# Intermolecular radical oxyalkylation of arynes with alkenes and TEMPO†

**DOI:** 10.1039/d4sc04369c

**Published:** 2024-07-26

**Authors:** Debkanta Bhattacharya, Maximilian Scherübl, Constantin G. Daniliuc, Armido Studer

**Affiliations:** a Organisch-Chemisches Institut, Universität Münster Corrensstraße 40 48149 Münster Germany studer@uni-muenster.de

## Abstract

Radical transformations with arynes represent an underexplored research field and only a few examples have been disclosed. In this research article, the implementation of arynes in three-component reactions with TEMPO (2,2,6,6-tetramethylpiperidine 1-oxyl) and activated alkenes is demonstrated. TEMPO is added to arynes, which triggers a Meerwein-type arylation cascade where the final alkyl radial is eventually trapped by a second equivalent of TEMPO. This method is applicable to activated alkenes such as electron-deficient acrylates, styrenes and also vinyl acetate to provide various bisalkoxyamines. This work is a contribution to the emerging field of radical aryne chemistry.

## Abstract 2

The implementation of arynes as acceptors in three-component radical reactions with TEMPO (2,2,6,6-tetramethylpiperidine 1-oxyl) and activated alkenes is demonstrated.

## Graphical abstract

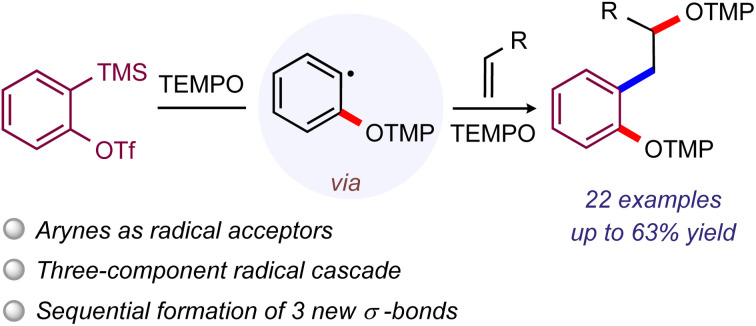

## Introduction

**Scheme 1:**
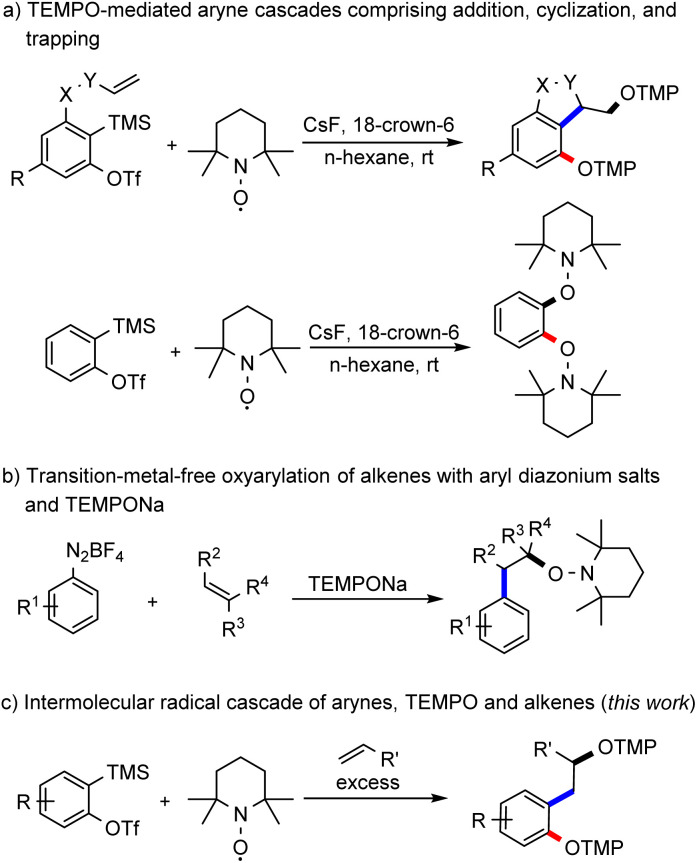
TEMPO-mediated strategies with intermediate aryl radicals. TMP: 2,2,6,6-tetramethylpiperidine.

Arynes are an interesting class of organic intermediates and versatile building blocks.^1^ They are highly reactive due to their low-lying lowest-unoccupied molecular orbital (LUMO), which renders them primarily electrophilic.^2^ Along with classical nucleophilic reactions, they are also known to engage in pericyclic^3^ and metal-mediated transformations.^4^ Its low-energy LUMO qualifies an aryne as a highly efficient radical acceptor. Despite this property, examples of arynes acting as radical acceptors are very rare.^5^ Along these lines, we recently successfully demonstrated that arynes can act as radical acceptors and we implemented various radical cascades such as direct TEMPO-trapping, radical cyclization and intramolecular HAT reactions (Scheme 1a).^6^ Recently, the Garg group has studied the reactivity of the TEMPO radical towards 1,2-cyclohexadiene.^7^ Earlier reported radical reactions comprising arynes as acceptors are exclusively intramolecular in nature and the realization of intermolecular radical cascade reactions with arynes is still a highly challenging task in synthesis. The fundamental problem is the generally high reactivity of the two intermediates that is acceptor aryne and the radical species that both have to be present in sufficiently high concentrations in the reaction medium. As demonstrated for the intramolecular variant, TEMPO owing to its persistent radical behaviour^8^ can be used as a reaction partner that is present in a larger concentration in combination with a highly reactive aryne intermediate.

The well-explored Meerwein arylation is a method that enables the formation of functionalized arenes through aryl radical addition to alkenes.^9^ The aryl radical is usually generated from diazonium salts by reduction with metal salts or from aryl halides by atom-transfer reactions. Other methods rely on hypervalent iodine compounds or aryl boronic acids as starting materials.^10^ In 2007, Heinrich and colleagues showed the successful addition of aryl radicals, generated by reduction of diazonium salts with iron sulfate, to alkenes with subsequent trapping of the adduct alkyl radical with TEMPO.^11^ Alternatively, our group used TEMPONa as the reducing agent and then *in situ* formed TEMPO acts as a radical scavenger of the adduct alkyl radical (Scheme 1b).^12^ We envisioned generating the aryl radical for a Meerwein-type arylation through a radical addition of TEMPO to an aryne (Scheme 1c). The transient aryl radical would then add to an alkene and the thus generated alkyl radical would be finally trapped by a second equivalent of TEMPO.

The main problem with this transformation is the competing direct TEMPO-trapping of the aryl radical, as observed in our previous study (Scheme 1a).^6^ Since the coupling of C-centred radicals with TEMPO is highly efficient, we envisaged using a large excess of the alkene component to steer the reaction towards the desired oxyalkylation.^10*a*,13^

## Results and discussion

**Scheme 2:**
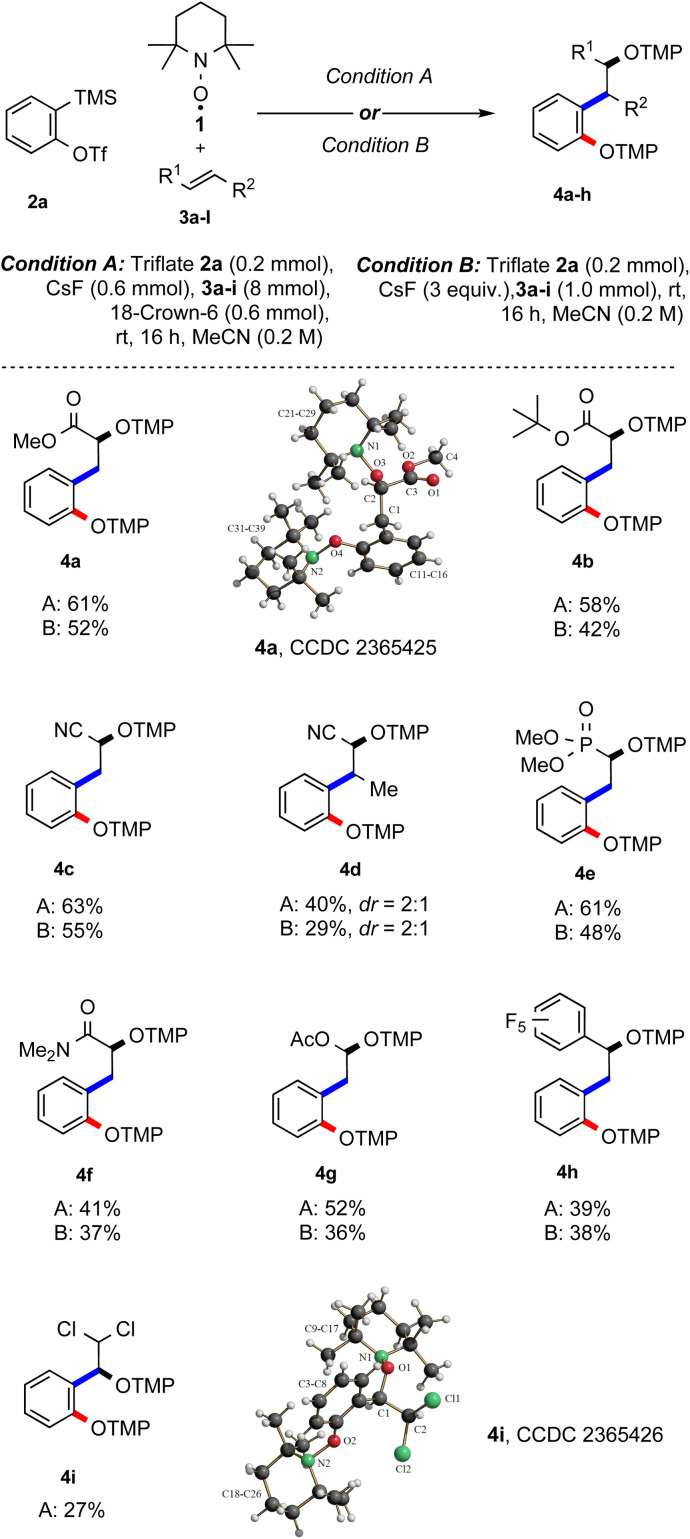
Substrate scope – variation of activated alkenes. Conditions: triflate 2a (0.20 mmol), TEMPO (1, 0.20 mmol), alkene 3a–h (8 mmol or 1 mmol), CsF (0.60 mmol), 18-crown-6 ether (0.60 mmol) and MeCN (1.0 mL, 0.20 M). Yield is calculated based on half the amount of TEMPO.

We started our investigations with triflate 2a in combination with TEMPO (1, 5 equiv.) and methyl acrylate (3a) under Kobayashi's conditions with CsF in the presence of 18-crown-6 ether in acetonitrile.^14^ By using 80 equivalents of 3a the desired bisalkoxyamine 4a was obtained in encouraging 36% yield (Table 1, entry 1). The structure of this compound was unambiguously confirmed by X-ray crystallography (see Scheme 2).^15^

**Table 1:** Reaction optimization^a^

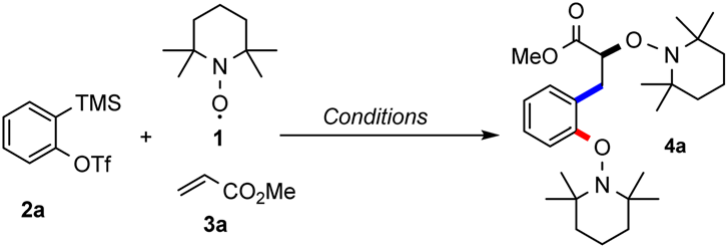					
Entry^a^	Aryne [mmol]	TEMPO [mmol]	Alkene 3a [mmol]	Solvent	Yield^b^^,^^e^
1	0.2	1.0	16	MeCN	37%
2	0.2	1.0	8	MeCN	27%
3	0.2	1.0	24	MeCN	47%
**4**	**0.2**	**0.2**	**8**	**MeCN**	**61%**
5	0.2	0.2	12	MeCN	63%
6	0.3	0.2	8	MeCN	61%
7^c^	0.2	0.2	8	MeCN	59%
8	0.2	0.2	8	Neat alkene	63%
9	0.2	0.2	8	THF	38%
10	0.2	0.2	8	*n*-Hexane	59%
11	0.2	0.2	8	CH_2_Cl_2_	53%
**12** d	**0.2**	**0.2**	**1**	**MeCN**	**52%**
13	0.2	0.2	1	MeCN	52%

a Reaction conditions: triflate 2a (0.20 mmol), TEMPO (1.0 mmol), CsF (0.60 mmol), methyl acrylate (3a, 16 mmol), 18-crown-6 ether (0.60 mmol), solvent (1.0 mL, 0.20 M), rt, 16 h.

b Isolated yield.

c 1 (M) solvent conc.

d Without 18-crown-6.

e Yield is calculated based on half the amount of TEMPO.

Decreasing the alkene concentration to 40 equivalents led to a lowering of the yield (27%, entry 2) and upon increasing the excess of 3a to 120 equivalents the yield increased to 47% (entry 3). In order to minimize competing double TEMPO addition (see Scheme 1a), the nitroxide radical was tested as the limiting reagent. Pleasingly, this variation of the stoichiometry increased the yield significantly to 61% by using 40 equivalents of 3a (entry 4). Increasing the methyl acrylate concentration (60 equiv.) had a slightly positive effect on the yield (63%, entry 5). With 1.5 equivalents of the aryne precursor the bisalkoxyamine 4a was formed in similar yield (entry 6). Surprisingly, increasing the overall concentration to 1.0 M or using the alkene as the neat solvent did not have a significant impact on the outcome of the reaction (entry 7 and 8). Lastly, different solvents were screened, but only *n*-hexane proved to be a viable alternative to acetonitrile to provide the desired product in 59% yield (entry 10). The reaction worked less efficiently in THF and CH_2_Cl_2_ where the yield decreased to 38% and 53%, respectively (entry 9 and 11).

To increase the atom economy of the transformation, we lowered the alkene excess to 5 equivalents applying a 1 : 1 molar ratio of the aryne precursor and TEMPO. The yield of the desired radical cascade product did not decrease significantly and 4a was obtained in 52% yield (entry 12). Note that this experiment was run in the absence of crown ether, while the same yield was achieved with crown ether (entry 13). Taken together, the best result was achieved with a one-to-one ratio of aryne precursor 2a and TEMPO and 40 equiv. (hereafter, condition A) or 5 equiv. (condition B) of alkene 3a in acetonitrile (entry 4 & 12). With optimised conditions in hand, the feasibility of the reaction with different alkenes was investigated, keeping triflate 2a as the benzyne precursor using either condition A or both (Scheme 2). Electron-poor alkenes were mostly considered, since electron-rich double bonds are known to undergo facile [2 + 2]-cycloaddition with arynes.^16^

In this three-component radical cascade reaction, both methyl (3a) and *tert*-butyl acrylate (3b) performed well and the corresponding bisalkoxyamines 4a and 4b were obtained in good yields applying condition A (61% and 58%) and slightly lower yields using condition B (52% and 42%). The electron-poorer acrylonitrile (3c) provided bisalkoxyamine 4c in a good yield (63%, condition A), while under condition B, the yield of 4c slightly dropped to 55%. As expected, yield decreased with the sterically more hindered crotononitrile (3d) and the desired product 4d was obtained in 40% yield as a 2 : 1 inseparable diastereoisomeric mixture (condition A); while applying condition B, the bisalkoxyamine 4d was formed with 29% yield. Next, vinyl phosphonate 3e was tested and alkoxyamine 4e was obtained in moderate to good yield using the two different methods (61%, A; 48%, B). *N*,*N*-Dimethylacrylamide (3f) afforded the targeted product 4f in moderate yield (41%, A; 37%, B). Surprisingly, the electron-rich vinyl acetate (3g) could also be implemented in this multicomponent reaction and the acetal 4g was isolated in 52% and 36% yield using conditions A and B, respectively. Pentafluorostyrene 3h engaged in the TEMPO-mediated radical cascade reaction to afford 4h in 39% yield (A). A similar yield was noted by applying condition B (38%). When (*E*)-1,2-dichloroethene (3i) was subjected to the optimized conditions, the unexpected product 4i was isolated in 27% yield. The structure of 4i was assigned by X-ray analysis.^15^ A possible explanation of this unexpected result is the occurrence of a formal intramolecular 1,2-migration of chlorine after initial aryl radical addition likely *via* Cl-radical fragmentation followed by re-addition, generating the more stable benzylic radical and subsequent TEMPO trapping.^17^

**Scheme 3:**
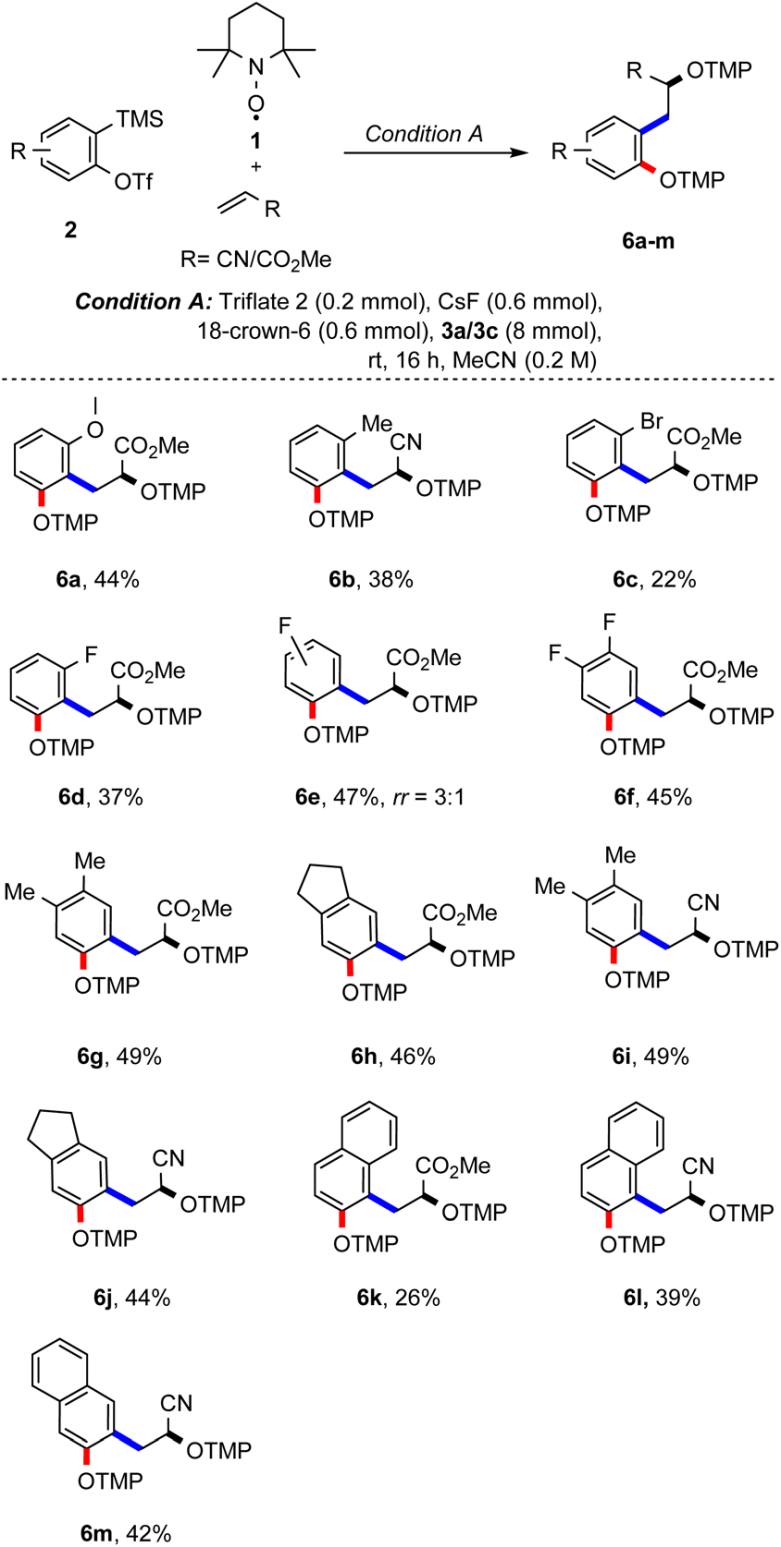
Substrate scope – variation of the aryne precursor. Conditions: triflate 5a–m (0.20 mmol), TEMPO (1, 0.20 mmol), alkene 3a or 3c (8 mmol), CsF (0.60 mmol), 18-crown-6 ether (0.60 mmol) and MeCN (1.0 mL, 0.20 M). Yield is calculated based on half the amount of TEMPO.

Studies were continued by testing different aryne precursors in the reaction with methyl acrylate or acrylonitrile using condition A (Scheme 3). *ortho*-Methoxy and *ortho*-methyl groups at the aryne moiety are tolerated in the reaction with 3a and the corresponding products 5a and 5b were formed, albeit with lower yields (44% and 38%), which can be rationalized by steric effects. Steric effects are even more pronounced for the *ortho*-bromo-substituted aryne generated from 5c, with the desired product 6c being obtained in only 22%. A slightly better yield was achieved for the smaller *ortho*-fluoro derivative 5d, which yielded the desired radical cascade product 6d in 37%. Importantly, in both cases the product was formed with complete regiocontrol. In contrast, the *meta*-fluoro substituted aryne 5e afforded the bisalkoxyamine 6e as a mixture of regioisomers (3 : 1) in 47% yield. Furthermore, 3,4-difluorobenzyne precursor 5f engaged in this cascade to furnish 6f in 45% yield.

Substrates 5g and 5h carrying alkyl substituents at the arene moiety were next examined in the reaction with methyl acrylate and the corresponding bisalkoxyamines 6g and 6h could be successfully prepared. Of note, inseparable side products resulting from competing [2 + 2] cycloaddition of methyl acrylate (3a) with the aryne were formed in these two transformations (see the ESI†).^16*c*^ When substrates 5g and 5h were reacted with acrylonitrile (3c), the desired products 6i and 6j were obtained in 49% and 44% yield, respectively. Similarly, the aryne precursor with a naphthalene core (see 5k) engaged in the reaction with both methyl acrylate and acrylonitrile to afford 6k and 6l in 26% and 39% yield with complete regiocontrol. Using the aryne precursor 5m containing a symmetrical naphthalene moiety in the reaction with acrylonitrile, 42% of the desired bisalkoxyamine 6m could be isolated.

**Scheme 4:**
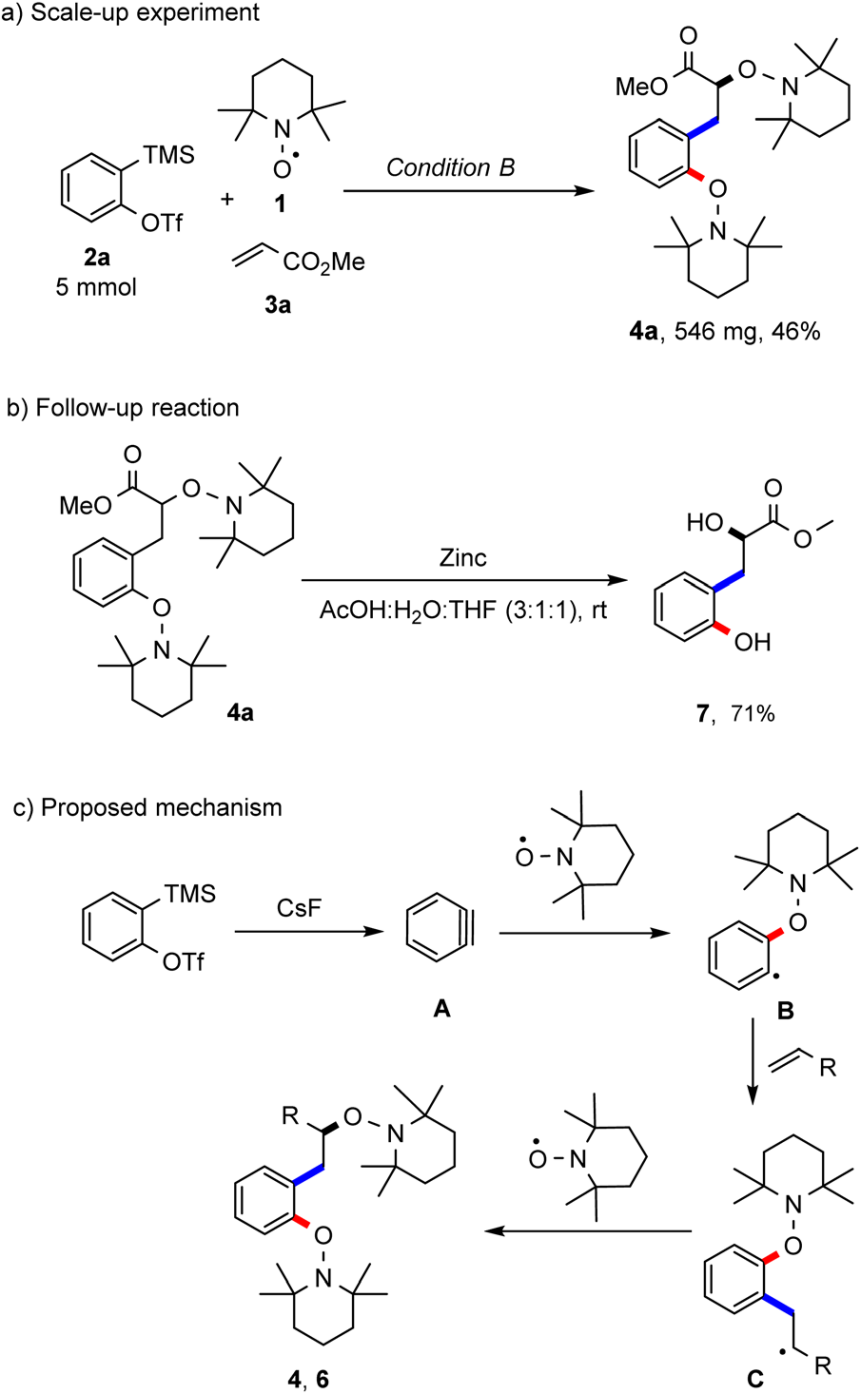
Scale-up experiment, follow up reaction and mechanistic proposal. Reaction conditions: (a) bisalkoxyamine 4a (0.10 mmol, 1.0 equiv.), zinc (1.2 mmol, 12 equiv.), AcOH : H_2_O : THF (3 : 1 : 1, 4.0 mL, 0.025 M).

To document robustness of this radical cascade, the reaction of the aryne precursor 2a, TEMPO and methyl acrylate was repeated applying condition A at a 5 mmol scale and the desired bisalkoxyamine 4a was obtained in 46% yield (Scheme 4a). The product bisalkoxyamines can be subjected to further chemical functionalisation. For example, the N–O bonds in bisalkoxyamine 4a could be reductively cleaved with zinc under acidic conditions to afford the corresponding hydroxyalkyl phenol 7 in a good yield (71%, Scheme 4b). The suggested mechanism of this novel radical cascade reaction is pictured in Scheme 4c. The generated transient aryne A acts as a radical acceptor in the reaction with the persistent TEMPO to generate an aryl radical B, which then undergoes a Meerwein-arylation to form an alkyl radical intermediate C. Then, a selective radical cross coupling between intermediate C and TEMPO (*k* = 10^8^–10^9^ M^−1^ s^−1^)^13*b*^ steered by the persistent radical effect (PRE)^8^ finally affords the isolated products 4 or 6. An excess of the alkene component is required in order to suppress the direct TEMPO trapping of the intermediate radical B.

## Conclusions

In this study, we developed intermolecular oxyalkylation of arynes using alkenes and TEMPO. This cascade reaction expands the scope of radical aryne chemistry by employing TEMPO, an alkene, and arynes as the three reaction components. The cascade involves formation of three new σ-bonds. To minimize competing side reactions, an excess of the alkene component is necessary. Additionally, we successfully implemented the reduction of the O–N bond of the alkoxyamines as a subsequent reaction.

## Data availability

Crystallographic data: deposition numbers 2365425 (4a) and 2365426 (4i) contain the ESI† crystallographic data for this paper. Experimental procedures and analytical data (NMR, MS, IR, and melting points) can be found in the ESI.† Copies of NMR spectra are also provided.

## Author contributions

D. B. and M. S. conducted all experiments and characterized all novel compounds. C. G. D. measured and solved the X-ray crystal structures. D. B., M. S. and A. S. designed the experiments and wrote the manuscript.

## Conflicts of interest

There are no conflicts to declare.

## Supplementary Material

SC-015-D4SC04369C-s001

SC-015-D4SC04369C-s002
