# Supplementary material for: Intermolecular radical oxyalkylation of arynes with alkenes and TEMPO
Source: Chem Sci. 2024 Jul 26;15(34):13712–6. doi: 10.1039/d4sc04369c (PMC11351772; doi:10.1039/d4sc04369c)
Supplement: SC-015-D4SC04369C-s001 [file SC-015-D4SC04369C-s001.pdf]

Supplementary Information (SI) for Chemical Science.
This journal is © The Royal Society of Chemistry 2024

Supporting Information

Intermolecular radical oxyalkylation of arynes with alkenes and TEMPO

Debkanta Bhattacharya[1,2], Maximilian Scherübl[1,2], Constantin G. Daniliuc[1] and Armido Studer[1,*]

1. Organisch-Chemisches Institut, Universität Münster, Corrensstraße 40, 48149 Münster, Germany

***Email: studer@uni-muenster.de**

1. General information……………………………………………………………………
2. Preparation of starting materials……………………………………………………………
3. General procedures (GP1& GP2)…………………………………………………………..
4. Physical data of the products………………………………………………………………..
5. Procedure for large scale experiment……………………………………………………..
6. Failed substrates……………………………………………………………………………
7. Crystallographic data………………………………………………………………………
8. References……………………………………………………………………………….
9. NMR spectra of the products……………………………………………………………

1. General information:

All reactions that are air and moisture sensitive were performed in oven-heated glassware under argon atmosphere by using Schlenk-technique. Anhydrous tetrahydrofuran (THF) was refluxed over elemental Na and freshly distilled from K metal before use. Anhydrous dichloromethane (CH_2Cl_2) was dried over P_4O_{10} and freshly distilled before use. All reagents were purchased from Sigma Aldrich, Acros Organics, ABCR, TCI, Alfa Aesar, BLDPharm and Fluorochem and were used without any further purification. Thin layer chromatography (TLC) was performed on Merck silica gel 60 F-254 plates and visualized by fluorescence quenching under UV light or staining with KMnO4 (1.5 g in 400 mL H_2O, 5 g $NaHCO_3$). Solvents for column chromatography were purchased in technical grade and purified by distillation prior to use. Column chromatography was performed on Merck or VWR silica gel 60 (40- 63 μm) using a compress air pressure of 0.2 bar. ^{1}H NMR, ^{13}C NMR and ^{19}F NMR spectra were measured on DPX 300, AV 400 or 500 at 300 K and chemical shift (δ) is expressed in ppm unit. Coupling constants were reported in Hertz (Hz), singlet is defined as s; broad singlet as brs; doublet as d; triplet as t; quartet as q; doublet of doublet as dd; triplet of triplet as tt; multiplet as m. HRMS (ESI-MS) spectra were measured on a Thermo Fisher Scientific LTQ XL Orbitrap and Thermo Fisher Scientific Orbitrap Velos Pro spectrometer. Infrared spectra (IR) were measured on a Jasco FT/IR-4600 spectrometer and bands are given by wavenumber (cm^{-1}). Melting points were measured using a Büchi Melting Point M-560.

2. Preparation of starting materials:

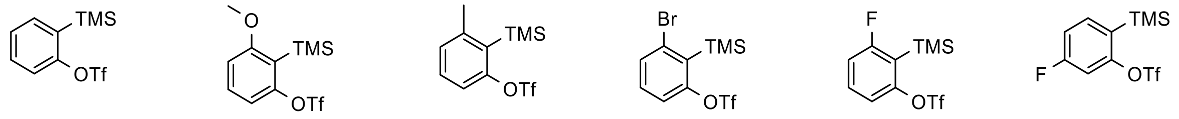

2a **5a** **5b** **5c** **5d** **5e**

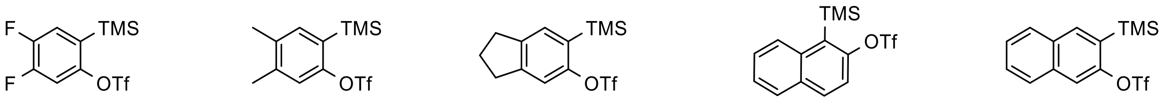

5f **5g** **5h** **5k** **5m**

Substrates **2a, 5a, 5b & 5c** are commercially available and directly used without further purifications.

Aryne precursors **5d-m** were synthesised from the corresponding *o*-bromophenol derivatives according to the reported procedure as followed [1,2,3]:

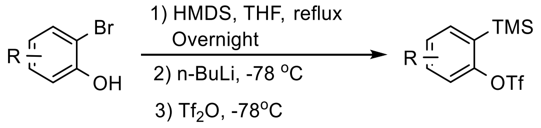

A solution of substituted o-bromophenol (4.0 mmol, 1.0 equiv) and hexamethyl disilylamine (HMDS) (1.7 mL, 8.0 mmol, 2.0 equiv) in THF (5 mL) was heated under refluxing condition overnight. The resulting solution was then concentrated directly on a rotary evaporator and further dried under vacuum for one hour. The crude material was dissolved in anhydrous THF (30 mL) and cooled to -78 ^{o}C under inert atmosphere. n-BuLi (1.6 M in hexanes, 3.75 mL, 6.00 mmol, 1.5 equiv) was added dropwise at the same temperature. After 30 minutes, triflic anhydride (Tf_2O) (1.0 mL, 6.0 mmol, 1.5 equiv) was added. After 20 minutes, 10% aq $NaHCO_3$ (30 mL) was added. The resulting mixture was extracted with pet ether (30 mL × 3). The combined organic layers were washed with brine (20 mL), dried over Na_2SO_4, filtered, and concentrated. Flash column chromatography with pet ether afforded aryne precursors **5d-m**.

3. General procedures:

General procedure for condition A (GP1):

A 10 mL oven-dried Schlenk tube equipped with a magnetic stirring bar was subjected to three cycles of vacuum/argon backfill, and charged with aryne precursor 2-(trimethylsilyl)phenyl trifluoromethanesulfonate (49 µL, 0.20 mmol, 1.0 equiv.), TEMPO (31.2 mg, 0.20 mmol, 1.0 equiv.), CsF (91 mg, 0.60 mmol, 3.0 equiv.), 18-crown-6 ether (159 mg, 0.600 mmol, 3.00 equiv.) and methyl acrylate (0.73 mL, 8.0 mmol, 40 equiv.) which were dissolved in dry MeCN (1.0 mL, 0.20 M). The reaction mixture was stirred at room temperature for 16 h under argon atmosphere. The solvent and excess of alkene were removed in a rotary evaporator under reduced pressure and the residue was subjected to flash column chromatography over silica gel to give the corresponding product.

General procedure for condition B (GP2):

A 10 mL oven-dried Schlenk tube equipped with a magnetic stirring bar was subjected to three cycles of vacuum/argon backfill, and charged with aryne precursor 2-(trimethylsilyl)phenyl trifluoromethanesulfonate (49 µL, 0.20 mmol, 1.0 equiv.), TEMPO (31.2 mg, 0.20 mmol, 1.0 equiv.), CsF (91 mg, 0.60 mmol, 3.0 equiv.) and methyl acrylate (91 µL, 1.0 mmol, 5 equiv.) which were dissolved in dry MeCN (1.0 mL, 0.20 M). The reaction mixture was stirred at room temperature for 16 h under argon atmosphere. The solvent and excess of alkene were removed in a rotary evaporator under reduced pressure and the residue was subjected to flash column chromatography over silica gel to give the corresponding product.

4. Physical data of the products:

Methyl 2-((2,2,6,6-tetramethylpiperidin-1-yl)oxy)-3-(2-((2,2,6,6-tetramethylpiperidin-1-yl)oxy)phenyl)propanoate (4a)

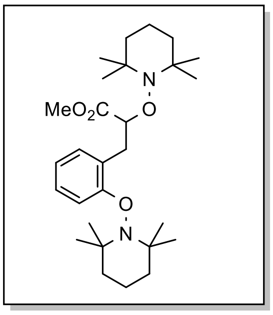

The reaction was performed according to the GP1 with 2-(trimethylsilyl)phenyl trifluoromethanesulfonate (49 µL, 0.20 mmol, 1.0 equiv.), TEMPO (31.2 mg, 0.20 mmol, 1.0 equiv.), CsF (91 mg, 0.60 mmol, 3.0 equiv.), 18-crown-6 ether (159 mg, 0.600 mmol, 3.00 equiv.) and methyl acrylate (0.73 mL, 8.0 mmol, 40 equiv.). After purification by flash chromatography (with SiO_2, gradient of Et_2O in *n*-pentane = 1% to 2%), the desired compound **4a** was obtained as a white solid (29 mg, 0.061 mmol, 61%).

The reaction was performed according to the GP2 with 2-(trimethylsilyl)phenyl trifluoromethanesulfonate (49 µL, 0.20 mmol, 1.0 equiv.), TEMPO (31.2 mg, 0.20 mmol, 1.0 equiv.), CsF (91 mg, 0.60 mmol, 3.0 equiv.) and methyl acrylate (91 µL, 1.0 mmol, 5 equiv.). After purification by flash chromatography (with SiO_2, gradient of Et_2O in *n*-pentane = 1% to 2%), the desired compound **4a** was obtained as a white solid (24.6 mg, 0.052 mmol, 52%).

^{1}H-NMR (500 MHz, C_6D_6): δ (ppm) 7.64 (dd, *J* = 8.4, 1.2 Hz, 1H), 7.19 (dd, *J* = 7.4, 1.7 Hz, 1H), 7.09 (ddd, *J* = 8.3, 7.3, 1.7 Hz, 1H), 6.76 (td, *J* = 7.4, 1.2 Hz, 1H), 5.07 (dd, *J* = 9.3, 7.0 Hz, 1H), 3.66 (dd, *J* = 12.9, 7.0 Hz, 1H), 3.22 (s, 3H), 3.15 (dd, *J* = 12.9, 9.3 Hz, 1H), 1.56 – 1.42 (m, 7H), 1.40 – 1.26 (m, 20H), 1.22 (s, 3H), 1.12 (s, 3H), 1.02 (s, 3H).

^{13}C-NMR (126 MHz, C_6D_6): δ (ppm) 172.9, 161.8, 131.9, 121.0, 120.2, 114.6, 85.4, 60.7, 60.60, 60.60, 59.8, 40.8, 40.7, 40.13, 40.07, 34.7, 34.3, 33.4, 32.7, 32.4, 21.5, 21.1, 20.6, 20.4, 17.5, 17.4.

HR-MS (ESI): calc. for $C_{28}H_{47}N_2O_4$ $[M+H]^+$ 475.3530 found 475.3530.

FTIR (neat): ν (cm^{-1}) 3002, 2974, 2928, 2866, 2363, 1739, 1582, 1469, 1441, 1362, 1173, 1165, 1133, 1014, 972, 875, 750.

T_{melt}: 94 – 96 °C

***tert*-Butyl 2-((2,2,6,6-tetramethylpiperidin-1-yl)oxy)-3-(2-((2,2,6,6-tetramethylpiperidin-1-yl)oxy)phenyl)propanoate (4b)**

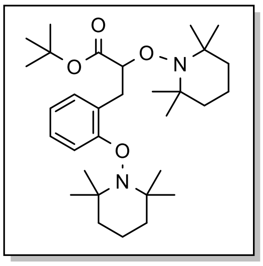

The reaction was performed according to the GP1 with 2-(trimethylsilyl)phenyl trifluoromethanesulfonate (49 µL, 0.20 mmol, 1.0 equiv.), TEMPO (31.2 mg, 0.20 mmol, 1.0 equiv.), CsF (91 mg, 0.60 mmol, 3.0 equiv.), 18-crown-6 ether (159 mg, 0.600 mmol, 3.00 equiv.) and *tert*-butyl acrylate (1.17 mL, 8.0 mmol, 40 equiv.). After purification by flash chromatography (with SiO_2, gradient of Et_2O in *n*-pentane = 2%), the desired compound **4b** was obtained as a white solid (30 mg, 0.058 mmol, 58%).

The reaction was performed according to the GP2 with 2-(trimethylsilyl)phenyl trifluoromethanesulfonate (49 µL, 0.20 mmol, 1.0 equiv.), TEMPO (31.2 mg, 0.20 mmol, 1.0 equiv.), CsF (91 mg, 0.60 mmol, 3.0 equiv.) and *tert*-butyl acrylate (146.5 µL, 1.0 mmol, 5 equiv.). After purification by flash chromatography (with SiO_2, gradient of Et_2O in *n*-pentane = 2%), the desired compound **4b** was obtained as a white solid (24.7 mg, 0.042 mmol, 42%).

^{1}H-NMR (599 MHz, C_6D_6): δ (ppm) 7.63 (dd, *J* = 8.3, 1.2 Hz, 1H), 7.24 (dd, *J* = 7.4, 1.7 Hz, 1H), 7.10 (ddd, *J* = 8.6, 7.3, 1.7 Hz, 1H), 6.75 (td, *J* = 7.3, 1.2 Hz, 1H), 4.93 (dd, *J* = 9.4, 6.9 Hz, 1H), 3.67 (dd, *J* = 12.8, 6.9 Hz, 1H), 3.12 (dd, *J* = 12.8, 9.4 Hz, 1H), 1.70 – 1.06 (m, 42H), 1.01 (s, 3H).

^{13}C-NMR (75 MHz, C_6D_6): δ (ppm) 172.2, 162.0, 132.7, 127.8, 121.3, 120.2, 114.4, 85.3, 80.2, 60.59, 60.57, 60.5, 59.8, 40.81, 40.80, 40.12, 40.05, 35.4, 34.5, 34.3, 32.8, 32.6, 28.1, 28.0, 21.6, 21.3, 20.6, 20.4, 17.6, 17.4.

HR-MS (ESI): calc. for $C_{31}H_{53}N_2O_4$ $[M+H]^+$ 517.4000 found 517.4004.

FTIR (neat): ν (cm^{-1}) 2969, 2932, 2869, 2363, 1736, 1585, 1479, 1453, 1363, 1217, 1148, 931, 846, 743.

T_{melt}: 105 – 107 °C.

2-((2,2,6,6-Tetramethylpiperidin-1-yl)oxy)-3-(2-((2,2,6,6-tetramethylpiperidin-1-yl)oxy)phenyl)propanenitrile (4c)

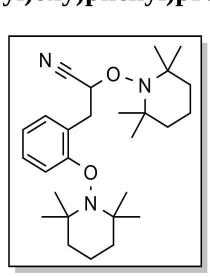

The reaction was performed according to the GP1 with 2-(trimethylsilyl)phenyl trifluoromethanesulfonate (49 µL, 0.20 mmol, 1.0 equiv.), TEMPO (31.2 mg, 0.20 mmol, 1.0 equiv.), CsF (91 mg, 0.60 mmol, 3.0 equiv.), 18-crown-6 ether (159 mg, 0.600 mmol, 3.00 equiv.) and acrylonitrile (0.52 mL, 8.0 mmol,

40 equiv.). After purification by flash chromatography (with SiO_2, gradient of Et_2O in *n*-pentane = 2%), the desired compound **4c** was obtained as a white solid (28 mg, 0.063 mmol, 63%).

The reaction was performed according to the GP2 with 2-(trimethylsilyl)phenyl trifluoromethanesulfonate (49 µL, 0.20 mmol, 1.0 equiv.), TEMPO (31.2 mg, 0.20 mmol, 1.0 equiv.), CsF (91 mg, 0.60 mmol, 3.0 equiv.) and acrylonitrile (65.5 µL, 1.0 mmol, 5 equiv.). After purification by flash chromatography (with SiO_2, gradient of Et_2O in *n*-pentane = 2%), the desired compound **4c** was obtained as a white solid (24.3 mg, 0.051 mmol, 51%).

^{1}H-NMR (500 MHz, C_6D_6): δ (ppm) 7.60 (dd, *J* = 8.4, 1.2 Hz, 1H), 7.16 (s, 1H), 7.11 (ddd, *J* = 8.3, 7.3, 1.7 Hz, 1H), 6.79 (td, *J* = 7.4, 1.2 Hz, 1H), 5.11 (dd, *J* = 7.5, 6.9 Hz, 1H), 3.29 – 3.20 (m, 2H), 1.60 (s, 3H), 1.54 – 1.23 (m, 24H), 1.20 (s, 3H), 1.19 (s, 3H), 1.13 (s, 3H), 1.06 (s, 3H), 1.03 (s, 3H), 1.00 (s, 3H), 0.96 (s, 3H).

^{13}C-NMR (126 MHz, C_6D_6): δ (ppm) 161.2, 131.9, 128.6, 120.5, 120.1, 119.6, 74.3, 61.1, 60.7, 60.6, 60.2, 40.3, 40.1, 40.0, 39.9, 35.0, 34.5, 34.0, 32.7, 21.2, 21.0, 20.7, 20.5, 17.3, 17.2.

HR-MS (ESI): calc. for $C_{27}H_{43}KN_3O_2$ $[M+K]^+$ 480.2987 found 480.2985**.**

FTIR (neat): ν (cm^{-1}) ν 2972, 2931, 2359, 1739, 1604, 1585, 1479, 1455, 1378, 1363, 1260, 1247, 1218, 1107, 1043, 992, 955, 929, 738.

T_{melt}: 105 – 107 °C.

2-((2,2,6,6-Tetramethylpiperidin-1-yl)oxy)-3-(2-((2,2,6,6-tetramethylpiperidin-1-yl)oxy)phenyl)butanenitrile (4d)

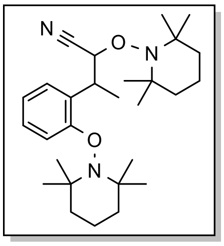

The reaction was performed according to the GP1 with 2-(trimethylsilyl)phenyl trifluoromethanesulfonate (49 µL, 0.20 mmol, 1.0 equiv.), TEMPO (31.2 mg, 0.20 mmol, 1.0 equiv.), CsF (91 mg, 0.60 mmol, 3.0 equiv.), 18-crown-6 ether (159 mg, 0.600 mmol, 3.00 equiv.) and cis and trans mixture of crotononitrile (651.4 µL, 8.0 mmol, 40 equiv.). After purification by flash chromatography (with SiO_2, gradient of Et_2O in *n*-pentane = 1%), the desired compound **4d** was obtained as a colorless liquid (18.3 mg, 0.040 mmol, 40%).

The reaction was performed according to the GP2 with 2-(trimethylsilyl)phenyl trifluoromethanesulfonate (49 µL, 0.20 mmol, 1.0 equiv.), TEMPO (31.2 mg, 0.20 mmol, 1.0 equiv.), CsF (91 mg, 0.60 mmol, 3.0 equiv.) and cis and trans mixture of crotononitrile (81.5 µL, 1.0 mmol, 5 equiv.). After purification by flash chromatography (with SiO_2, gradient of Et_2O in *n*-pentane = 1%), the desired compound **4d** was obtained as a colorless liquid (13.2 mg, 0.029 mmol, 29%).

^{1}H NMR (400 MHz, $CDCl_3$, as a mixture of diastereomers, 2:1) δ 7.57 – 7.48 (m, 1H), 7.28 – 7.17 (m, 1H), 7.12 – 7.04 (m, 1H), 6.86 – 6.74 (m, 1H), 4.93 – 4.83 (m, 1H), 4.10 – 3.53 (m, 1H), 1.64 – 1.46 (m, 8H), 1.45 – 1.33 (m, 7H), 1.33 – 1.23 (m, 5H), 1.23 – 1.14 (m, 7H), 1.09 – 0.94 (m, 7H), 0.93 – 0.71 (m, 5H).

^{13}C NMR (101 MHz, $CDCl_3$, 400 MHz, $CDCl_3$, as a mixture of diastereomers, 2:1): δ 160.1, 160, 128.1, 127.8, 127.7, 127.6, 125.7, 125.5, 124.4, 120.3, 120.0, 119.9, 118.5, 114.7, 79.1, 75.6, 61.4, 60.82, 60.79, 60.7, 60.65, 60.6, 60.5, 60.4, 60.2, 40.3, 40.2, 40.17, 40.14, 40.1, 40.0, 39.9(5), 39.9(0), 34.3, 34.2, 34.1, 33.8, 33.70, 32.69, 32.51, 32.48, 21.6, 21.4, 21.3, 21.1, 20.9, 20.6, 20.5, 17.1(8), 17.1(7), 17.1(3), 15.0, 14.9.

HR-MS (ESI): calc. for $C_{28}H_{45}N_3O_2Na$ $[M+Na]^+$ 478.3404 found 478.3405**.**

FTIR (neat): 2974, 2931, 2374, 2320, 1736, 1602, 1583, 1477, 1454, 1379, 1260, 1232, 1179, 1131, 1085, 990, 1011, 990, 956, 835, 753, 719.

Dimethyl (1-((2,2,6,6-tetramethylpiperidin-1-yl)oxy)-2-(2-((2,2,6,6-tetramethylpiperidin-1-yl)oxy)phenyl)ethyl)phosphonate (4e)

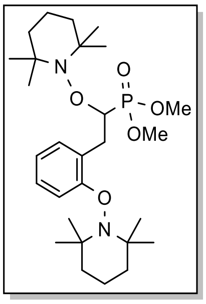

The reaction was performed according to the GP1 with 2-(trimethylsilyl)phenyl trifluoromethanesulfonate (49 µL, 0.20 mmol, 1.0 equiv.), TEMPO (31.2 mg, 0.20 mmol, 1.0 equiv.), CsF (91 mg, 0.60 mmol, 3.0 equiv.), 18-crown-6 ether (159 mg, 0.600 mmol, 3.00 equiv.) and dimethyl vinylphosphonate (0.95 mL, 8.0 mmol, 40 equiv.). After purification by flash chromatography (with SiO_2, gradient of Et_2O in *n*-pentane = 35% to 50%), the desired compound **4e** was obtained as a colorless liquid (32 mg, 0.061 mmol, 61%).

The reaction was performed according to the GP2 with 2-(trimethylsilyl)phenyl trifluoromethanesulfonate (49 µL, 0.20 mmol, 1.0 equiv.), TEMPO (31.2 mg, 0.20 mmol, 1.0 equiv.), CsF (91 mg, 0.60 mmol, 3.0 equiv.) and dimethyl vinylphosphonate (118.8 µL, 1.0 mmol, 5 equiv.). After purification by flash chromatography (with SiO_2, gradient of Et_2O in *n*-pentane = 35% to 50%), the desired compound **4e** was obtained as a white solid (25.2 mg, 0.048 mmol, 48%).

^{1}H-NMR (599 MHz, C_6D_6): δ (ppm) 7.66 (dd, *J* = 8.3, 1.3 Hz, 1H), 7.54 (dd, *J* = 7.5, 1.7 Hz, 1H), 7.11 (ddd, *J* = 8.6, 7.3, 1.7 Hz, 1H), 6.84 (td, *J* = 7.4, 1.3 Hz, 1H), 4.99 (td, *J* = 9.7, 5.8 Hz, 1H), 3.94 (ddd, *J* = 30.3, 13.4, 5.8 Hz, 1H), 3.48 (d, *J* = 10.6 Hz, 3H), 3.47 – 3.43 (m, 2H), 3.27 (d, *J* = 10.7 Hz, 3H), 1.62 – 1.15 (m, 36H).

^{13}C-NMR (151 MHz, C_6D_6): δ (ppm) 161.6, 132.2, 127.3, 122.6, 122.6, 120.1, 114.7, 102.9, 80.4, 79.4, 60.7, 60.5, 52.54, 52.50, 51.52, 51.47, 40.9, 40.1, 35.1, 34.0, 32.7, 32.6, 32.2, 21.7, 21.2, 17.7, 17.4.

^{31}P-NMR (243 MHz, C_6D_6): δ (ppm) 25.71.

HR-MS (ESI): calc. for $C_{28}H_{49}KN_2O_5P$ $[M+K]^+$ 563.3011 found 563.3010**.**

FTIR (neat): ν (cm^{-1}) 2971, 2929, 2848, 2360, 1739, 1479, 1454, 1377, 1363, 1249, 1217, 1179, 1131, 1034, 829, 783, 751.

***N,N*-Dimethyl-2-((2,2,6,6-tetramethylpiperidin-1-yl)oxy)-3-(2-((2,2,6,6-tetramethylpiperidin-1-yl)oxy)phenyl)propanamide (4f)**

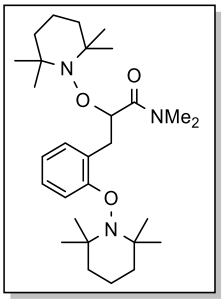

The reaction was performed according to the GP1 with 2-(trimethylsilyl)phenyl trifluoromethanesulfonate (49 µL, 0.20 mmol, 1.0 equiv.), TEMPO (31.2 mg, 0.20 mmol, 1.0 equiv.), CsF (91 mg, 0.60 mmol,

3.0 equiv.), 18-crown-6 ether (159 mg, 0.600 mmol, 3.00 equiv.) and *N,N*-dimethyl acrylamide (0.82 mL, 8.0 mmol, 40 equiv.). After purification by flash chromatography (with SiO_2, gradient of Et_2O in *n*-pentane = 10% to 20%), the desired compound **4f** was obtained as a white solid (20 mg, 0.041 mmol, 41%).

The reaction was performed according to the GP2 with 2-(trimethylsilyl)phenyl trifluoromethanesulfonate (49 µL, 0.20 mmol, 1.0 equiv.), TEMPO (31.2 mg, 0.20 mmol, 1.0 equiv.), CsF (91 mg, 0.60 mmol, 3.0 equiv.) and *N,N*-dimethyl acrylamide (103 µL, 1.0 mmol, 5 equiv.). After purification by flash chromatography (with SiO_2, gradient of Et_2O in *n*-pentane = 10% to 20%), the desired compound **4f** was obtained as a white solid (18 mg, 0.037 mmol, 37%).

^{1}H NMR (599 MHz, C_6D_6) δ 7.63 (dd, *J* = 8.4, 1.2 Hz, 1H), 7.34 (dd, *J* = 7.5, 1.7 Hz, 1H), 7.08 (ddd, *J* = 8.6, 7.3, 1.7 Hz, 1H), 6.78 (td, *J* = 7.4, 1.2 Hz, 1H), 5.17 (dd, *J* = 8.7, 6.6 Hz, 1H), 3.66 (dd, *J* = 12.9, 6.6 Hz, 1H), 3.39 (dd, *J* = 12.9, 8.8 Hz, 1H), 2.70 (s, 3H), 2.63 (s, 3H), 1.54 – 1.40 (m, 6H), 1.38 – 1.34 (m, 3H), 1.33 (s, 4H), 1.30 (s, 3H), 1.28 – 1.23 (m, 8H), 1.20 (s, 3H), 1.19 – 1.10 (m, 3H), 1.07 (s, 3H), 1.02 (s, 3H).

^{13}C-NMR (151 MHz, C_6D_6): δ (ppm) 172.3, 161.7, 132.4, 127.6, 122.0, 120.3, 114.8, 81.2, 60.8, 60.7, 60.5, 59.8, 41.0, 40.8, 40.14, 40.12, 37.0, 35.4, 34.3, 33.7, 33.3, 32.8, 32.7, 21.4, 21.2, 20.7, 20.5, 17.6, 17.4.

HR-MS (ESI): calc. for $C_{29}H_{49}KN_3O_3$ $[M+K]^+$ 526.3406 found 526.3405**.**

FTIR (neat): ν (cm^{-1}) 3000, 2970, 2938, 2366, 1739, 1642, 1429, 1407, 1244, 1207, 1133, 1098, 1059, 995, 973, 790.

T_{melt}: >107 °C decomposition.

1-((2,2,6,6-Tetramethylpiperidin-1-yl)oxy)-2-(2-((2,2,6,6-tetramethylpiperidin-1-yl)oxy)phenyl)ethyl acetate (4g)

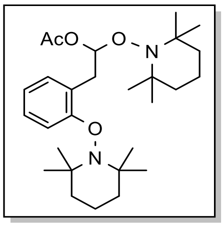

The reaction was performed according to the GP1 with 2-(trimethylsilyl)phenyl trifluoromethanesulfonate (49 µL, 0.20 mmol, 1.0 equiv.), TEMPO (31.2 mg, 0.20 mmol, 1.0 equiv.), CsF (91 mg, 0.60 mmol, 3.0 equiv.), 18-crown-6 ether (159 mg, 0.600 mmol, 3.00 equiv.) and dimethyl vinylacetate (0.74 mL, 8.0 mmol, 40 equiv.). After purification by flash chromatography (with SiO_2, gradient of Et_2O in *n*-pentane = 2%), the desired compound **4g** was obtained as a white solid (25 mg, 0.052 mmol, 52%).

The reaction was performed according to the GP2 with 2-(trimethylsilyl)phenyl trifluoromethanesulfonate (49 µL, 0.20 mmol, 1.0 equiv.), TEMPO (31.2 mg, 0.20 mmol, 1.0 equiv.), CsF (91 mg, 0.60 mmol, 3.0 equiv.) and dimethyl vinylacetate (92.2 µL, 1.0 mmol, 5 equiv.). After purification by flash chromatography (with SiO_2, gradient of Et_2O in *n*-pentane = 2%), the desired compound **4g** was obtained as a white solid (17.1 mg, 0.036 mmol, 36%).

^{1}H-NMR (599 MHz, C_6D_6): δ (ppm) 7.68 (dd, *J* = 8.3, 1.2 Hz, 1H), 7.26 (dd, *J* = 7.4, 1.7 Hz, 1H), 7.14 (ddd, *J* = 8.6, 7.3, 1.8 Hz, 1H), 6.90 (dd, *J* = 6.7, 5.9 Hz, 1H), 6.82 (td, *J* = 7.4, 1.2 Hz, 1H), 3.32 (dd, *J* = 13.5, 6.6 Hz, 1H), 3.25 (dd, *J* = 13.4, 5.9 Hz, 1H), 1.70 (s, 3H), 1.61 – 1.30 (m, 19H), 1.23 (s, 3H), 1.22 (s, 3H), 1.21 – 1.14 (m, 2H), 1.11 (s, 3H), 1.10 (s, 3H), 1.07 (s, 3H).

^{13}C-NMR (151 MHz, C_6D_6): δ (ppm) 168.9, 161.8, 132.6, 128.0, 120.8, 120.1, 115.0, 99.9, 61.1, 60.8, 60.6, 59.4, 40.7, 40.3, 40.23, 40.16, 35.5, 34.3, 33.8, 32.8, 32.7, 21.3, 21.2, 21.1, 20.7, 20.4, 17.5, 17.4.

HR-MS (ESI): calc. for $C_{28}H_{46}KN_2O_4$ $[M+K]^+$ 513.3089 found 513.3091**.**

FTIR (neat): ν (cm^{-1}) 2970, 2937, 2361, 1736, 1482, 1453, 1362, 1261, 1232, 1208, 1176, 1110, 1038, 967, 936, 751.

T_{melt}: 105 - 107 °C.

2,2,6,6-Tetramethyl-1-(2-(2-(perfluorophenyl)-2-((2,2,6,6-tetramethylpiperidin-1-yl)oxy)ethyl)phenoxy)piperidine (4h)

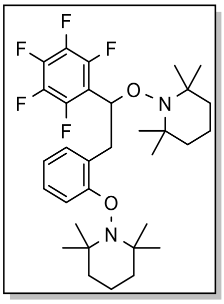

The reaction was performed according to the GP1 with 2-(trimethylsilyl)phenyl trifluoromethanesulfonate (49 µL, 0.20 mmol, 1.0 equiv.), TEMPO (31.2 mg, 0.20 mmol, 1.0 equiv.), CsF (91 mg, 0.60 mmol, 3.0 equiv.), 18-crown-6 ether (159 mg, 0.600 mmol, 3.00 equiv.) and pentafluoro styrene (1.1 mL, 8.0 mmol, 40 equiv.). After purification by flash chromatography (with SiO_2, gradient of Et_2O in *n*-pentane = 1%), the desired compound **4h** was obtained as a white solid (23 mg, 0.039 mmol, 39%).

The reaction was performed according to the GP2 with 2-(trimethylsilyl)phenyl trifluoromethanesulfonate (49 µL, 0.20 mmol, 1.0 equiv.), TEMPO (31.2 mg, 0.20 mmol, 1.0 equiv.), CsF (91 mg, 0.60 mmol, 3.0 equiv.) and pentafluoro styrene (138 µL, 1.0 mmol, 5 equiv.). After purification by flash chromatography (with SiO_2, gradient of Et_2O in *n*-pentane = 1%), the desired compound **4h** was obtained as a white solid (22.1 mg, 0.038 mmol, 38%).

^{1}H-NMR (599 MHz, C_6D_6): δ (ppm) 7.59 (dd, *J* = 8.4, 1.2 Hz, 1H), 7.00 (ddd, *J* = 8.5, 7.3, 1.7 Hz, 1H), 6.95 (dd, *J* = 7.4, 1.7 Hz, 1H), 6.65 (td, *J* = 7.4, 1.2 Hz, 1H), 5.79 (dd, *J* = 9.8, 6.5 Hz, 1H), 3.91 (dd, *J* = 13.1, 6.6 Hz, 1H), 3.42 (dd, *J* = 13.1, 9.7 Hz, 1H), 1.56 – 1.25 (m, 19H), 1.20 (s, 3H), 1.19 – 1.12 (m, 2H), 1.11 (s, 3H), 1.08 (s, 3H), 0.93 (s, 3H), 0.76 (s, 3H).

^{13}C NMR (151 MHz, C_6D_6, ^{1}H/^{19}F decoupled) δ 161.55, 145.35, 140.72, 137.76, 131.13, 128.35, 128.10, 127.98, 121.35, 120.40, 117.12, 115.20, 78.82, 67.08, 60.69, 60.60, 60.58, 59.83, 40.73, 40.49, 40.11, 40.02, 34.87, 34.68, 33.63, 32.52, 21.33, 20.99, 20.58, 20.41, 17.47, 17.29.

^{19}F-NMR (564 MHz, C_6D_6): δ (ppm) -143.28 (dd, *J* = 23.3, 8.3 Hz), -143.80, -144.37 (m), -153.99 (t, *J* = 21.6 Hz), -154.81 (t, *J* = 21.3 Hz), -155.08, -156.55 (m), -161.69 (td, *J* = 22.6, 8.3 Hz), -162.02, -162.64 (m).

HR-MS (ESI): calc. for $C_{32}H_{44}F_5N_2O_2$ $[M+H]^+$ 583.3317 found 583.3320**.**

FTIR (neat): ν (cm^{-1}) 2975, 2932, 1657, 1604, 1520, 1479, 1454, 1362, 1304, 1248, 1216, 1179, 1122, 1000, 956, 885, 752.

T_{melt}: 113-115 °C.

1-(2-(2,2-Dichloro-1-((2,2,6,6-tetramethylpiperidin-1-yl)oxy)ethyl)phenoxy)-2,2,6,6-tetramethylpiperidine (6i)

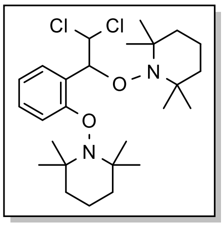

The reaction was performed according to the GP1 with 2-(trimethylsilyl)phenyl trifluoromethanesulfonate (49 µL, 0.20 mmol, 1.0 equiv.), TEMPO (31.2 mg, 0.20 mmol, 1.0 equiv.), CsF (91 mg, 0.60 mmol, 3.0 equiv.), 18-crown-6 ether (159 mg, 0.600 mmol, 3.00 equiv.) and trans-1,2-dichloroethylene (0.95 mL, 8.0 mmol, 40 equiv.). After purification by flash chromatography (with SiO_2, gradient of Et_2O in *n*-pentane = 1%), the desired compound **4i** was obtained as a white solid (13 mg, 0.027 mmol, 27%).

^{1}H-NMR (599 MHz, C_6D_6): δ (ppm) 7.79 (dd, *J* = 7.6, 1.7 Hz, 1H), 7.75 (dd, *J* = 8.5, 1.2 Hz, 1H), 7.16 (m, 1H), 6.89 (td, *J* = 7.5, 1.2 Hz, 1H), 6.47 (d, *J* = 3.8 Hz, 1H), 5.97 (s, 1H), 1.54 – 1.29 (m, 16H), 1.26 (m, 9H), 1.11 (s, 3H), 1.06 (s, 3H), 1.03 (m, 5H).

^{13}C-NMR (151 MHz, C_6D_6): δ (ppm) 160.8, 130.5, 129.4, 121.8, 119.9, 114.6, 82.0, 74.9, 60.8, 60.6, 56.6, 41.1, 40.7, 40.52, 40.49, 35.2, 35.0, 34.4, 32.5, 32.3, 27.5, 21.1, 21.0, 20.7, 20.4, 17.4, 17.2.

HR-MS (ESI): calc. for $C_{26}H_{42}KN_2O_2Cl_2$ $[M+K]^+$ 523.2255 found 523.2255**.**

FTIR (neat): ν (cm^{-1}) 2968, 2933, 2868, 2359, 1739, 1602, 1581, 1473, 1447, 1376, 1362, 1246, 1215, 1096, 1016, 915, 803.

T_{melt}: 112 - 114 °C.

Methyl 3-(2-methoxy-6-((2,2,6,6-tetramethylpiperidin-1-yl)oxy)phenyl)-2-((2,2,6,6-tetramethylpiperidin-1-yl)oxy)propanoate (6a)

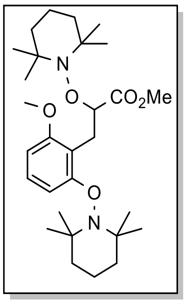

The reaction was performed according to the GP1 with 3-methoxy-2-(trimethylsilyl)phenyl trifluoromethanesulfonate **5a** (53 µL, 0.20 mmol, 1.0 equiv.), TEMPO (31.2 mg, 0.20 mmol, 1.0 equiv.), CsF (91 mg, 0.60 mmol, 3.0 equiv.), 18-crown-6 (159 mg, 0.600 mmol, 3.00 equiv.) and methyl acrylate (0.73 mL, 8.0 mmol, 40 equiv.). After purification by flash chromatography (with SiO_2, gradient of Et_2O in *n*-pentane = 3%), the desired compound **6a** was obtained as a colorless liquid (22 mg, 0.044 mmol, 44%).

^{1}H-NMR (599 MHz, C_6D_6): δ (ppm) 7.40 (d, *J* = 8.3 Hz, 1H), 7.06 (t, *J* = 8.3 Hz, 1H), 6.23 (d, *J* = 8.0 Hz, 1H), 5.18 (t, *J* = 8.0 Hz, 1H), 3.66 (d, *J* = 8.0 Hz, 2H), 3.39 (s, 3H), 3.29 (s, 3H), 1.61 – 1.08 (m, 36H).

^{13}C-NMR (151 MHz, C_6D_6): δ (ppm) 173.2, 162.7, 159.2, 127.5, 109.8, 108.2, 102.8, 84.7, 60.7, 60.6, 60.6, 59.7, 55.2, 50.6, 40.8, 40.23, 40.15, 34.2, 33.5, 32.63, 32.61, 27.1, 21.4, 21.2, 20.6, 20.3, 17.6, 17.4.

HR-MS (ESI): calc. for $C_{29}H_{49}N_2O_5$ $[M+H]^+$ 505.3636 found 505.3637**.**

FTIR (neat): ν (cm^{-1}) 2971, 2930, 2359, 1741, 1590, 1456, 1438, 1377, 1362, 1251, 1207, 1166, 1095, 1022, 993, 922, 777.

3-(2-Methyl-6-((2,2,6,6-tetramethylpiperidin-1-yl)oxy)phenyl)-2-((2,2,6,6-tetramethylpiperidin-1-yl)oxy)propanenitrile (6b)

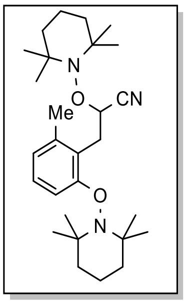

The reaction was performed according to the GP1 with 3-methyl-2-(trimethylsilyl)phenyl trifluoromethanesulfonate **5b** (50.8 µL, 0.20 mmol, 1.0 equiv.), TEMPO (31.2 mg, 0.20 mmol, 1.0 equiv.), CsF (91 mg, 0.60 mmol, 3.0 equiv.), 18-crown-6 ether (159 mg, 0.600 mmol, 3.00 equiv.) and acrylonitrile (0.52 mL, 8.0 mmol, 40 equiv.). After purification by flash chromatography (with SiO_2, gradient of Et_2O in *n*-pentane = 2%), the desired compound **6b** was obtained as a colorless liquid (17.3 mg, 0.038 mmol, 38%).

^{1}H NMR (300 MHz, $CDCl_3$) δ 7.41 (d, *J* = 7.8 Hz, 1H), 7.09 – 7.01 (m, 1H), 6.72 (d, *J* = 7.5 Hz, 1H), 5.02 (t, 1H), 3.26 (d, *J* = 7.5 Hz, 2H), 2.42 (s, 3H), 1.67 – 1.53 (m, 6H), 1.51 – 1.44 (m, 3H), 1.43 – 1.38 (m, 4H), 1.29 (s, 5H), 1.25 (s, 3H), 1.16 – 1.07 (m, 3H), 1.02 (s, 3H), 0.98 – 0.88 (m, 9H).

^{13}C NMR (101 MHz, $CDCl_3$) δ 161.1, 138.2, 127.4, 122.3, 120.1, 118.9, 112.6, 73.6, 61.0, 60.7, 60.4, 60.0, 40.1, 40.0, 39.9, 34.3, 33.9, 32.7, 32.5, 31.1, 21.4, 21.1, 20.8, 20.5, 20.2, 17.2, 17.1.

HR-MS (ESI): calc. for $C_{28}H_{45}N_3O_2H$ $[M+H]^+$ 456.3584 found 456.3576.

FTIR (neat): 2975, 2932, 2372, 1581, 1463, 1378, 1254, 1209, 1132, 1065, 1045, 992, 877, 778, 713.

Methyl 3-(2-bromo-6-((2,2,6,6-tetramethylpiperidin-1-yl)oxy)phenyl)-2-((2,2,6,6-tetra-methylpiperidin-1-yl)oxy)propanoate (6c)

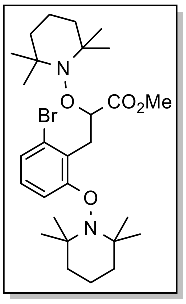

The reaction was performed according to the GP1 with 3-bromo-2-(trimethylsilyl)phenyl trifluoromethanesulfonate **5c** (75.5 µL, 0.20 mmol, 1.0 equiv.), TEMPO (31.2 mg, 0.20 mmol, 1.0 equiv.), CsF (91 mg, 0.60 mmol, 3.0 equiv.), 18-crown-6 (159 mg, 0.600 mmol, 3.00 equiv.) and methyl acrylate (0.73 mL, 8.0 mmol, 40 equiv.). After purification by flash chromatography (with SiO_2, gradient of Et_2O in *n*-pentane = 3%), the desired compound **6c** was obtained as a colorless liquid (12 mg, 0.022 mmol, 22%).

^{1}H NMR (599 MHz, C_6D_6) δ 7.53 (dd, *J* = 8.4, 1.2 Hz, 1H), 7.11 (dd, *J* = 8.0, 1.1 Hz, 1H), 6.76 (t, *J* = 8.2 Hz, 1H), 5.20 (dd, *J* = 8.7, 6.7 Hz, 1H), 3.89 – 3.81 (m, 1H), 3.50 (dd, *J* = 13.5, 6.7 Hz, 1H), 3.35 (s, 3H), 1.49 – 1.33 (m, 7H), 1.32 – 1.26 (m, 10H), 1.25 (s, 3H), 1.23 (s, 3H), 1.18 (s, 3H), 1.16 – 1.05 (m, 3H), 1.03 (s, 3H), 1.00 – 0.93 (m, 4H).
^{13}C-NMR (151 MHz, C_6D_6): δ (ppm) 172.6, 162.8, 128.4, 126.6, 124.9, 122.5, 114.1, 84.2, 61.0, 60.7, 60.6, 59.9, 50.8, 40.8, 40.7, 40.2, 40.1, 34.5, 33.9, 33.4, 32.5, 32.4, 21.4, 21.2, 20.64, 20.58, 17.5, 17.2.

HR-MS (ESI): calc. for $C_{28}H_{46}N_2O_4Br$ $[M+H]^+$ 553.2636 found 553.2639**.**

FTIR (neat): ν (cm^{-1}) 3006, 2972, 2930, 2360, 1742, 1589, 1568, 1435, 1376, 1362, 1277, 1238, 1209, 1043, 1022, 924, 877, 704.

Methyl 3-(2-fluoro-6-((2,2,6,6-tetramethylpiperidin-1-yl)oxy)phenyl)-2-((2,2,6,6-tetramethylpiperidin-1-yl)oxy)propanoate (6d)

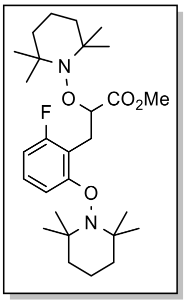

The reaction was performed according to the GP1 with 3-fluoro-2-(trimethylsilyl)phenyl trifluoromethanesulfonate **5d** (63.3 μL, 0.20 mmol, 1.0 equiv.), TEMPO (31.2 mg, 0.20 mmol, 1.0 equiv.), CsF (91 mg, 0.60 mmol, 3.0 equiv.), 18-crown-6 (159 mg, 0.600 mmol, 3.00 equiv.) and methyl acrylate (0.73 mL, 8.0 mmol, 40 equiv.). After purification by flash chromatography (with SiO_2, gradient of Et_2O in *n*-pentane = 2%), the desired compound **6d** was obtained as a colorless liquid (18.2 mg, 0.037 mmol, 37%).

^{1}H NMR (599 MHz, C_6D_6) δ 7.38 (d, *J* = 8.5 Hz, 1H), 6.91 – 6.84 (m, 1H), 6.55 (t, *J* = 8.7 Hz, 1H), 5.11 (t, *J* = 8.0 Hz, 1H), 3.55 (dd, *J* = 13.2, 7.5 Hz, 1H), 3.43 (dd, *J* = 13.2, 8.6 Hz, 1H), 3.31 (s, 3H), 1.51 – 1.40 (m, 6H), 1.37 – 1.24 (m, 18H), 1.20 (s, 3H), 1.16 – 1.07 (m, 2H), 1.06 – 1.02 (m, 5H), 0.99 – 0.81 (m, 2H).

^{13}C-NMR (151 MHz, C_6D_6, ^{1}H/^{19}F-decoupled): δ (ppm) 172.3, 162.5, 162.1, 110.2, 109.2, 106.7, 84.3, 60.4, 60.34, 60.27, 59.4, 50.4, 40.4, 40.3, 39.7, 39.7, 33.9, 33.0, 32.1, 32.0, 26.3, 21.0, 20.7, 20.1, 19.9, 17.1, 16.9.

^{19}F-NMR (282 MHz, C_6D_6): δ (ppm) -116.20.

HR-MS (ESI): calc. for $C_{28}H_{46}N_2O_4F$ $[M+H]^+$ 493.3436 found 493.3437**.**

FTIR (neat): ν (cm^{-1}) 2971, 2933, 2359, 1743, 1656, 1457, 1364, 1217, 1130, 1038, 958, 782.

Methyl 3-(3/4-fluoro-6-((2,2,6,6-tetramethylpiperidin-1-yl)oxy)phenyl)-2-((2,2,6,6-tetramethylpiperidin-1-yl)oxy)propanoate (6e)

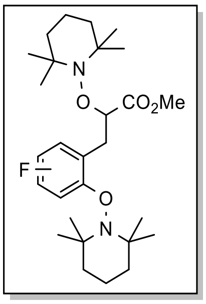

The reaction was performed according to the GP1 with 4-fluoro-2-(trimethylsilyl)phenyl trifluoromethanesulfonate **5e** (63.3 μL, 0.20 mmol, 1.0 equiv.), TEMPO (31.2 mg, 0.20 mmol, 1.0 equiv.), CsF (91 mg, 0.60 mmol, 3.0 equiv.), 18-crown-6 (159 mg, 0.600 mmol, 3.00 equiv.) and methyl acrylate (0.73 mL, 8.0 mmol, 40 equiv.). After purification by flash chromatography (with SiO_2, gradient of Et_2O in *n*-pentane = 2%), the desired compound **6e** was obtained as a white solid (23.2 mg, 0.047 mmol, rr= 3:1 as determined by ^{1}H NMR, 47%).

^{1}H NMR (400 MHz, $CDCl_3$, containing two regioisomers) δ 7.46 (dd, *J* = 9.0, 4.9 Hz, 1H), 6.82 – 6.72 (m, 2H), 4.69 (dd, *J* = 9.1, 7.0 Hz, 1H), 3.48 (s, 3H), 3.40 (dd, *J* = 13.0, 7.0 Hz, 1H), 2.81 (dd, *J* = 13.1, 9.1 Hz,

1H), 1.68 – 1.52 (m, 6H), 1.46 – 1.38 (m, 5H), 1.30 – 1.23 (m, 8H), 1.20 – 1.08 (m, 9H), 1.05 – 0.99 (m, 6H), 0.94 (s, 3H).

^{13}C NMR (101 MHz, $CDCl_3$, containing two regioisomers) δ 173.2, 157.4, 157.4, 157.3, 155.0, 121.9, 121.8, 117.5, 117.3, 115.1, 115.0, 113.6, 113.4, 84.6, 60.6(1), 60.6(0), 60.5, 59.6, 51.1, 40.5, 40.4, 40.1, 40.0, 34.0, 33.9, 33.8, 33.1, 32.5, 32.2, 21.4, 21.1, 20.4, 20.2, 17.3, 17.2.

^{19}F NMR (376 MHz, $CDCl_3$) δ -126.07 (for both regioisomers)

HR-MS (ESI): calc. for $C_{28}H_{46}N_2O_4F$ $[M+H]^+$ 493.3436 found 493.3438**.**

FTIR (neat): ν (cm^{-1}) 2978, 2930, 2871, 1746, 1481, 1377, 1177, 1132, 1020, 995, 928, 716.

T_{melt}: 88 – 91 °C.

Methyl 3-(4,5-difluoro-2-((2,2,6,6-tetramethylpiperidin-1-yl)oxy)phenyl)-2-((2,2,6,6-tetramethylpiperidin-1-yl)oxy)propanoate (6f)

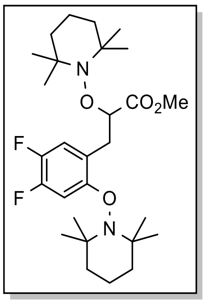

The reaction was performed according to the GP1 with 4,5-difluoro-2-(trimethylsilyl)phenyl trifluoromethanesulfonate **5f** (67 µL, 0.20 mmol, 1.0 equiv.), TEMPO (31.2 mg, 0.20 mmol, 1.0 equiv.), CsF (91 mg, 0.60 mmol, 3.0 equiv.), 18-crown-6 (159 mg, 0.600 mmol, 3.00 equiv.) and methyl acrylate (0.73 mL, 8.0 mmol, 40 equiv.). After purification by flash chromatography (with SiO_2, gradient of Et_2O in *n*-pentane = 2%), the desired compound **6f** was obtained as a white solid (23 mg, 0.045 mmol, 45%).

^{1}H-NMR (500 MHz, C_6D_6): δ (ppm) 7.54 (dd, *J* = 13.1, 7.4 Hz, 1H), 6.92 (dd, *J* = 10.7, 8.8 Hz, 1H), 4.91 (dd, *J* = 9.2, 6.9 Hz, 1H), 3.41 (dd, *J* = 13.2, 6.9 Hz, 1H), 3.19 (s, 3H), 2.90 (dd, *J* = 13.2, 9.2 Hz, 1H), 1.45 – 1.06 (m, 30H), 0.98 (s, 3H), 0.90 (s, 3H).

^{13}C-NMR (126 MHz, C_6D_6): δ (ppm) 172.5, 157.9, 149.7, 144.3, 119.5, 116.9, 104.3, 84.9, 60.8, 60.7, 59.8, 40.7, 40.6, 39.8, 39.7, 34.2, 33.5, 33.3, 32.4, 32.1, 21.4, 21.1, 20.5, 20.3, 17.5, 17.1.

^{19}F-NMR (470 MHz, C_6D_6): δ (ppm) -137.50 (d, *J* = 22.9 Hz), -149.60 (d, *J* = 22.9 Hz).

HR-MS (ESI): calc. for $C_{28}H_{45}N_2O_4F_2$ $[M+H]^+$ 511.3342 found 511.3345.

FTIR (neat): ν (cm^{-1}) 2972, 2932, 1744, 1479, 1377, 1363, 1244, 1232, 1205, 1164, 1133, 1020, 956, 923, 876, 738.

T_{melt}: 100 - 102 °C.

Methyl 3-(4,5-dimethyl-2-((2,2,6,6-tetramethylpiperidin-1-yl)oxy)phenyl)-2-((2,2,6,6-tetramethylpiperidin-1-yl)oxy)propanoate (6g) and methyl 3,4-dimethylbicyclo[4.2.0]octa-1(6),2,4-triene-7-carboxylate (6g’)

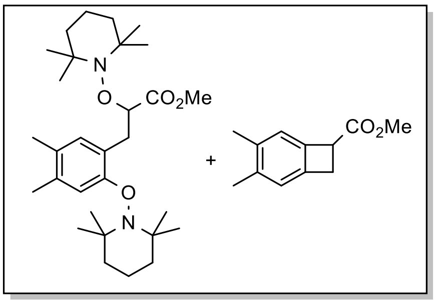

The reaction was performed according to the GP1 with 4,5-dimethyl-2-(trimethylsilyl)phenyl trifluoromethanesulfonate **5g** (65.3 µL, 0.20 mmol, 1.0 equiv.), TEMPO (31.2 mg, 0.20 mmol, 1.0 equiv.), CsF (91 mg, 0.60 mmol, 3.0 equiv.), 18-crown-6 (159 mg, 0.600 mmol, 3.00 equiv.) and methyl acrylate (0.73 mL, 8.0 mmol, 40 equiv.). After purification by flash chromatography (with SiO_2, gradient of Et_2O in *n*-pentane = 2%), the desired compound **6g** was obtained along with an inseparable mixture of **6g'** as colorless liquid (31 mg, **6g:6g'** = 1.44:1.00**,** 0.083 mmol, **6g:** 49% and **6g':** 34%).

For 6g:

^{1}H-NMR (599 MHz, C_6D_6): δ (ppm) 7.53 (s, 1H), 6.96 (s, 1H), 5.04 (dd, *J* = 9.4, 6.9 Hz, 1H), 3.61 (dd, *J* = 12.9, 6.8 Hz, 1H), 3.22 (s, 3H), 3.13 (dd, *J* = 13.0, 9.4 Hz, 1H), 2.03 (s, 3H), 2.00 (s, 3H), 1.56 – 1.04 (m, 36H).

^{13}C NMR (151 MHz, C_6D_6) δ 173.0, 159.9, 135.6, 133.1, 127.4, 118.0, 115.8, 85.7, 60.7, 60.6, 51.4, 50.5, 40.8, 40.7, 40.2, 40.1, 34.3, 34.2, 33.4, 32.9, 32.6, 21.5, 21.1, 20.3, 20.2, 20.1, 18.7, 17.5, 17.4.

HR-MS (ESI): calc. for $C_{30}H_{51}N_2O_4$ $[M+H]^+$ 503.3843 found 503.3846**.**

For 6g':

^{1}H-NMR (599 MHz, C_6D_6): δ (ppm) 6.90 (s, 1H), 6.65 (s, 1H), 4.05 (dd, *J* = 5.8, 2.6 Hz, 1H), 3.44 (dd, *J* = 13.8, 2.6 Hz, 1H), 3.32 (s, 3H), 3.09 (dd, *J* = 13.7, 5.5 Hz, 1H), 1.97 (t, *J* = 1.4 Hz, 6H).

^{13}C NMR (151 MHz, C_6D_6) δ 172.4, 142.0, 140.9, 136.6, 135.8, 124.2, 123.8, 59.7, 45.8, 33.4, 20.6, 20.3.

Methyl 2-((2,2,6,6-tetramethylpiperidin-1-yl)oxy)-3-(6-((2,2,6,6-tetramethylpiperidin-1-yl)oxy)-2,3-dihydro-1H-inden-5-yl)propanoate (6h) and methyl 2,4,5,6-tetrahydro-1*H*-cyclobuta[*f*]indene-1-carboxylate (6h')

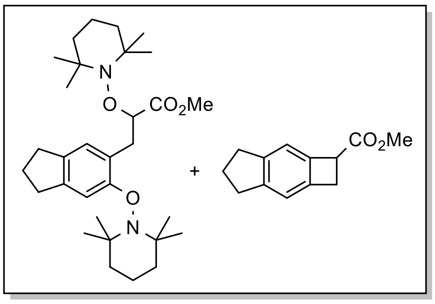

The reaction was performed according to the GP1 with 6-(trimethylsilyl)-2,3-dihydro-1H-inden-5-yl trifluoromethanesulfonate **5h** (67.7 µL, 0.20 mmol, 1.0 equiv.), TEMPO (31.2 mg, 0.20 mmol, 1.0 equiv.), CsF (91 mg, 0.60 mmol, 3.0 equiv.), 18-crown-6 (159 mg, 0.600 mmol, 3.00 equiv.) and methyl acrylate (0.73 mL, 8.0 mmol, 40 equiv.). After purification by flash chromatography (with SiO_2, gradient of Et_2O in *n*-pentane = 2%), the desired compound **6h** was obtained along with an inseparable mixture of **6h'** as colorless liquid (32.2 mg, **6h:6h'** = 1.10:1.00**,** 0.088 mmol, **6h:** 46% and **6g':** 42%).

For 6h:

^{1}H-NMR (599 MHz, C_6D_6): δ (ppm) 7.67 (s, 1H), 7.10 (s, 1H), 5.09 (dd, *J* = 9.2, 6.9 Hz, 1H), 3.70 – 3.65 (m, 1H), 3.27 (s, 3H), 3.22 (dd, *J* = 13.0, 9.2 Hz, 1H), 2.72 (dt, *J* = 24.3, 7.4 Hz, 4H), 1.77 (p, *J* = 7.3 Hz, 3H), 1.62 – 1.07 (m, 36H).

^{13}C NMR (151 MHz, C_6D_6) δ 173.0, 160.8, 143.6, 135.1, 127.4, 110.5, 85.7, 60.7, 60.6, 59.8, 50.6, 45.2, 40.7(9), 40.7(7), 40.2, 40.1, 34.7, 34.3, 33.4, 32.9, 32.6, 32.3, 25.7, 21.5, 21.2, 20.6, 20.3, 17.6, 17.4.

HR-MS (ESI): calc. for $C_{31}H_{51}N_2O_4$ $[M+H]^+$ 515.3843 found 515.3848**.**

For 6h':

^{1}H-NMR (599 MHz, C_6D_6): δ (ppm) 7.02 (s, 1H), 6.79 (s, 1H), 4.05 (ddd, *J* = 5.6, 2.6, 1.2 Hz, 1H), 3.45 (ddd, *J* = 13.7, 2.6, 1.1 Hz, 1H), 3.36 (s, 3H), 3.13 – 3.05 (m, 1H), 2.67 – 2.62 (m, 4H), 1.82 (p, *J* = 7.2 Hz, 2H).

^{13}C-NMR (151 MHz, C_6D_6): δ (ppm) 172.5, 143.9, 143.0, 142.0, 140.8, 119.5, 119.1, 51.4, 33.6, 33.4, 33.1, 26.0.

3-(4,5-Dimethyl-2-((2,2,6,6-tetramethylpiperidin-1-yl)oxy)phenyl)-2-((2,2,6,6-tetramethylpiperidin-1-yl)oxy)propanenitrile (6i)

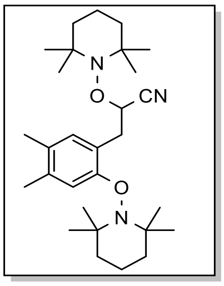

The reaction was performed according to the GP1 with 4,5-dimethyl-2-(trimethylsilyl)phenyl trifluoromethanesulfonate **5g** (65.3 µL, 0.20 mmol, 1.0 equiv.), TEMPO (31.2 mg, 0.20 mmol, 1.0 equiv.), CsF (91 mg, 0.60 mmol, 3.0 equiv.), 18-crown-6 ether (159 mg, 0.600 mmol, 3.00 equiv.) and acrylonitrile (0.52 mL, 8.0 mmol, 40 equiv.). After purification by flash chromatography (with SiO_2, gradient of Et_2O in *n*-pentane = 2%), the desired compound **6i** was obtained as a colorless oil (23 mg, 0.049 mmol, 49%).

^{1}H NMR (400 MHz, $CDCl_3$) δ 7.31 (s, 1H), 6.92 (s, 1H), 4.92 (t, *J* = 7.4 Hz, 1H), 3.25 (dd, *J* = 13.4, 7.2 Hz, 1H), 3.04 (dd, *J* = 13.4, 7.7 Hz, 1H), 2.21 (s, 3H), 2.15 (s, 3H), 1.69 – 1.54 (m, 5H), 1.52 – 1.41 (m, 5H), 1.38 (s, 3H), 1.35 – 1.22 (m, 8H), 1.14 (s, 3H), 1.09 (s, 3H), 1.05 (s, 3H), 1.03 – 0.94 (m, 6H).

^{13}C NMR (101 MHz, $CDCl_3$) δ 158.9, 136.3, 132.5, 127.6, 119.7, 116.3, 115.8, 74.3, 60.7, 60.5, 60.5, 60.1, 40.2, 40.1, 39.9, 39.9, 34.2, 34.1, 33.9, 32.7, 32.7, 21.3, 21.1, 20.6, 20.4, 20.2, 18.8, 17.2.

HR-MS (ESI): calc. for $C_{29}H_{47}N_3O_2Na$ $[M+Na]^+$ 492.3560 found 492.3556.

FTIR (neat): ν (cm^{-1}) 2972, 2930, 2373, 1736, 1593, 1460, 1378, 1249, 1219, 1132, 1045, 993, 743.

2-((2,2,6,6-Tetramethylpiperidin-1-yl)oxy)-3-(6-((2,2,6,6-tetramethylpiperidin-1-yl)oxy)-2,3-dihydro-1H-inden-5-yl)propanenitrile (6j)

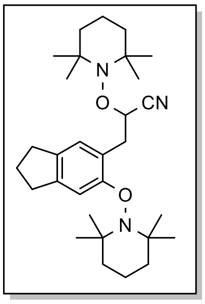

The reaction was performed according to the GP1 with 6-(trimethylsilyl)-2,3-dihydro-1H-inden-5-yl trifluoromethanesulfonate **5h** (67.7 µL, 0.20 mmol, 1.0 equiv.), TEMPO (31.2 mg, 0.20 mmol, 1.0 equiv.), CsF (91 mg, 0.60 mmol, 3.0 equiv.), 18-crown-6 ether (159 mg, 0.600 mmol, 3.00 equiv.) and acrylonitrile (0.52 mL, 8.0 mmol, 40 equiv.). After purification by flash chromatography (with SiO_2, gradient of Et_2O in *n*-pentane = 2%), the desired compound **6j** was obtained as a white solid (21.2 mg, 0.044 mmol, 44%).

^{1}H NMR (400 MHz, $CDCl_3$) δ 7.41 (s, 1H), 7.02 (s, 1H), 4.93 (t, *J* = 7.4 Hz, 1H), 3.27 (dd, *J* = 13.4, 7.2 Hz, 1H), 3.07 (dd, *J* = 13.4, 7.6 Hz, 1H), 2.89 – 2.76 (m, 4H), 2.11 – 1.96 (m, 2H), 1.72 – 1.61 (m, 3H), 1.60 – 1.46 (m, 8H), 1.38 (s, 3H), 1.31 – 1.20 (m, 7H), 1.14 (s, 3H), 1.08 (s, 3H), 1.05 (s, 3H), 1.03 – 0.93 (m, 6H).

^{13}C NMR (101 MHz, $CDCl_3$) δ 159.8, 144.3, 135.2, 126.9, 119.8, 117.0, 110.7, 74.4, 70.0, 60.5(6), 60.5(4), 60.1, 40.2, 40.1, 40.0, 39.9, 34.7, 34.2, 33.9, 33.4, 32.8, 32.7, 32.1, 25.8, 21.4, 21.1, 20.6, 20.4, 17.2.

HR-MS (ESI): calc. for $C_{30}H_{47}N_3O_2H$ $[M+H]^+$ 482.3741found 482.3740.

FTIR (neat): ν (cm^{-1}) 2972, 2930, 2320, 1735, 1470, 1378, 1261, 1183, 1132, 1054, 992, 927.

T_{melt}: 103 - 105 °C.

Methyl 2-((2,2,6,6-tetramethylpiperidin-1-yl)oxy)-3-(2-((2,2,6,6-tetramethylpiperidin-1-yl)oxy)naphthalen-1-yl)propanoate (6k)

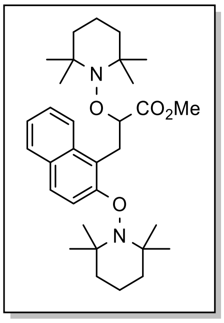

The reaction was performed according to the GP1 with 1-(trimethylsilyl)naphthalen-2-yl trifluoromethanesulfonate **5k** (69.7 µL, 0.20 mmol, 1.0 equiv.), TEMPO (31.2 mg, 0.20 mmol, 1.0 equiv.), CsF (91 mg, 0.60 mmol, 3.0 equiv.), 18-crown-6 (159 mg, 0.600 mmol, 3.00 equiv.) and methyl acrylate (0.73 mL, 8.0 mmol, 40 equiv.). After purification by flash chromatography (with SiO_2, gradient of Et_2O in *n*-pentane = 2%), the desired compound **6k** was obtained as a colorless liquid (14.4 mg, 0.026 mmol, 26%).

^{1}H NMR (300 MHz, $CDCl_3$) δ 8.16 (d, *J* = 8.7 Hz, 1H), 7.96 (d, *J* = 9.2 Hz, 1H), 7.76 – 7.60 (m, 2H), 7.50 – 7.37 (m, 1H), 7.33 – 7.21 (m, 1H), 4.85 (dd, *J* = 9.1, 6.4 Hz, 1H), 3.89 – 3.71 (m, 1H), 3.43 – 3.31 (m, 4H), 1.74 – 1.60 (m, 4H), 1.50 – 1.36 (m, 5H), 1.38 – 1.28 (m, 6H), 1.28 – 1.19 (m, 3H), 1.12 (s, 3H), 1.06 – 0.99 (m, 6H), 1.00 – 0.91 (m, 6H), 0.68 (s, 3H).

^{13}C NMR (76 MHz, $CDCl_3$) δ 173.7, 158.9, 133.8, 129.1, 128.1, 127.6, 125.8, 124.2, 122.7, 116.4, 113.5, 84.3, 60.8, 60.5, 60.4, 59.5, 51.2, 40.6, 40., 40.15, 40.08, 33.9, 33.2, 32.4, 32.3, 28.6, 21.5, 21.1, 20.4, 19.9, 17.2.

HR-MS (ESI): calc. for $C_{32}H_{48}N_2O_4H$ $[M+H]^+$ 525.3686 found 525.3687.

FTIR (neat): ν (cm^{-1}) 2971, 2930, 2871, 1742, 1622, 1593, 1513, 1459, 1377, 1249, 1154, 1062, 1039, 993, 818, 734.

2-((2,2,6,6-Tetramethylpiperidin-1-yl)oxy)-3-(2-((2,2,6,6-tetramethylpiperidin-1-yl)oxy)naphthalen-1-yl)propanenitrile (6l)

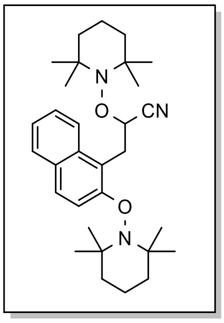

The reaction was performed according to the GP1 with 1-(trimethylsilyl)naphthalen-2-yl trifluoromethanesulfonate **5k** (69.7 µL, 0.20 mmol, 1.0 equiv.), TEMPO (31.2 mg, 0.20 mmol, 1.0 equiv.), CsF (91 mg, 0.60 mmol, 3.0 equiv.), 18-crown-6 (159 mg, 0.600 mmol, 3.00 equiv.) and acrylonitrile (0.52 mL, 8.0 mmol, 40 equiv.). After purification by flash chromatography (with SiO_2, gradient of Et_2O in *n*-pentane = 2%), the desired compound **6l** was obtained as a colorless liquid (19.2 mg, 0.039 mmol, 39%).

^{1}H NMR (400 MHz, $CDCl_3$) δ 8.05 (d, *J* = 8.6 Hz, 1H), 7.97 (d, *J* = 9.1 Hz, 1H), 7.78 – 7.67 (m, 2H), 7.48 (ddd, *J* = 8.4, 6.8, 1.4 Hz, 1H), 7.35 – 7.25 (m, 1H), 5.08 (dd, *J* = 8.6, 5.8 Hz, 1H), 3.77 – 3.62 (m, 2H), 1.83 – 1.46 (m, 11H), 1.35 (s, 3H), 1.32 (s, 3H), 1.29 – 1.19 (m, 4H), 1.11 (s, 3H), 1.08 (s, 3H), 0.95 (s, 3H), 0.85 (s, 3H), 0.58 (s, 3H).

^{13}C NMR (101 MHz, $CDCl_3$) δ 158.9, 133.6, 129.1, 128.5, 128.4, 126.3, 123.6, 123.1, 120.1, 116.5, 112.1, 73.5, 61.1, 61.0, 60.7, 60.0, 40.2, 40.1, 40.03, 39.98, 34.4, 33.7, 32.6, 32.3, 29.6, 21.5, 21.1, 20.8, 20.1, 17.2, 17.1.

HR-MS (ESI): calc. for $C_{31}H_{45}N_3O_2Na$ $[M+Na]^+$ 514.3404 found 514.3400.

FTIR (neat): ν (cm^{-1}) 2971, 2930, 2871, 1739, 1623, 1594, 1513, 1459, 1435, 1378, 1363, 1219, 1183, 1132, 1065, 819.

2-((2,2,6,6-Tetramethylpiperidin-1-yl)oxy)-3-(3-((2,2,6,6-tetramethylpiperidin-1-yl)oxy)naphthalen-2-yl)propanenitrile (6m)

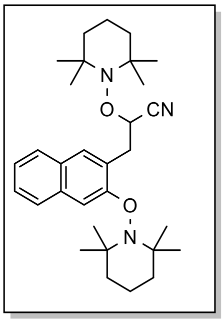

The reaction was performed according to the GP1 with 3-(trimethylsilyl)naphthalen-2-yl trifluoromethanesulfonate **5m** (69.7 µL, 0.20 mmol, 1.0 equiv.), TEMPO (31.2 mg, 0.20 mmol, 1.0 equiv.), CsF (91 mg, 0.60 mmol, 3.0 equiv.), 18-crown-6 (159 mg, 0.600 mmol, 3.00 equiv.) and acrylonitrile (0.52 mL, 8.0 mmol, 40 equiv.). After purification by flash chromatography (with SiO_2, gradient of Et_2O in *n*-pentane = 2%), the desired compound **6m** was obtained as a colorless liquid (20.7 mg, 0.042 mmol, 42%).

^{1}H NMR (400 MHz, CDCl$_3$) δ 7.85 (s, 1H), 7.72 (dd, *J* = 8.2, 4.5 Hz, 2H), 7.69 (s, 1H), 7.39 (ddd, *J* = 8.3, 6.8, 1.2 Hz, 1H), 7.29 (ddd, *J* = 8.0, 6.8, 1.2 Hz, 1H), 5.11 – 5.03 (m, 1H), 3.53 – 3.41 (m, 1H), 3.32 (dd, *J* = 13.6, 6.8 Hz, 1H), 1.79 – 1.56 (m, 7H), 1.55 – 1.43 (m, 5H), 1.41 (s, 3H), 1.33 (s, 3H), 1.30 (s, 3H), 1.16 (s, 3H), 1.09 – 0.99 (m, 9H), 1.00 (s, 3H).

^{13}C NMR (101 MHz, CDCl$_3$) δ 158.9, 134.2, 130.8, 128.6, 127.5, 126.7, 126.1, 123.5, 122.3, 119.5, 109.8, 73.9, 61.0, 60.8, 60.7, 60.2, 40.1, 40.03, 39.99, 39.9, 35.2, 34.2, 33.9, 32.8, 32.7, 21.4, 21.2, 20.7, 20.4, 17.2, 17.1.

HR-MS (ESI): calc. for $C_{31}H_{45}N_3O_2Na$ $[M+Na]^+$ 514.3404 found 514.3397.

FTIR (neat): 2974, 2930, 2352, 1633, 1602, 1503, 1456, 1373, 1260, 1183, 1089, 992, 925, 877, 717.

Methyl 2-hydroxy-3-(2-hydroxyphenyl)propanoate (7)

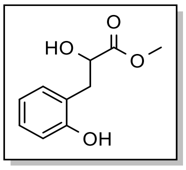

Bisalkoxyamine **4a** (47.5 mg, 0.100 mmol, 1.00 equiv.) was dissolved in AcOH:H_2O:THF (3:1:1, 4.0 mL, 0.025 M) and zinc dust (78 mg, 1.2 mmol, 12 equiv.) was added portionwise. After 4 h at room temperature the suspension was quenched by the addition of sat. aq. $NaHCO_3$ solution (10 mL). The aq. phase was extracted with CH_2Cl_2 (3×15 mL) and the combined organic layers were washed with sat. aq. NaCl solution (15 mL), dried over Na_2SO_4 and concentrated under reduced pressure. The desired product **7** was obtained as a colorless liquid (14 mg, 71%). The spectroscopic data are in accordance with those described in the literature[4].

^{1}H NMR (300 MHz, CDCl$_3$) δ 7.64 (s, 1H), 7.22 – 7.11 (m, 1H), 7.02 (dd, *J* = 7.6, 1.7 Hz, 1H), 6.92 (dd, *J* = 8.1, 1.2 Hz, 1H), 6.84 (td, *J* = 7.4, 1.3 Hz, 1H), 4.58 (dd, *J* = 6.0, 3.5 Hz, 1H), 3.80 (s, 3H), 3.78 (s, 1H), 3.27 (dd, *J* = 14.7, 3.6 Hz, 1H), 3.02 (dd, *J* = 14.7, 6.0 Hz, 1H).

^{13}C NMR (101 MHz, CDCl$_3$) δ 173.9, 155.8, 131.4, 129.2, 123.2, 120.7, 117.7, 72.3, 53.2, 35.9.

FTIR (neat): ν (cm^{-1}) 3346, 1729, 1610, 1584, 1505, 1490, 1457, 1440, 1363, 1227, 1176, 1106, 1042, 1015, 753.

5. Procedure for large scale experiment:

A 50 mL oven-dried Schlenk tube equipped with a magnetic stirring bar was subjected to three cycles of vacuum/argon backfill, and charged with aryne precursor 2-(trimethylsilyl)phenyl trifluoromethanesulfonate **2a** (1.22 mL, 5.0 mmol, 1.0 equiv.), TEMPO (781.3 mg, 5.0 mmol, 1.0 equiv.), CsF (2.28 g, 15 mmol, 3.0 equiv.) and methyl acrylate (2.15 mL, 25 mmol, 5 equiv.) which were dissolved in dry MeCN (25 mL, 0.20 M). The reaction mixture was stirred at room temperature for 24 h under argon atmosphere. Then the reaction mixture was filtered through silica pad and washed with diethyl ether. The solvent and excess of alkene were removed in a rotary evaporator under reduced pressure and the residue was subjected to flash column chromatography over silica gel (2% diethyl ether: pentane) to give the corresponding product **4a** (546 mg, 1.15 mmol, 46%).

6.Failed substrates:

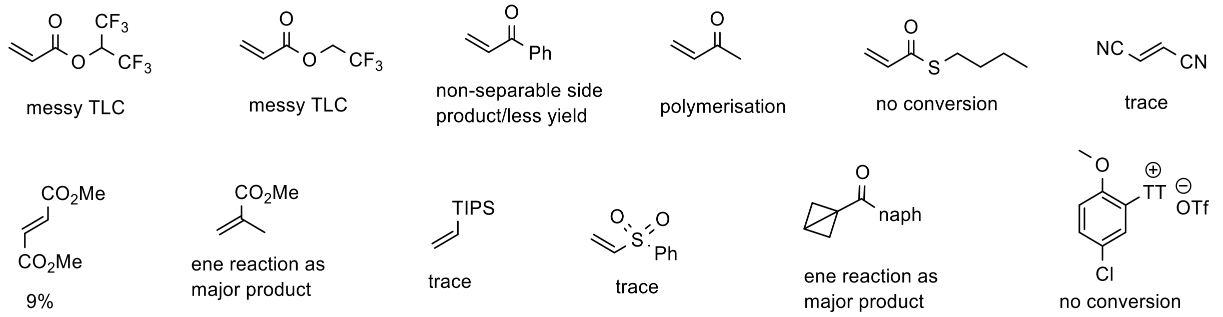

7.Crystallographic data:

X-Ray diffraction: Data sets for compounds **4a** and **4i** were collected with a Bruker D8 Venture Photon III Diffractometer. Programs used: data collection: *APEX4* Version 2021.4-0 [5] (Bruker AXS Inc., **2021**); cell refinement: *SAINT* Version 8.40B (Bruker AXS Inc., **2021**); data reduction: *SAINT* Version 8.40B (Bruker AXS Inc., **2021**); absorption correction, *SADABS* Version 2016/2 (Bruker AXS Inc., **2021**); structure solution *SHELXT*-Version 2018-3 [6] (Sheldrick, G. M. *Acta Cryst.*, **2015**, *A71*, 3-8); structure refinement *SHELXL*-Version 2018-3 [7] (Sheldrick, G. M. *Acta Cryst.*, **2015**, *C71* (1), 3-8) and graphics, *XP* [8] (Version 5.1, Bruker AXS Inc., Madison, Wisconsin, USA, **1998**). *R*-values are given for observed reflections, and wR^2 values are given for all reflections.

X-ray crystal structure analysis of 4a: A colorless, plate-like specimen of $C_{28}H_{46}N_2O_4$, approximate dimensions 0.052 mm x 0.060 mm x 0.152 mm, was used for the X-ray crystallographic analysis. The X-ray intensity data were measured on a single crystal diffractometer Bruker D8 Venture Photon III system equipped with a micro focus tube Cu ImS (CuKα, λ = 1.54178 Å) and a MX mirror monochromator. A total of 1769 frames were collected. The total exposure time was 22.43 hours. The frames were integrated with the Bruker SAINT software package using a wide-frame algorithm. The integration of the data using a monoclinic unit cell yielded a total of 50846 reflections to a maximum θ angle of 66.64° (0.84 Å resolution), of which 4704 were independent (average redundancy 10.809, completeness = 99.7%, R_{int} = 7.65%, R_{sig} = 3.09%) and 3884 (82.57%) were greater than 2σ(F^2). The final cell constants of a = 11.4757(2) Å, b = 7.6251(2) Å, c = 30.9076(7) Å, β = 99.5570(10)°, volume = 2666.98(10) Å^3, are based upon the refinement of the XYZ-centroids of 9905 reflections above 20 σ(I) with 8.775° < 2θ < 133.0°. Data were corrected for absorption effects using the Multi-Scan method (SADABS). The ratio of minimum to maximum apparent transmission was 0.895. The calculated minimum and maximum transmission coefficients (based on crystal size) are 0.9120 and 0.9690. The structure was solved and refined using the Bruker SHELXTL Software Package, using the space group $P2_1/n$, with Z = 4 for the formula unit, $C_{28}H_{46}N_2O_4$. The final anisotropic full-matrix least-squares refinement on F^2 with 316 variables converged at R1 = 3.79%, for the observed data and wR2 = 8.93% for all data. The goodness-of-fit was 1.046. The largest peak in the final difference electron density synthesis was 0.256 $e^-/Å^3$ and the largest hole was -0.197 $e^-/Å^3$ with an RMS deviation of 0.041 $e^-/Å^3$. On the basis of the final model, the calculated density was 1.182 g/cm^3 and F(000), 1040 e^-. CCDC Nr.: 2365425.

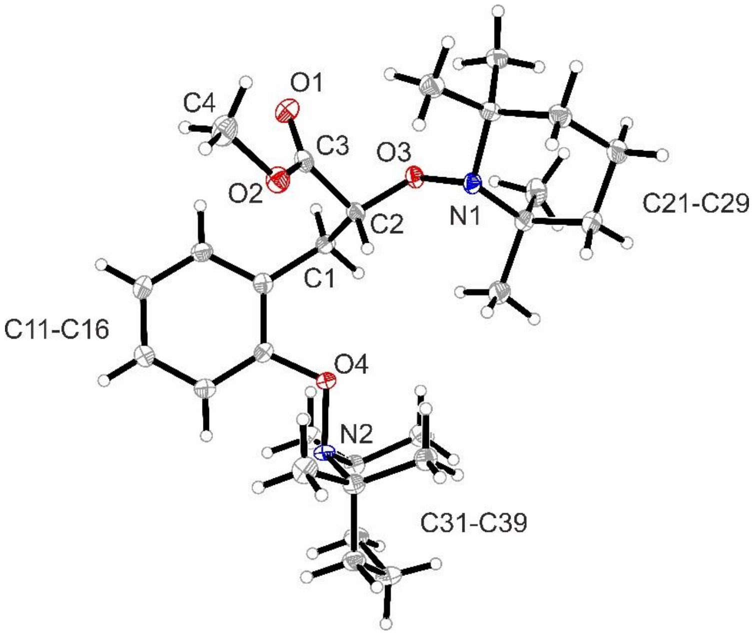

Figure S1: Crystal structure of compound **4a**.

Thermal ellipsoids are shown at 50% probability.

X-ray crystal structure analysis of 4i (stu10240): A colorless, plate-like specimen of $C_{26}H_{42}Cl_2N_2O_2$, approximate dimensions 0.063 mm x 0.093 mm x 0.246 mm, was used for the X-ray crystallographic analysis. The X-ray intensity data were measured on a single crystal diffractometer Bruker D8 Venture Photon III system equipped with a micro focus tube Cu ImS (CuKα, λ = 1.54178 Å) and a MX mirror monochromator. A total of 1609 frames were collected. The total exposure time was 19.76 hours. The frames were integrated with the Bruker SAINT software package using a wide-frame algorithm. The integration of the data using a monoclinic unit cell yielded a total of 52082 reflections to a maximum θ angle of 66.61° (0.84 Å resolution), of which 4587 were independent (average redundancy 11.354, completeness = 99.4%, R_{int} = 4.57%, R_{sig} = 2.12%) and 4213 (91.85%) were greater than $2\sigma(F^2)$. The final cell constants of a = 15.3322(6) Å, b = 7.6032(3) Å, c = 22.4351(8) Å, β = 91.006(2)°, volume = 2614.94(17) Å^3, are based upon the refinement of the XYZ-centroids of 9162 reflections above 20 σ(I) with 5.765° < 2θ < 133.0°. Data were corrected for absorption effects using the Multi-Scan method (SADABS). The ratio of minimum to maximum apparent transmission was 0.831. The calculated minimum and maximum transmission coefficients (based on crystal size) are 0.5880 and 0.8630. The structure was solved and refined using the Bruker SHELXTL Software Package, using the space group $P2_1/c$, with Z = 4 for the formula unit, $C_{26}H_{42}Cl_2N_2O_2$. The final anisotropic full-matrix least-squares refinement on F^2 with 297 variables converged at R1 = 3.07%, for the observed data and wR2 = 7.77% for all data. The goodness-of-fit was 1.028. The largest peak in the final difference electron density synthesis was 0.274 e^-/Å^3 and the largest hole was -0.310 e^-/Å^3 with an RMS deviation of 0.039 e^-/Å^3. On the basis of the final model, the calculated density was 1.233 g/cm^3 and F(000), 1048 e^-. CCDC Nr.: 2365426.

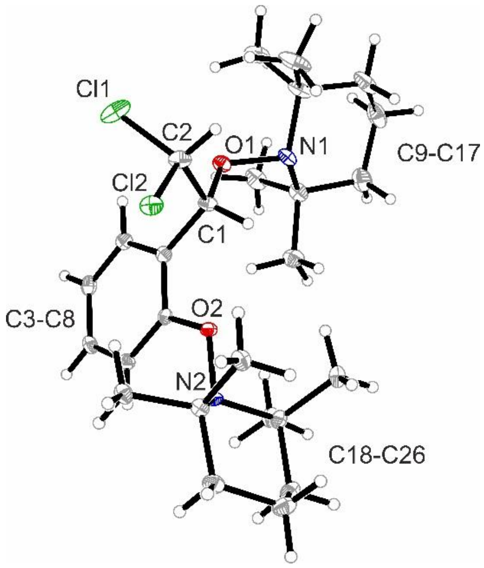

Figure S2: Crystal structure of compound **4i**.
Thermal ellipsoids are shown at 50% probability.

9. References:

1. C. Wan, Y. Guo, X. Chen, R. Gu, J. Shi & Y. Li, *Org. Lett.*, 2022, **24**, 7276-7281.

2. Chen & M. C. Willis, *Org. Lett.* 2015, **17**, 4786-4789.

3. B. S. Shaibu, R. K. Kawade, R.-S. Liu, *Org. Biomol. Chem.* 2012, **10**, 6834-6839.

5. A. Aybey & A.S. Demir, *Tetrahedron Lett*. 2008, **64**, 11256-11261.

5. Bruker AXS (**2021**) *APEX4 Version 2021.4-0*, *SAINT Version 8.40B* and *SADABS Bruker AXS area detector scaling and absorption correction Version 2016/2*, Bruker AXS Inc., Madison, Wisconsin, USA.

6. Sheldrick, G. M., *SHELXT – Integrated space-group and crystal-structure determination, Acta Cryst.,* **2015**, *A71*, 3-8.

7. Sheldrick, G.M., *Crystal structure refinement with SHELXL, Acta Cryst.*, **2015**, *C71 (1)*, 3-8.

8. Bruker AXS (**1998**) *XP – Interactive molecular graphics, Version 5.1*, Bruker AXS Inc., Madison, Wisconsin, USA.

9. NMR spectra of the products:

^{1}H NMR of 4a

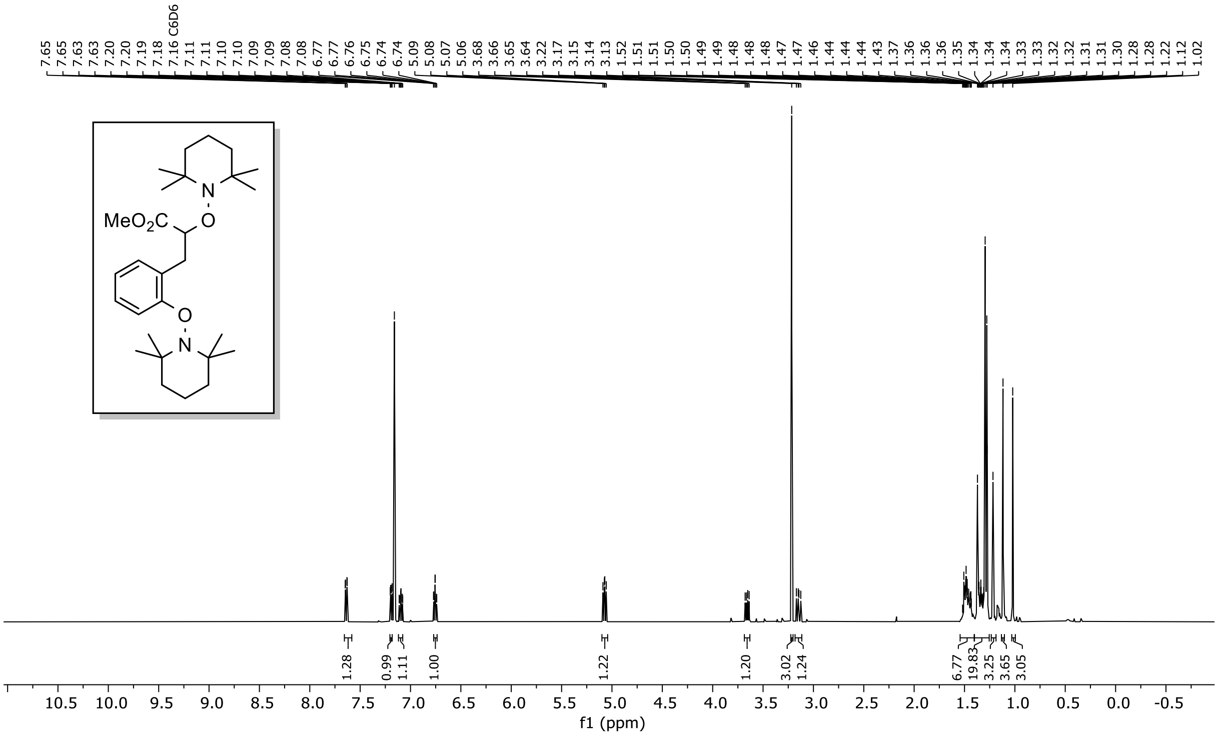

^{13}C NMR of 4a

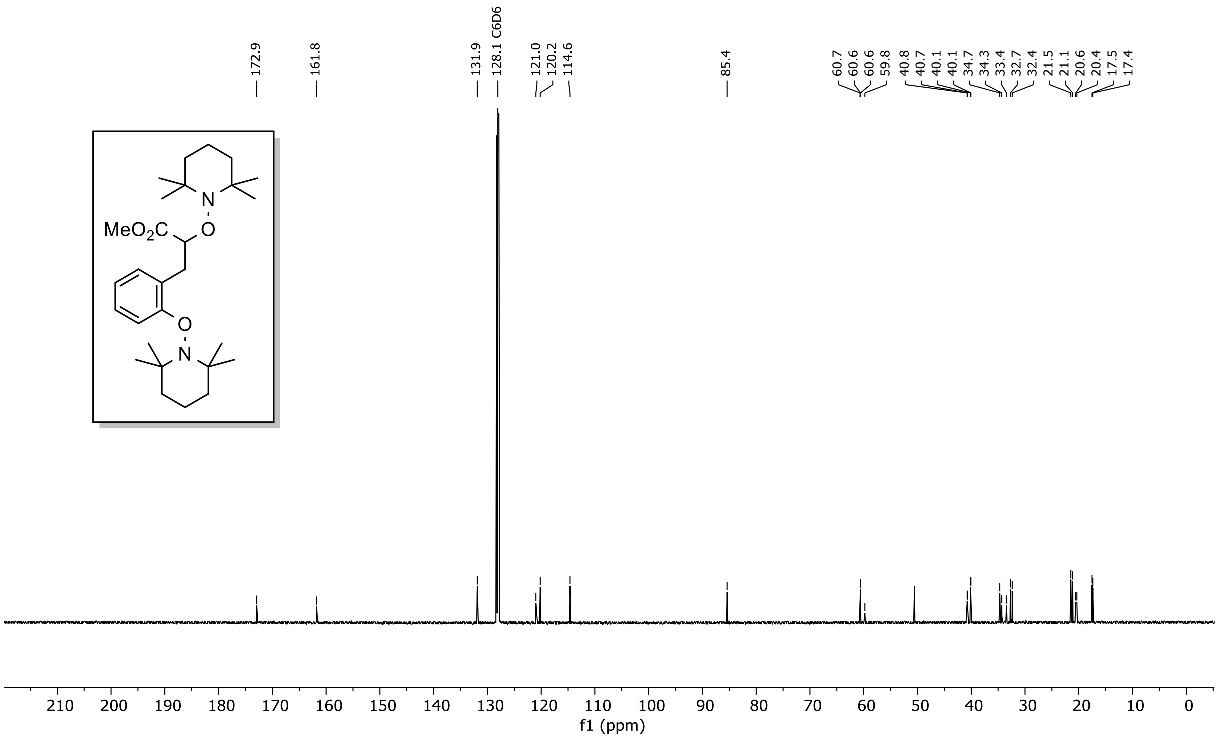

^{1}H NMR of 4b

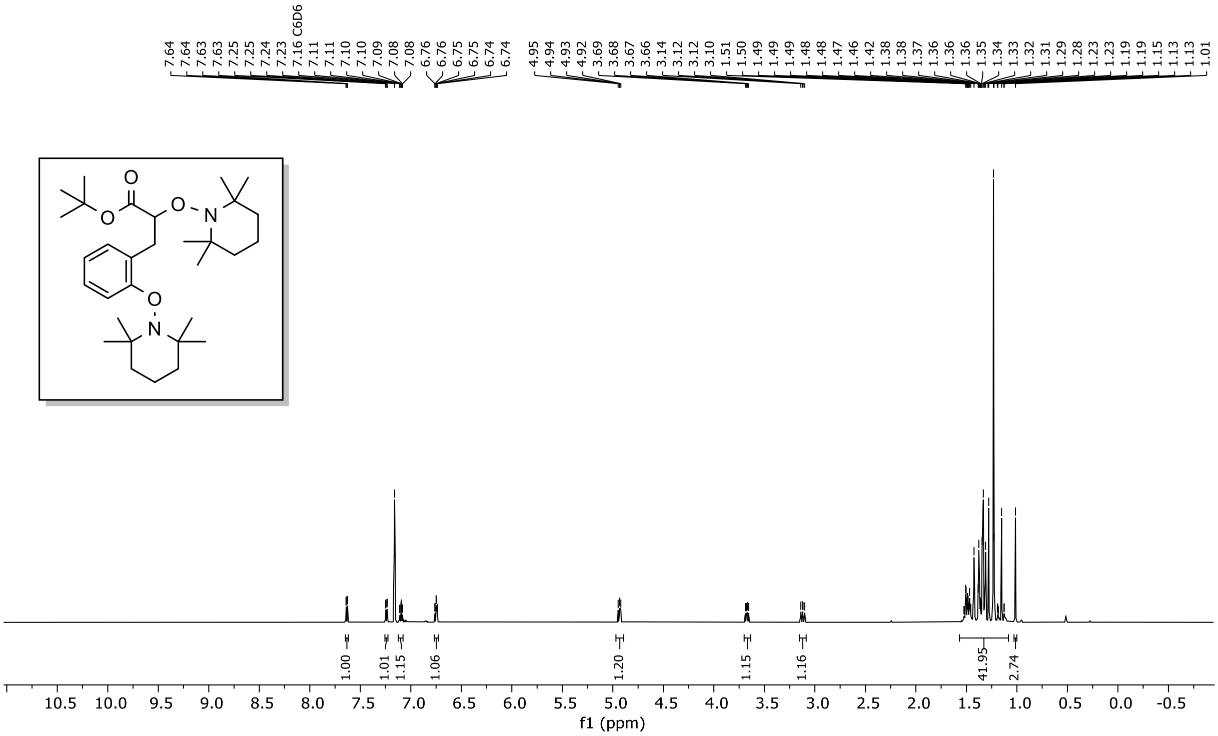

^{13}C NMR of 4b

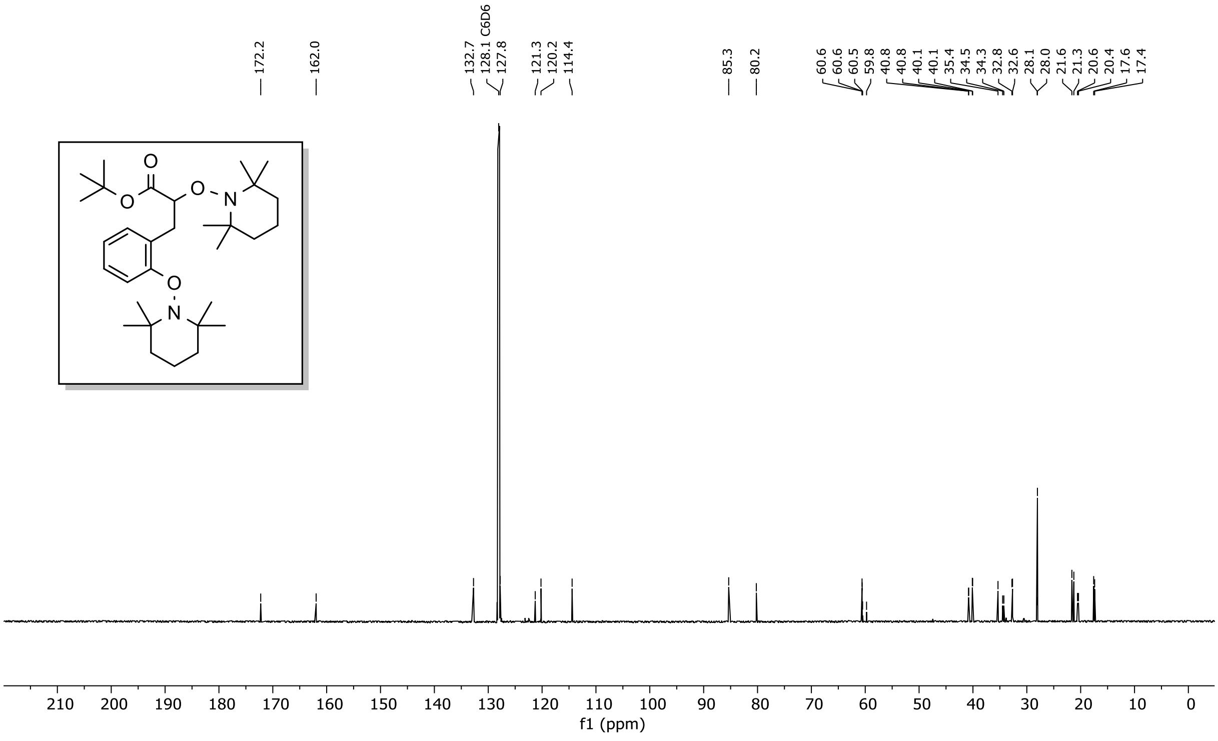

^{1}H NMR of 4c

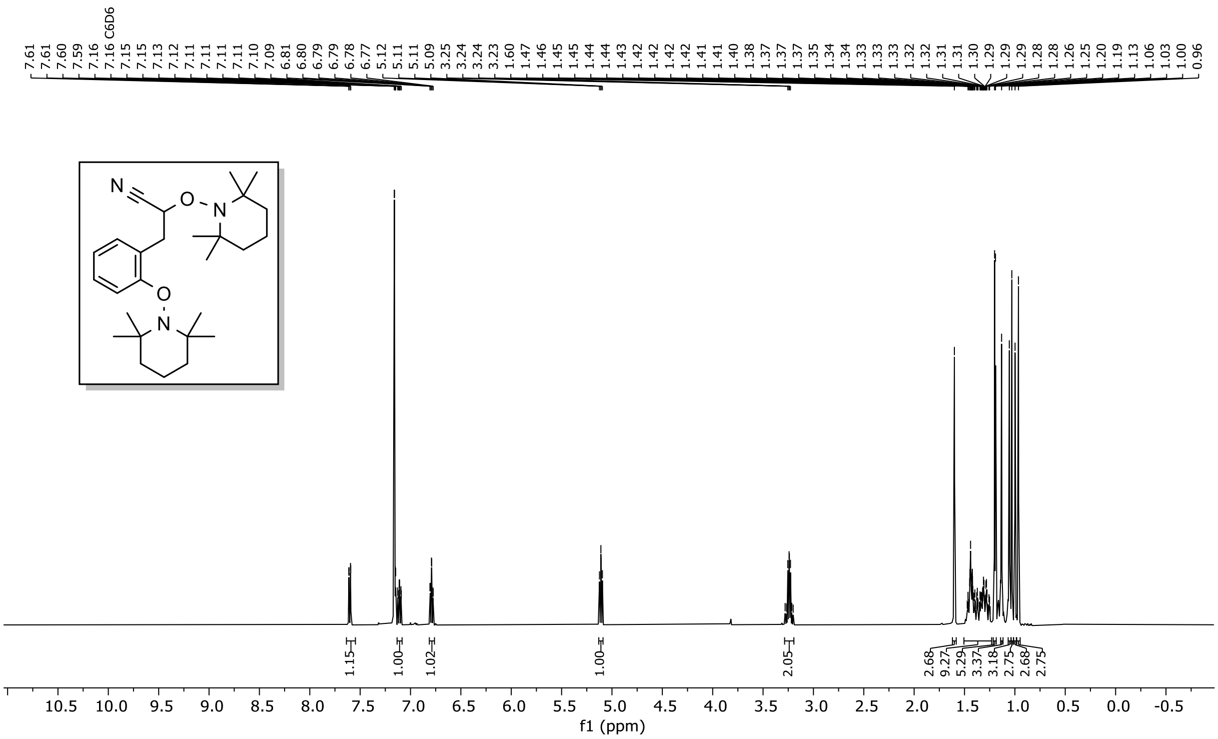

^{13}C NMR of 4c

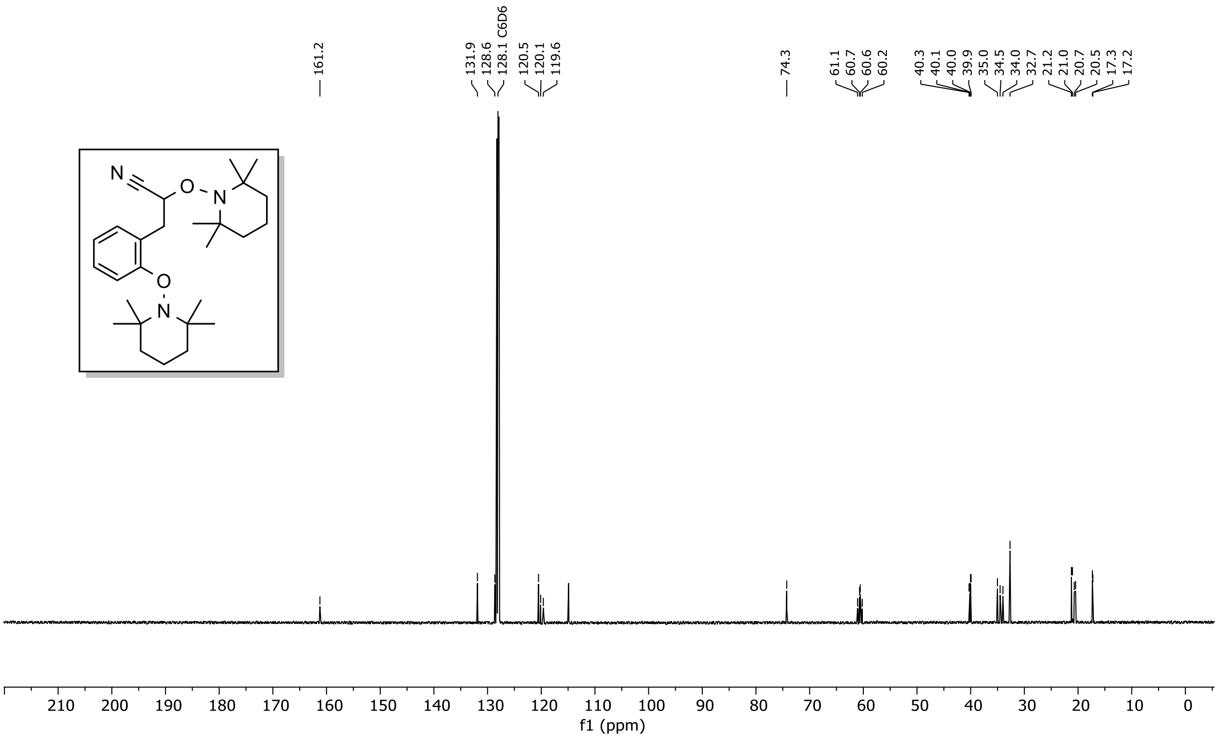

^{1}H NMR of 4d

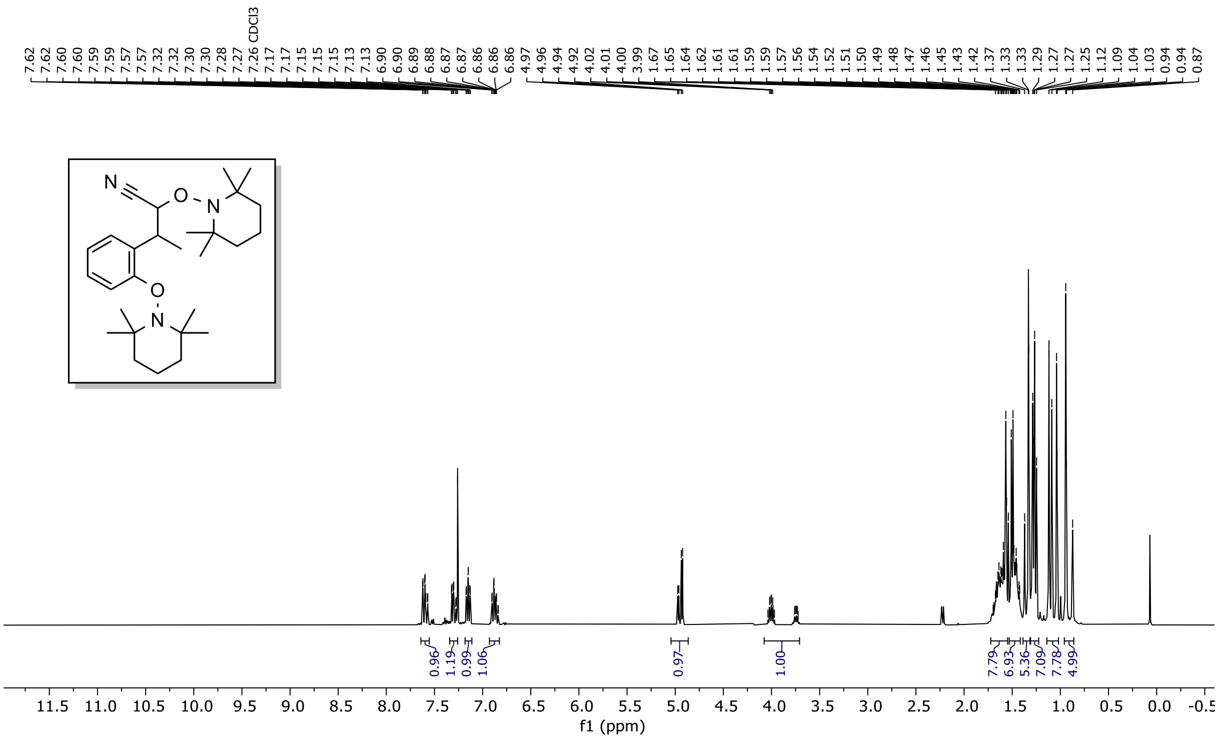

^{13}C NMR of 4d

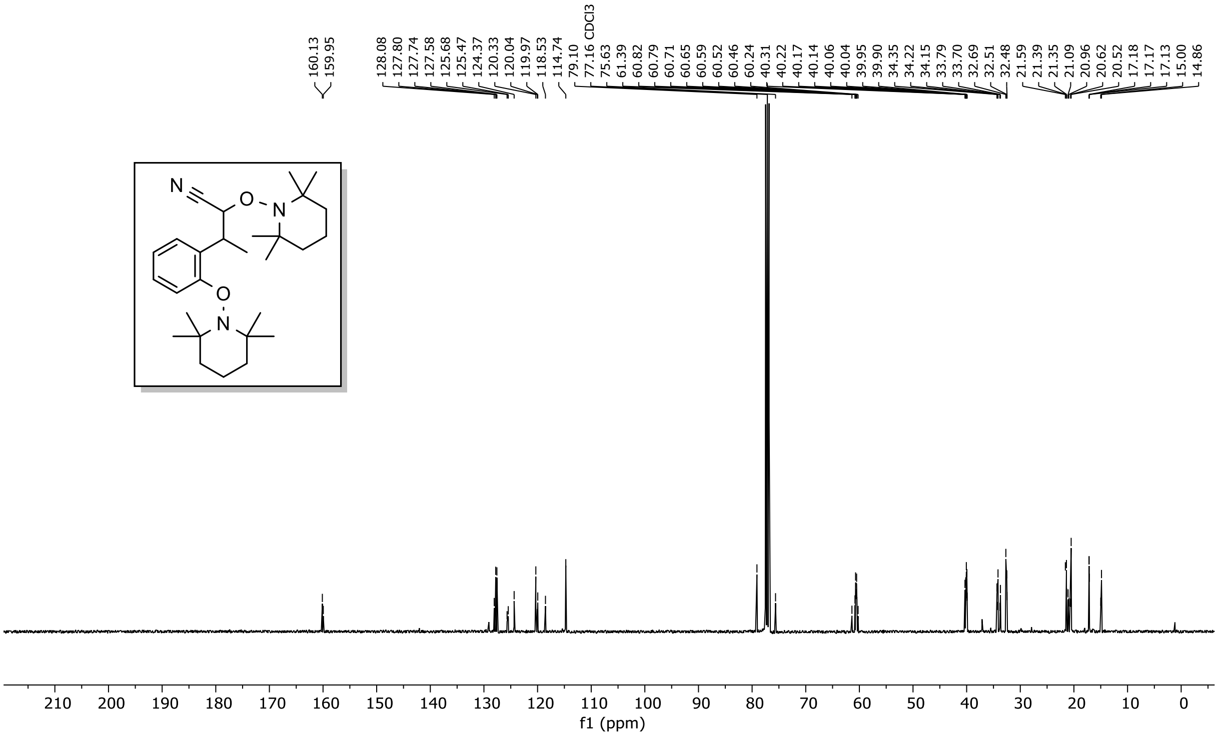

^{1}H NMR of 4e

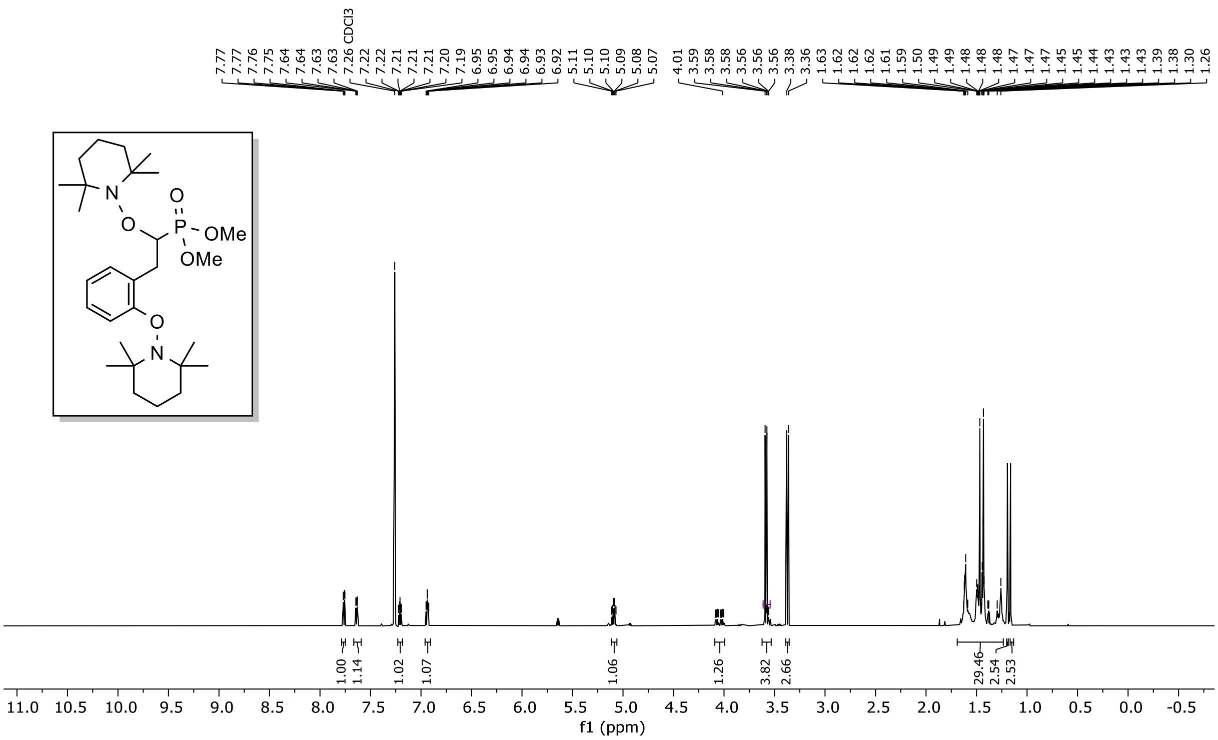

^{13}C NMR of 4e

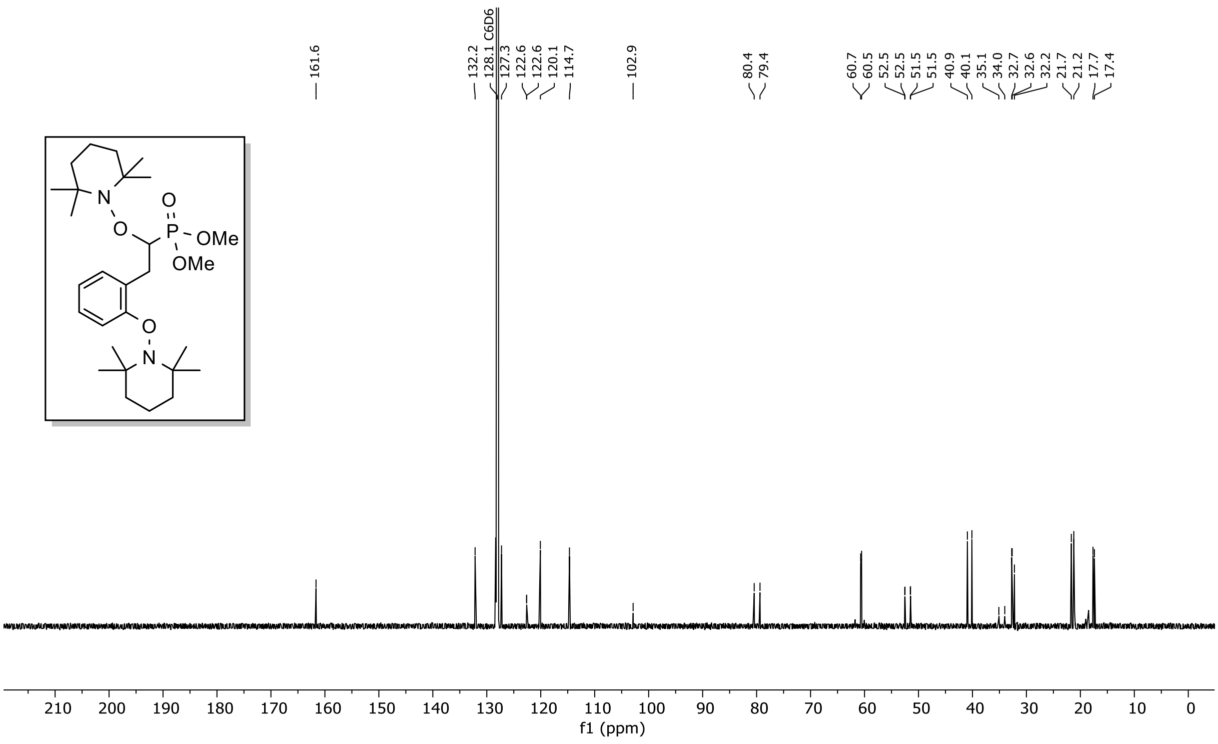

[13]P NMR of 4e

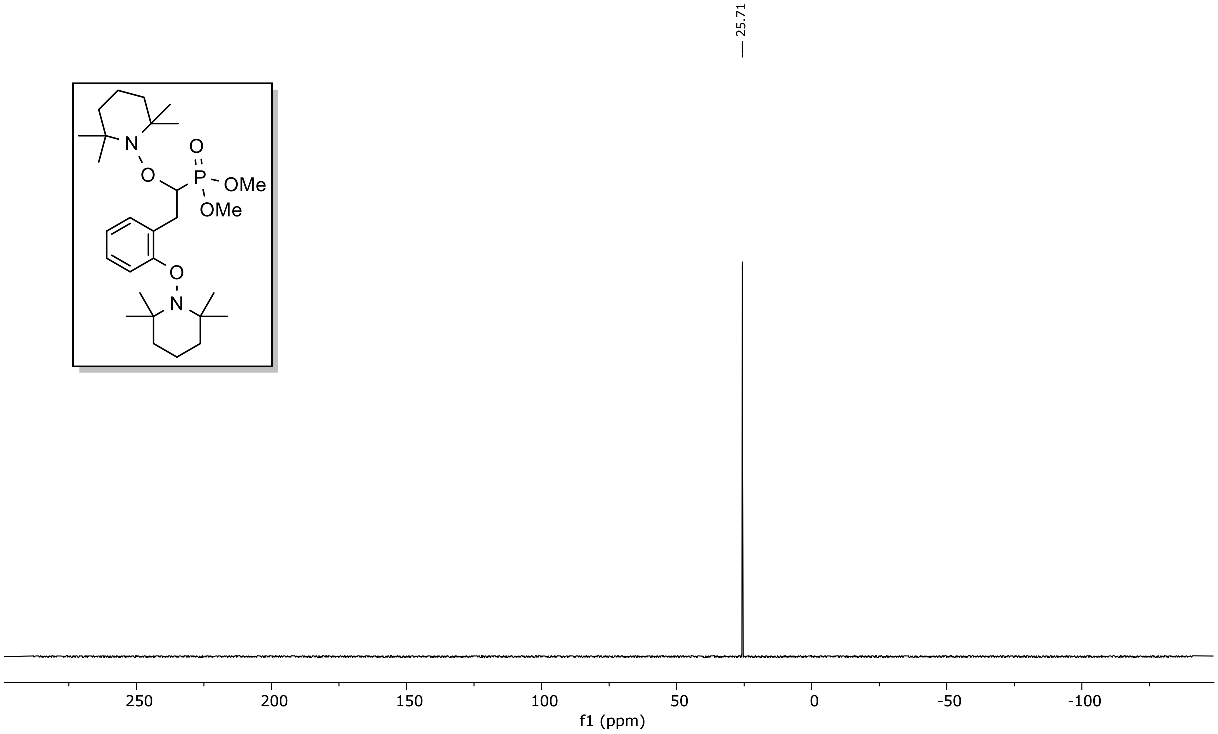

^{1}H NMR of 4f

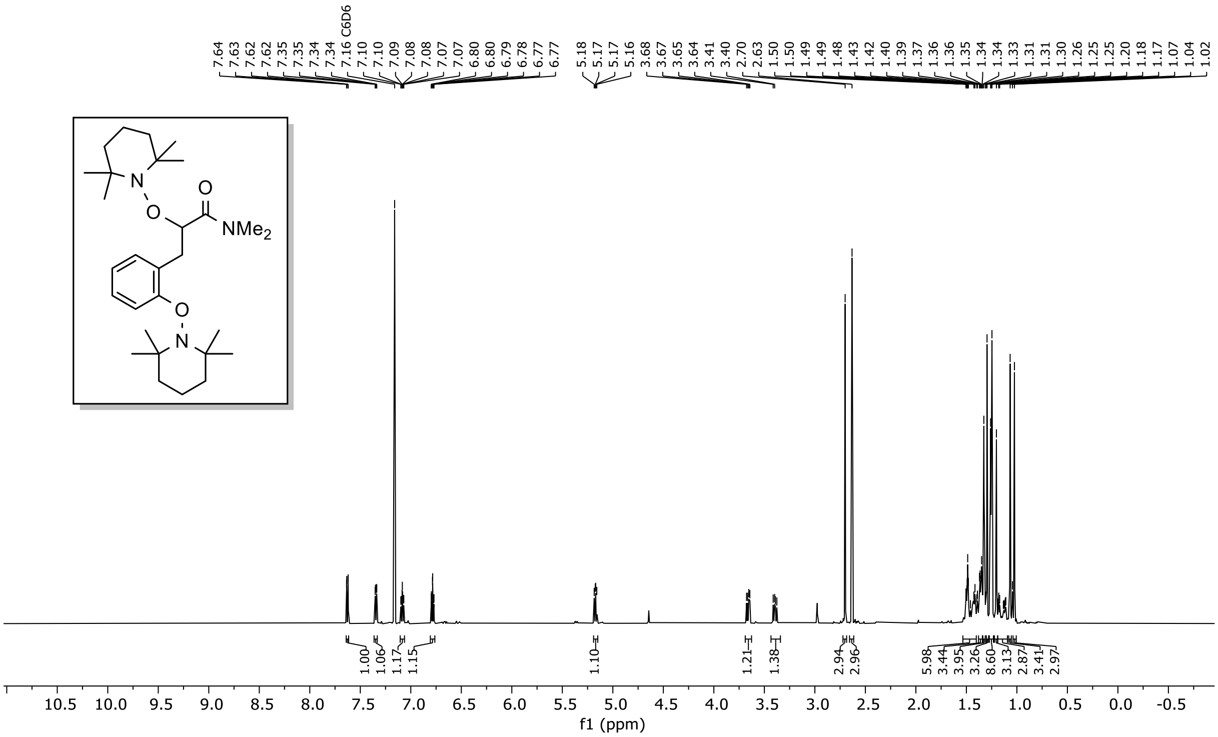

^{13}C NMR of 4f

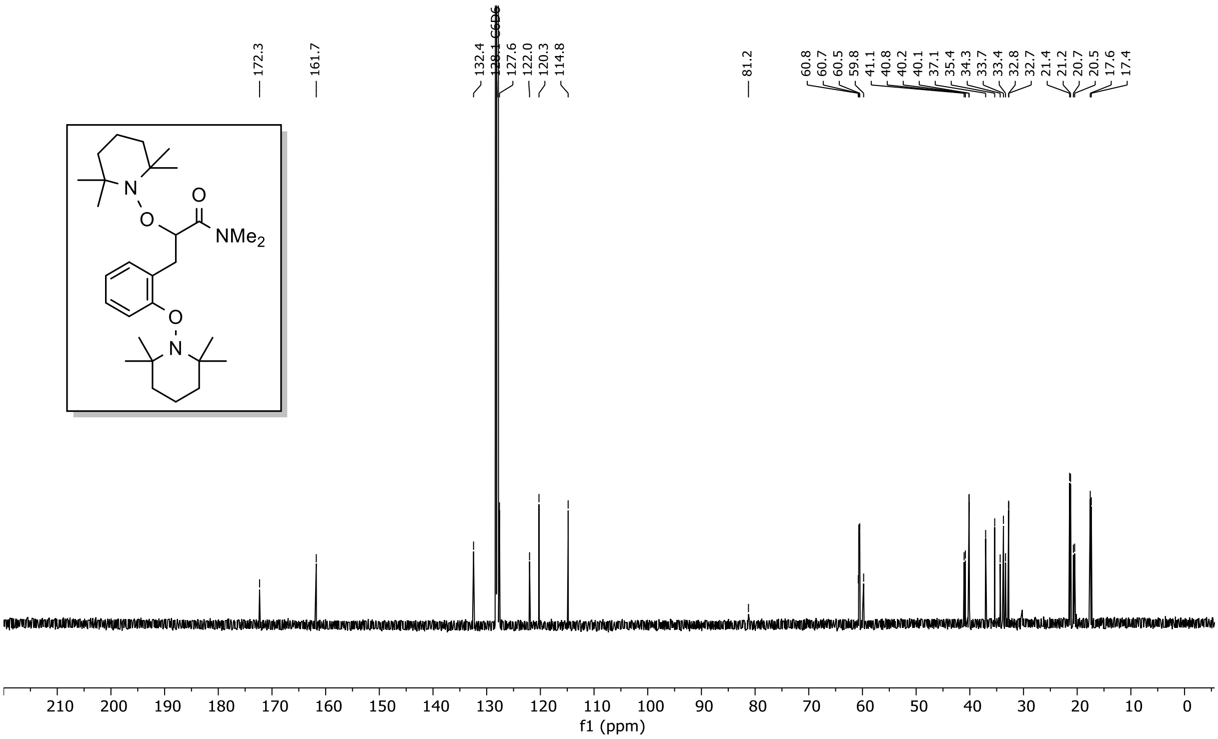

^{1}H NMR of 4g

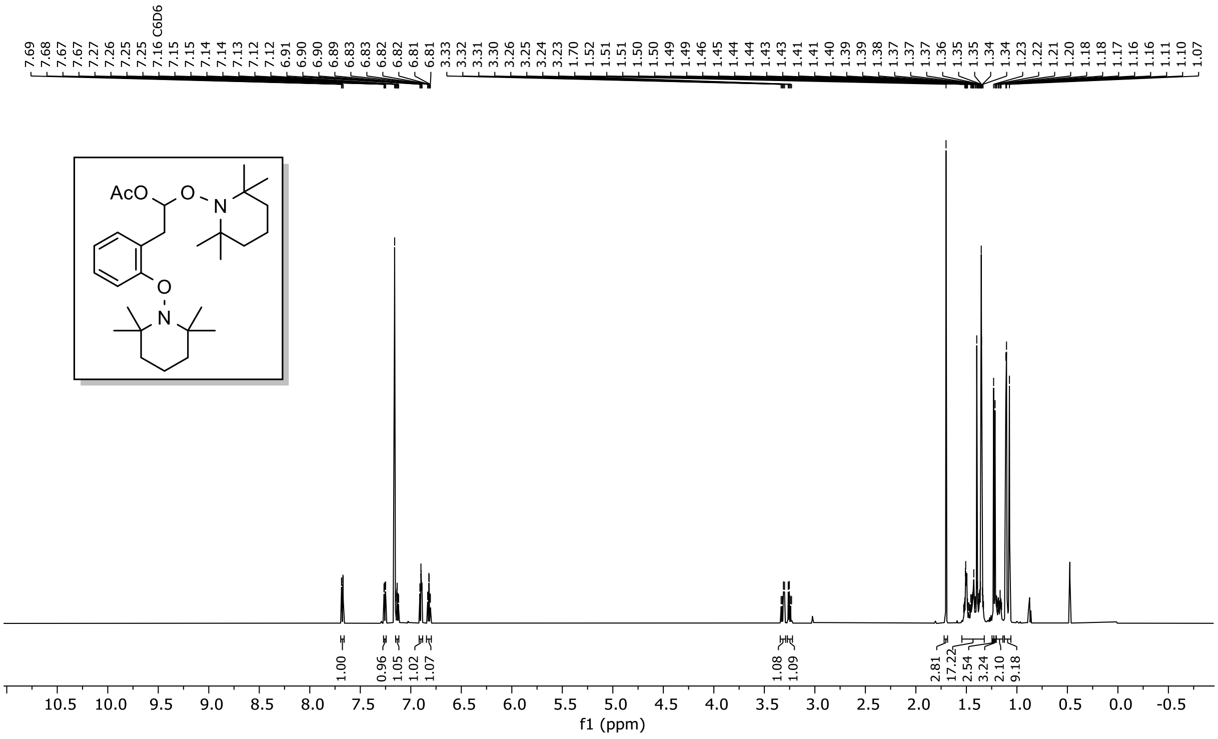

^{13}C NMR of 4g

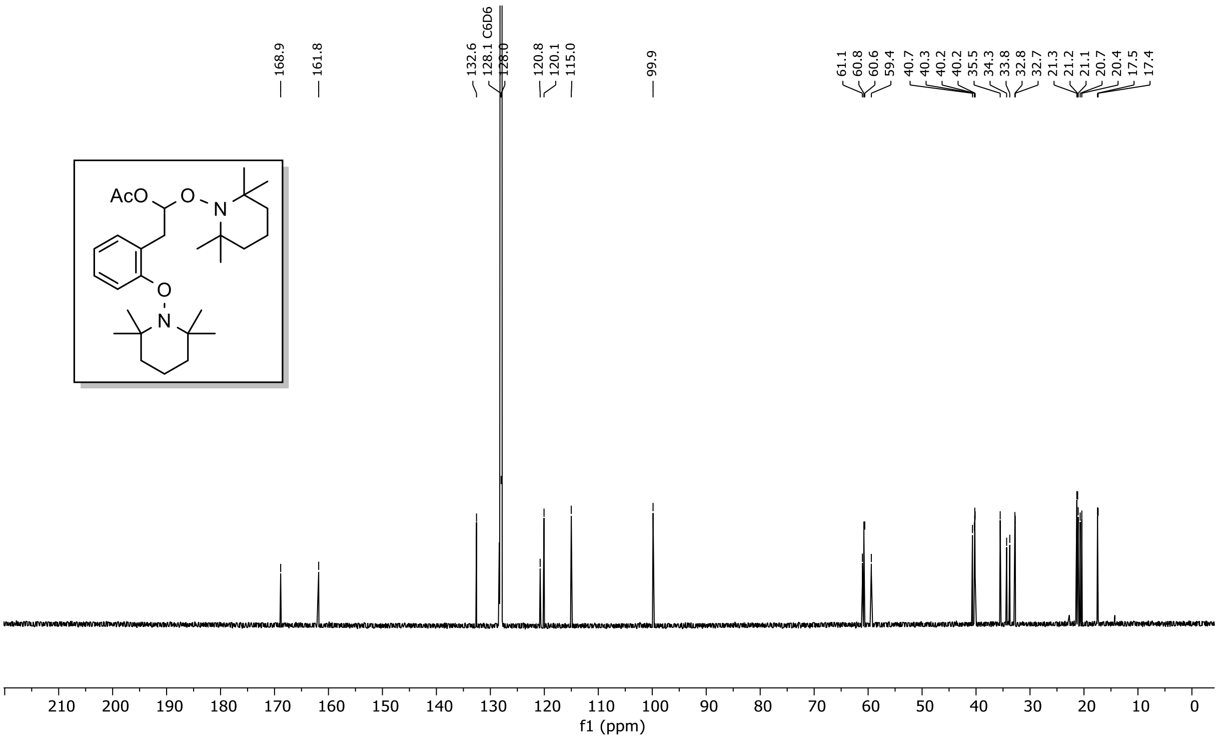

^{1}H NMR of 4h

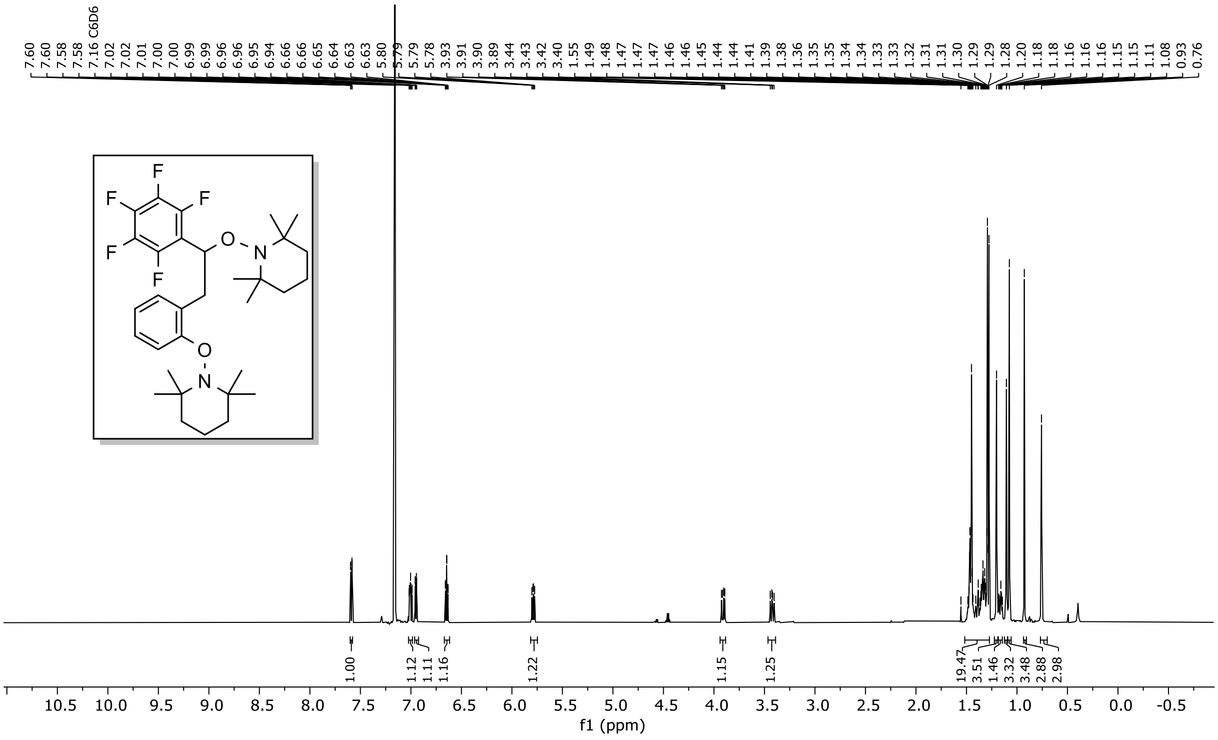

^{13}C NMR of 4h

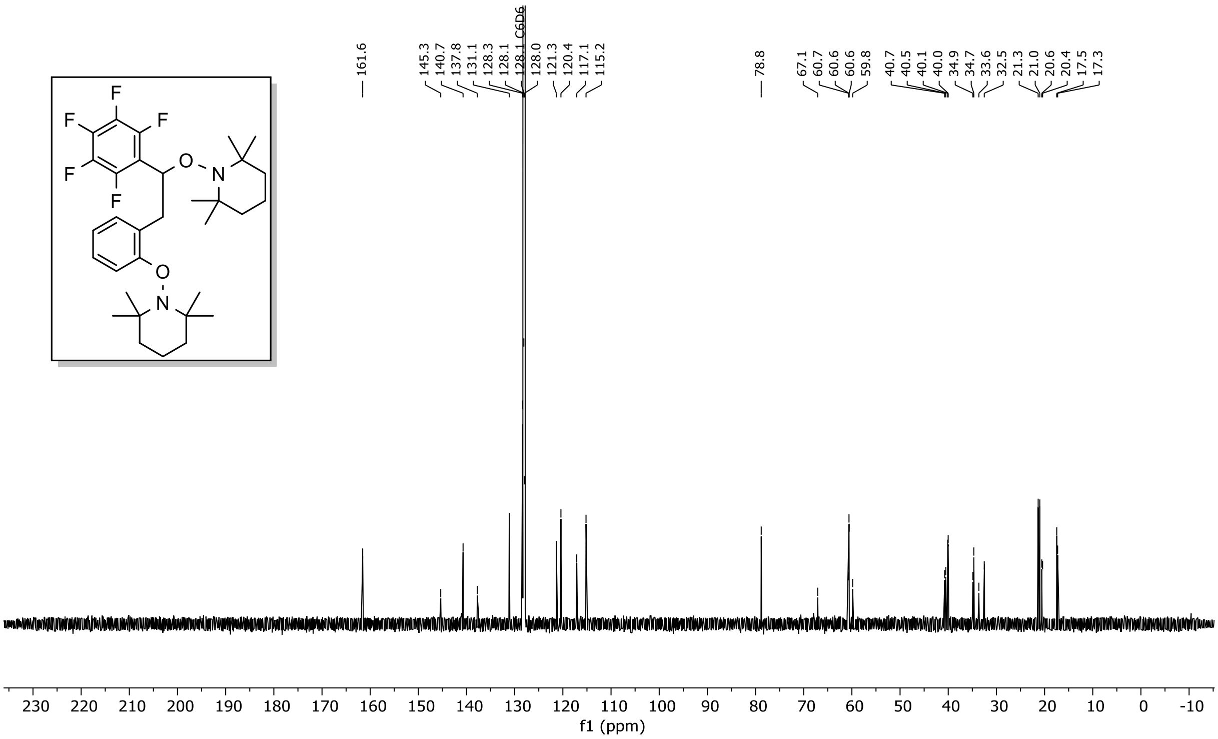

^{19}F NMR of 4h

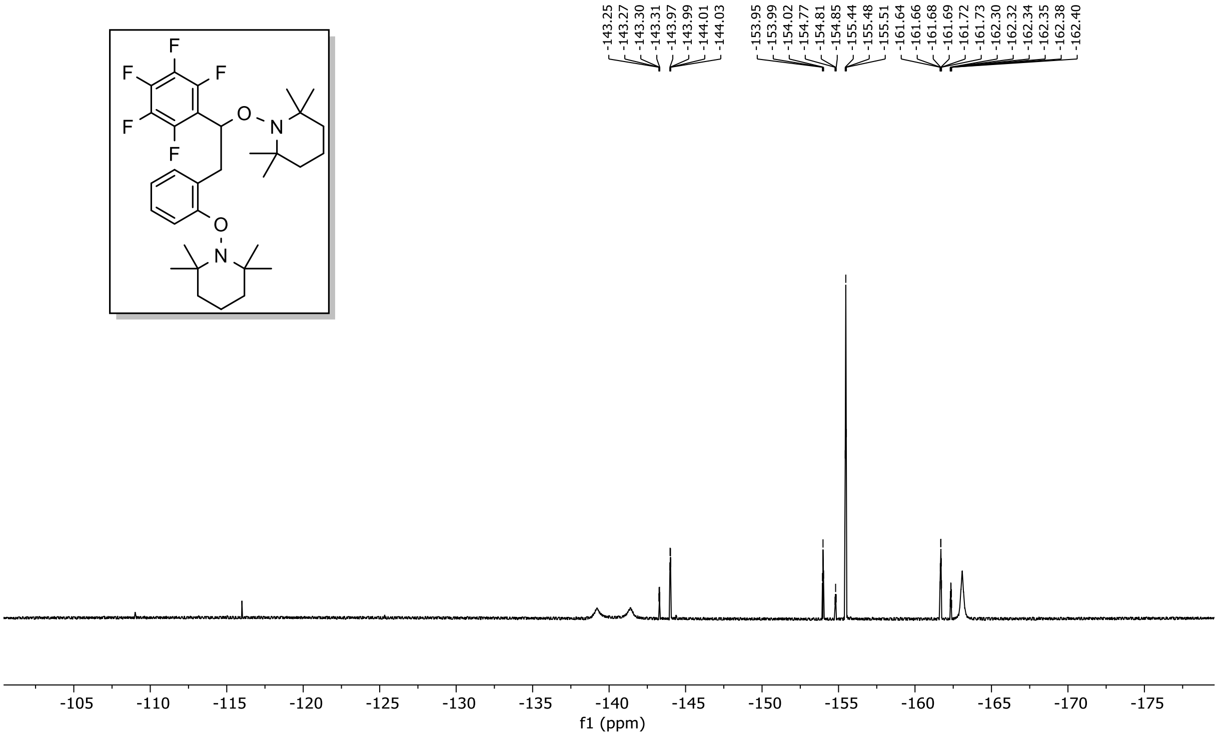

^{1}H NMR of 4i

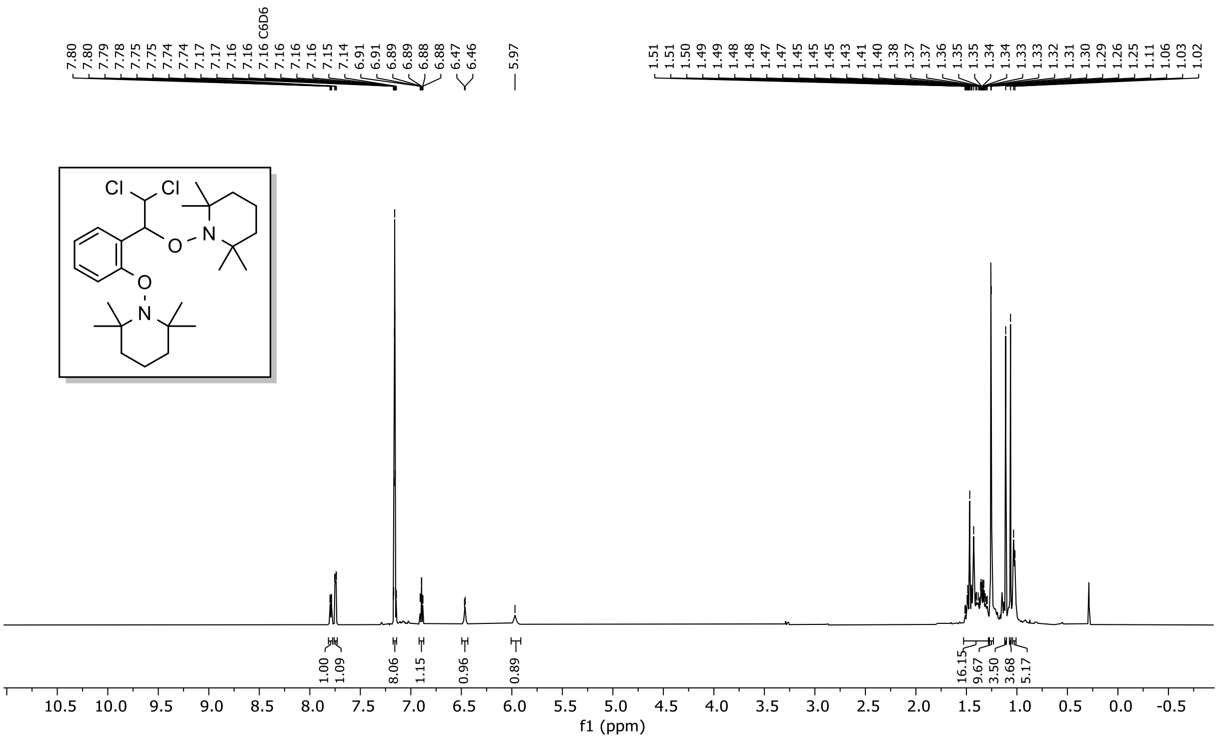

^{13}C NMR of 4i

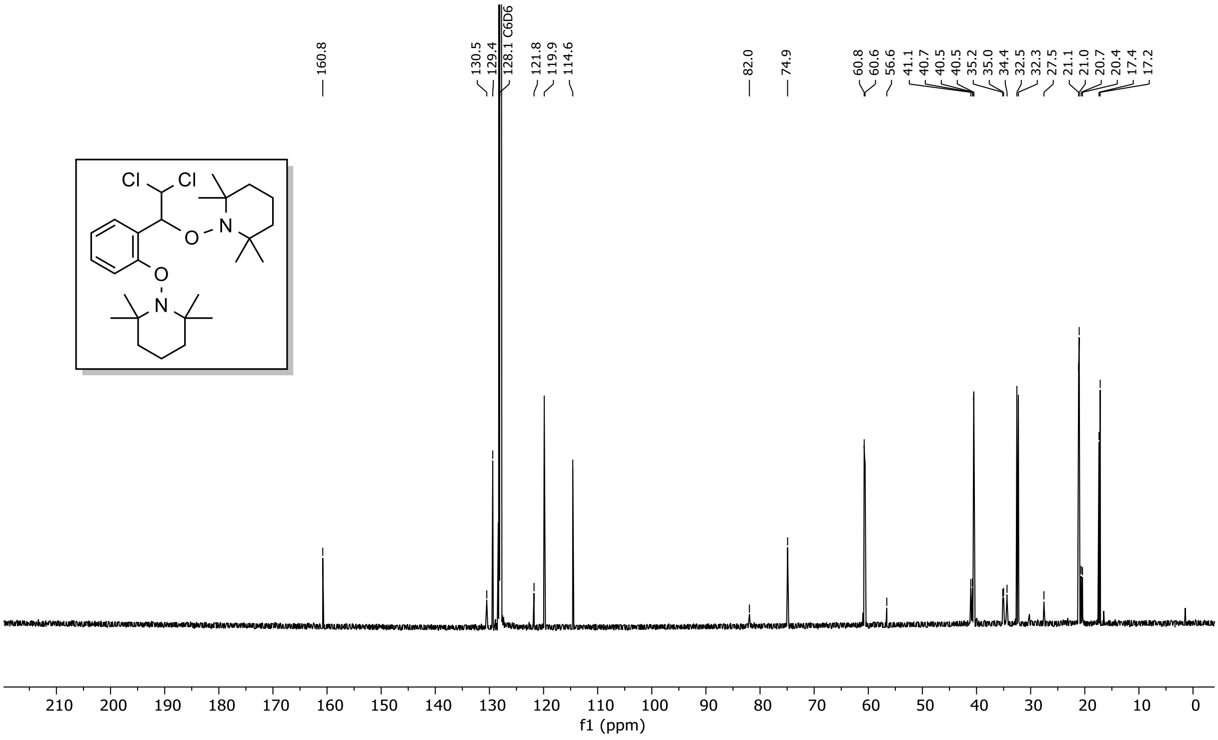

^{1}H NMR of 6a

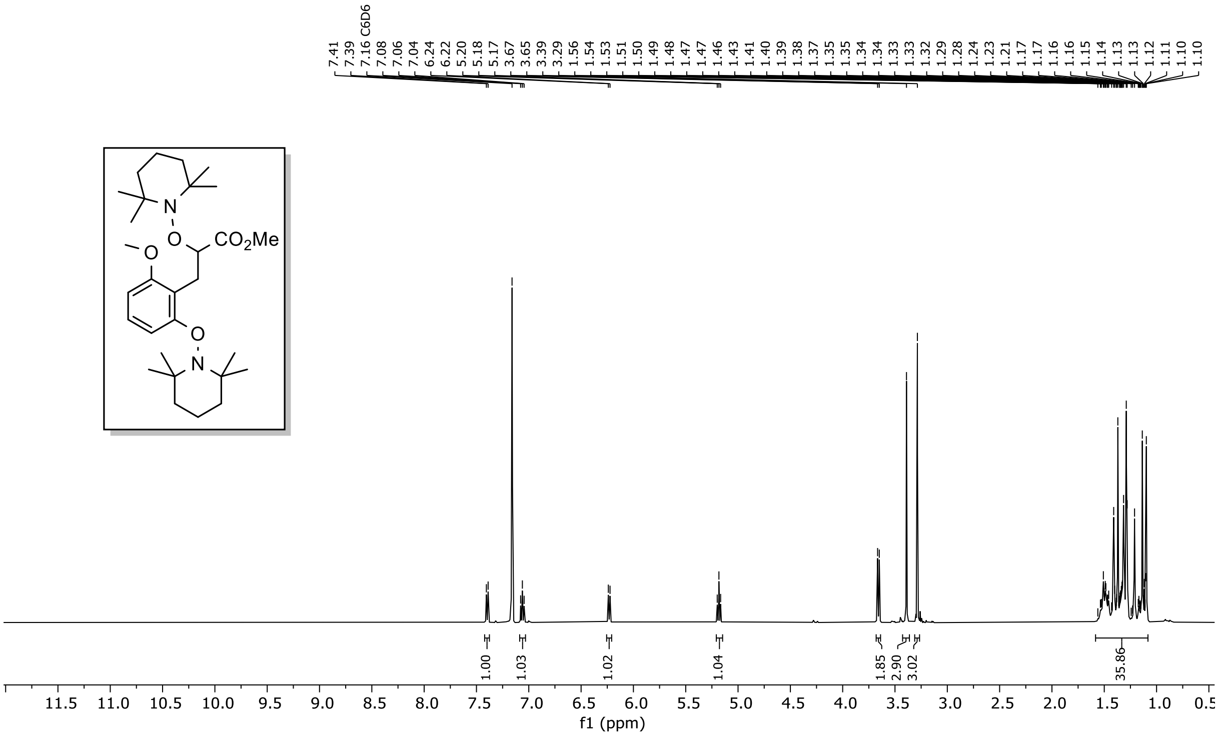

^{13}C NMR of 6a

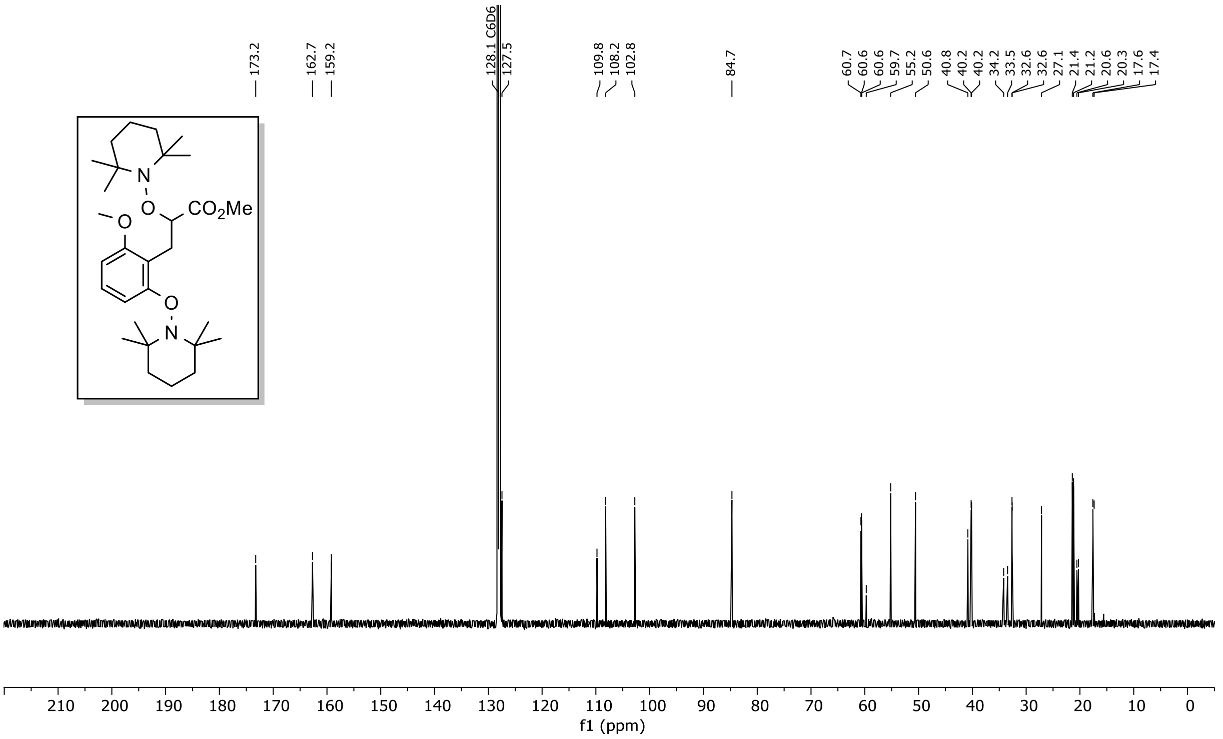

^{1}H NMR of 6b

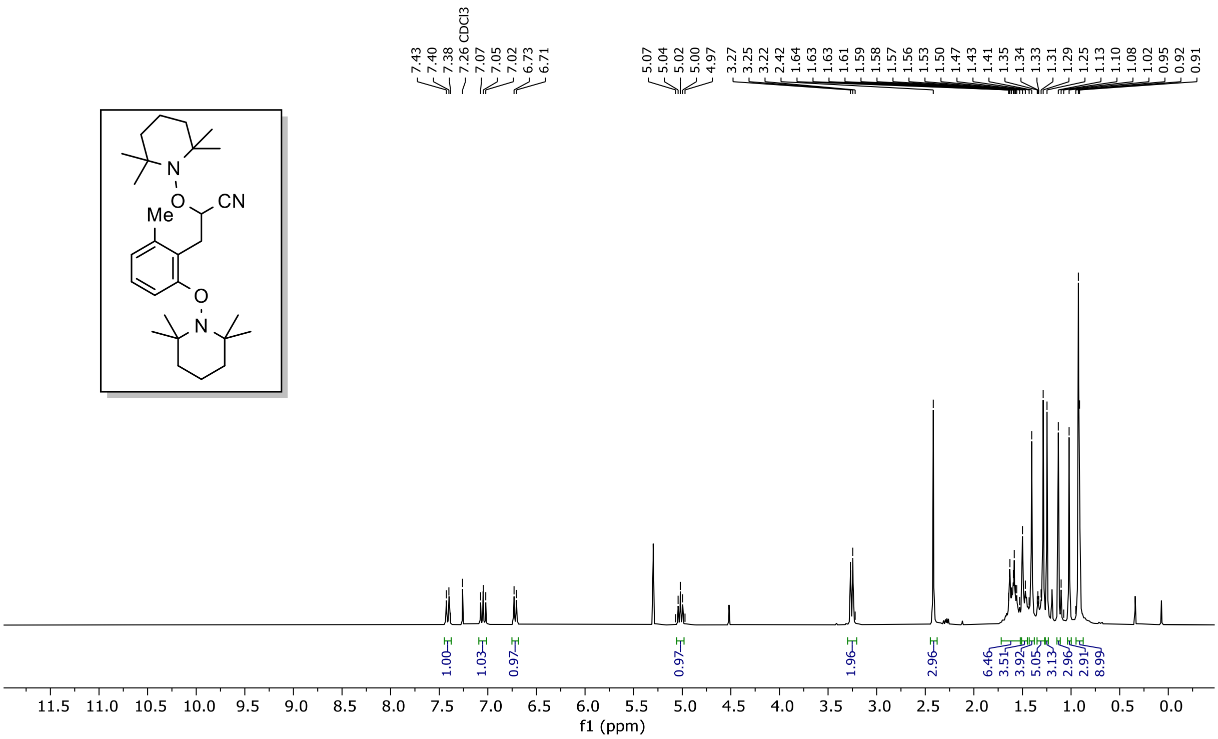

^{13}C NMR of 6b

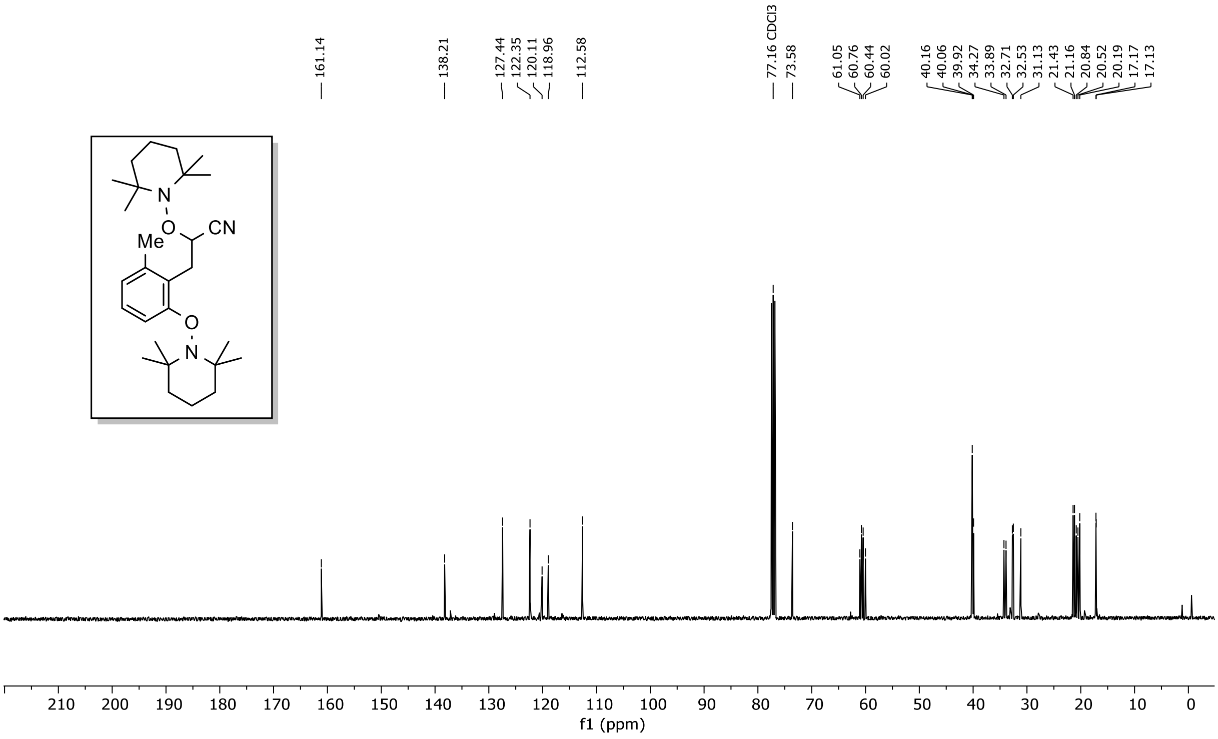

^{1}H NMR of 6c

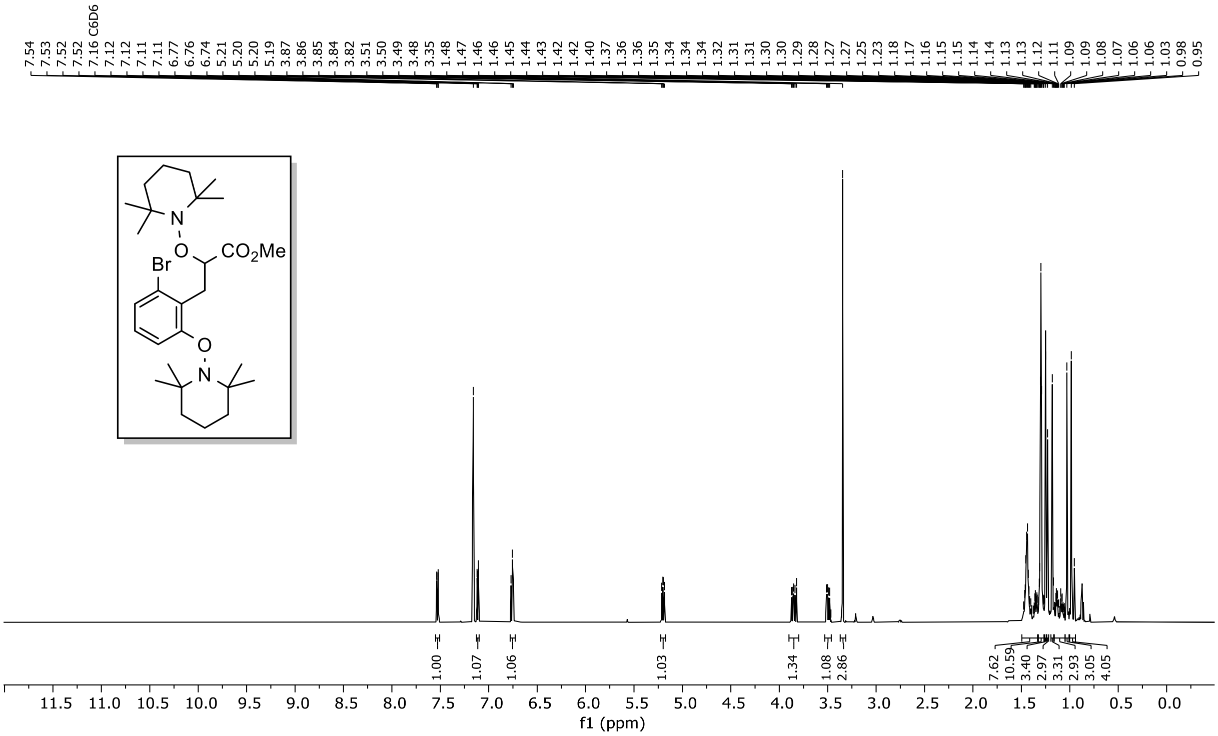

^{13}C NMR of 6c

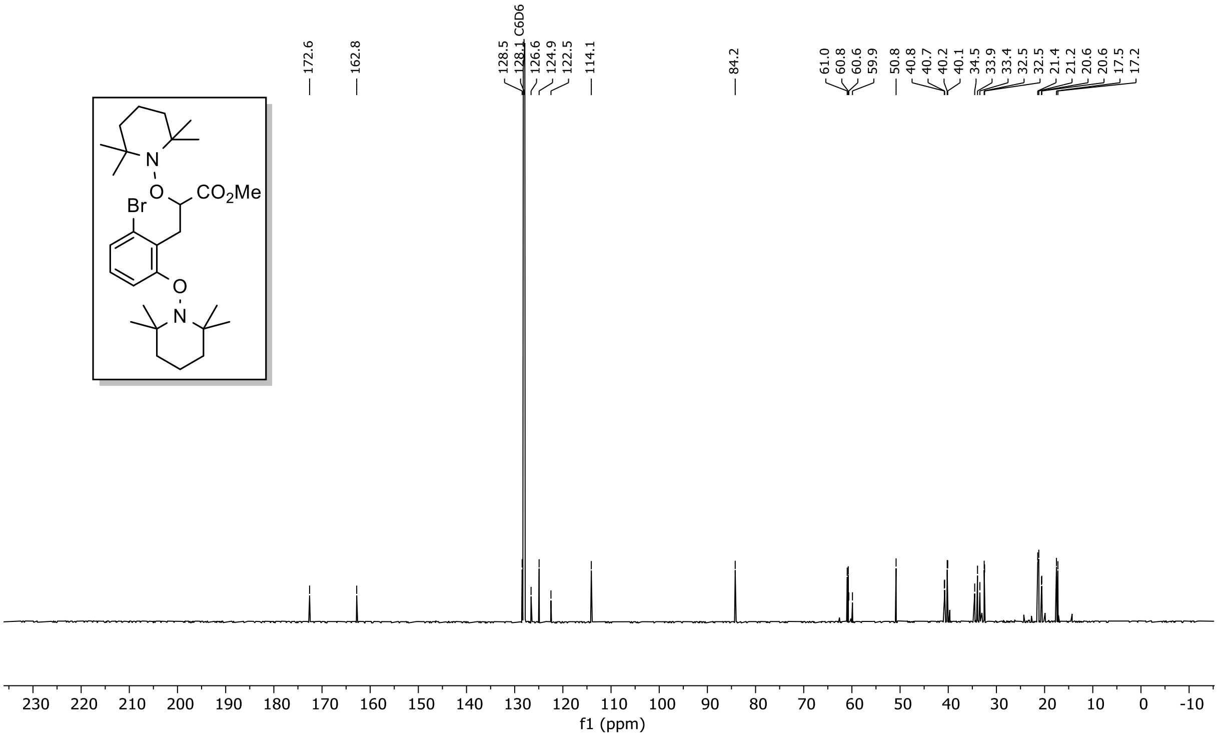

^{1}H NMR of 6d

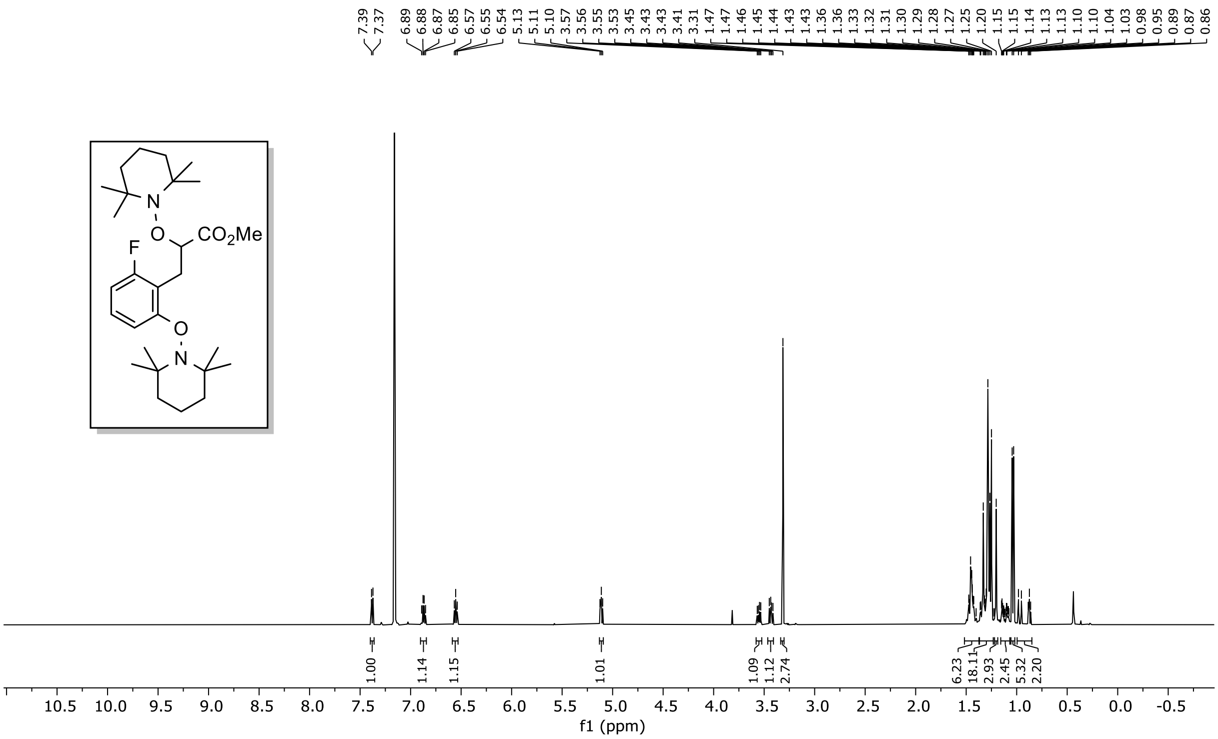

^{13}C NMR of 6d

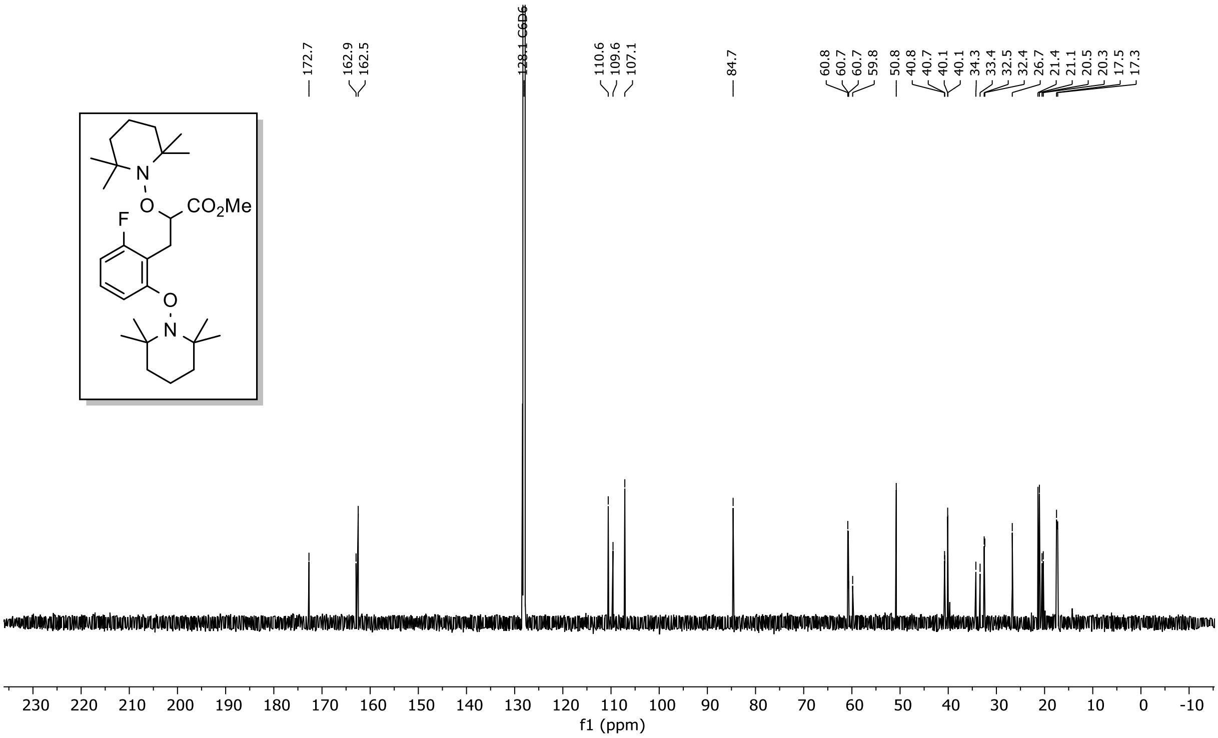

^{19}F NMR of 6d

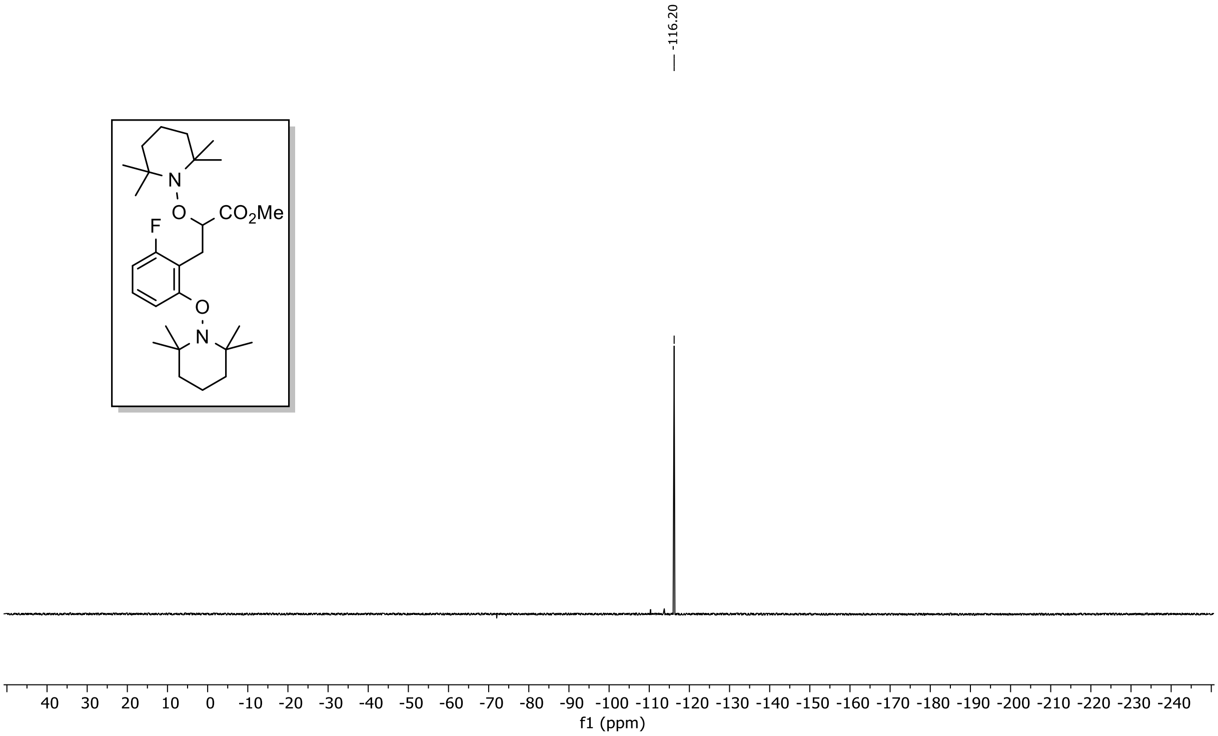

^{1}H NMR of 6e

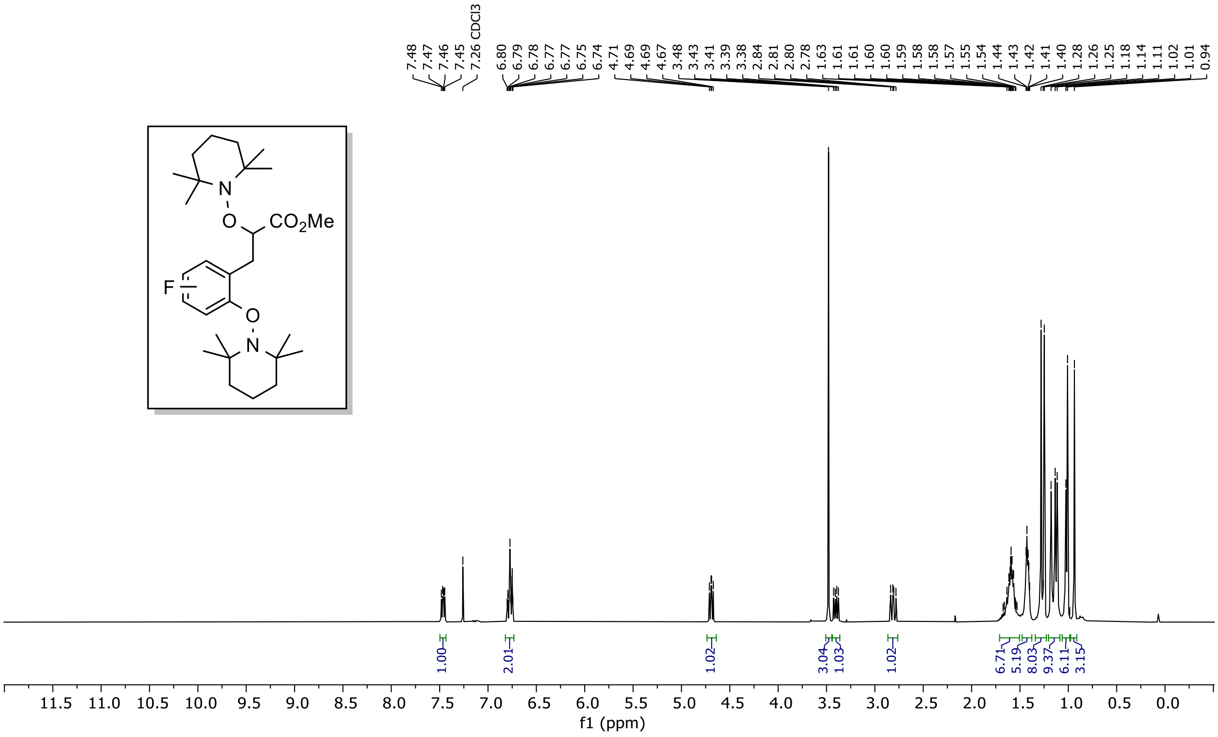

^{13}C NMR of 6e

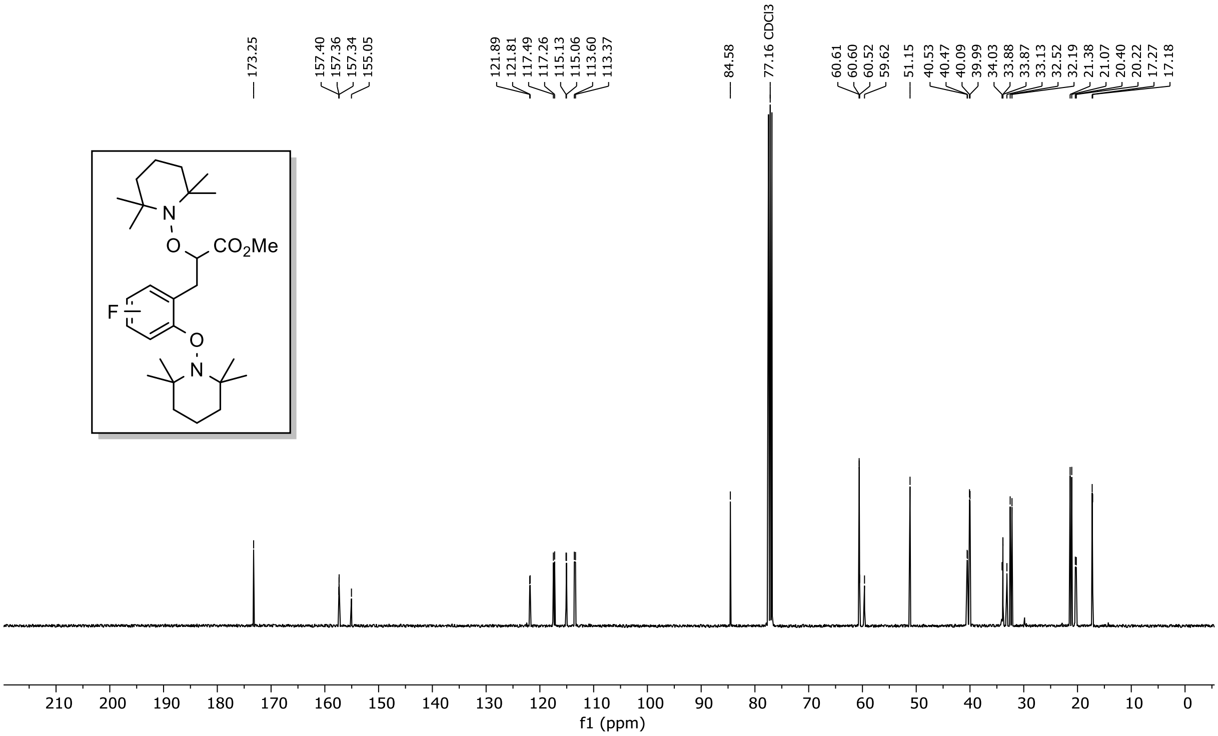

^{19}F NMR of 6e

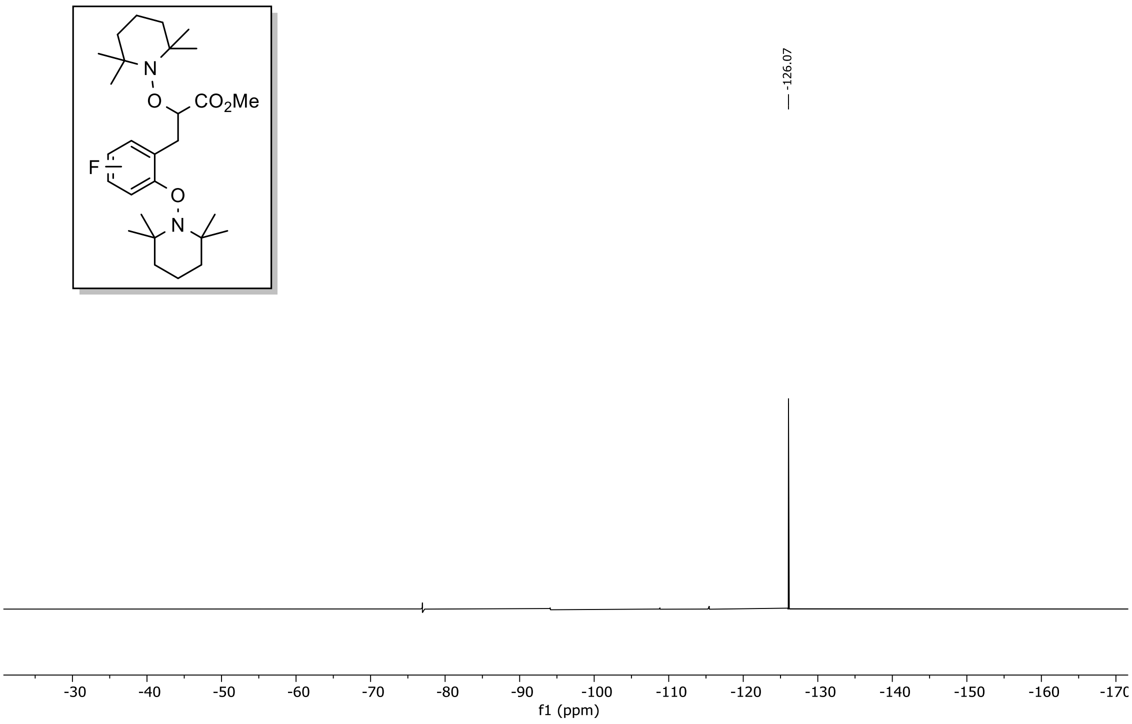

^{1}H NMR of 6f

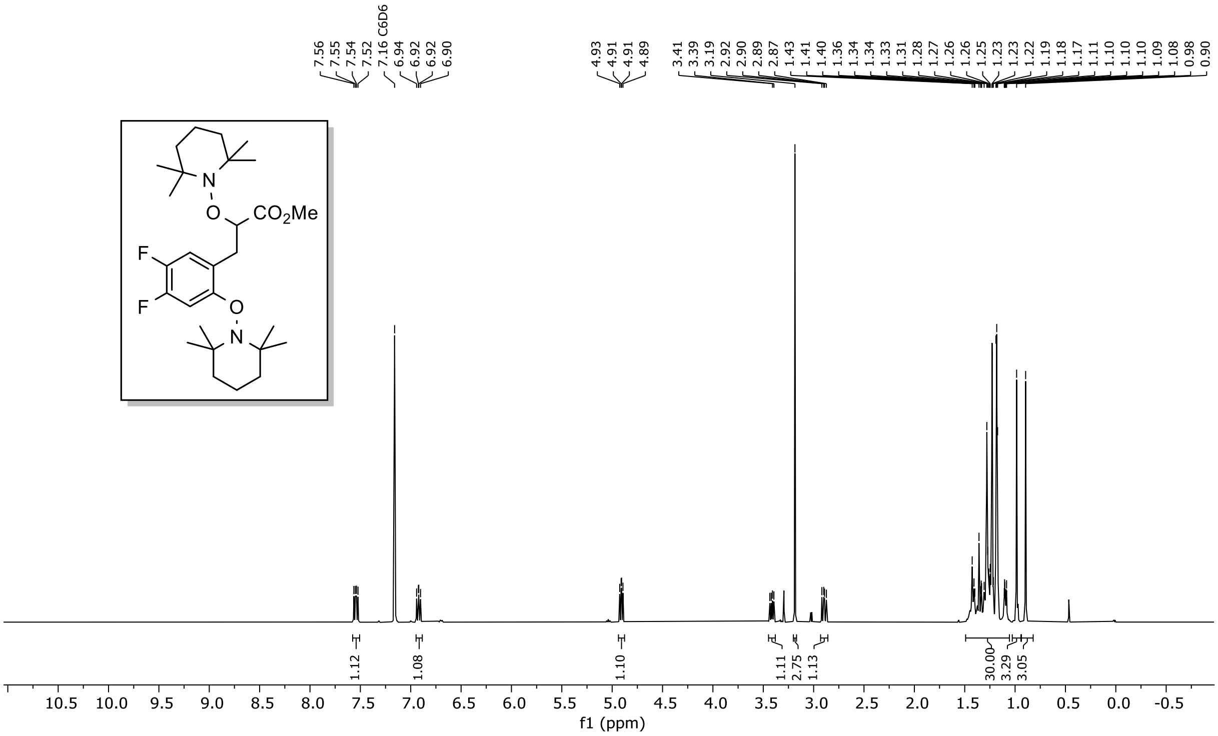

^{13}C NMR of 6f

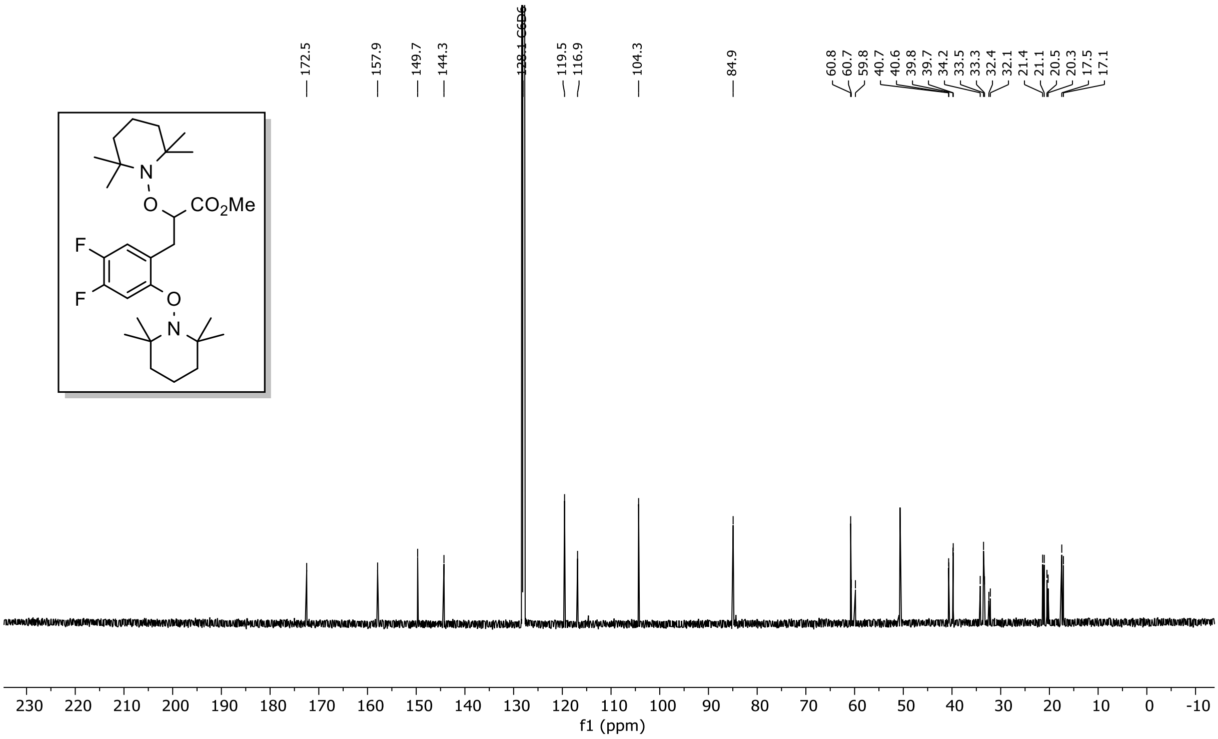

^{19}F NMR of 6f (extra small peaks are probably from [2+2] cycloaddition product)

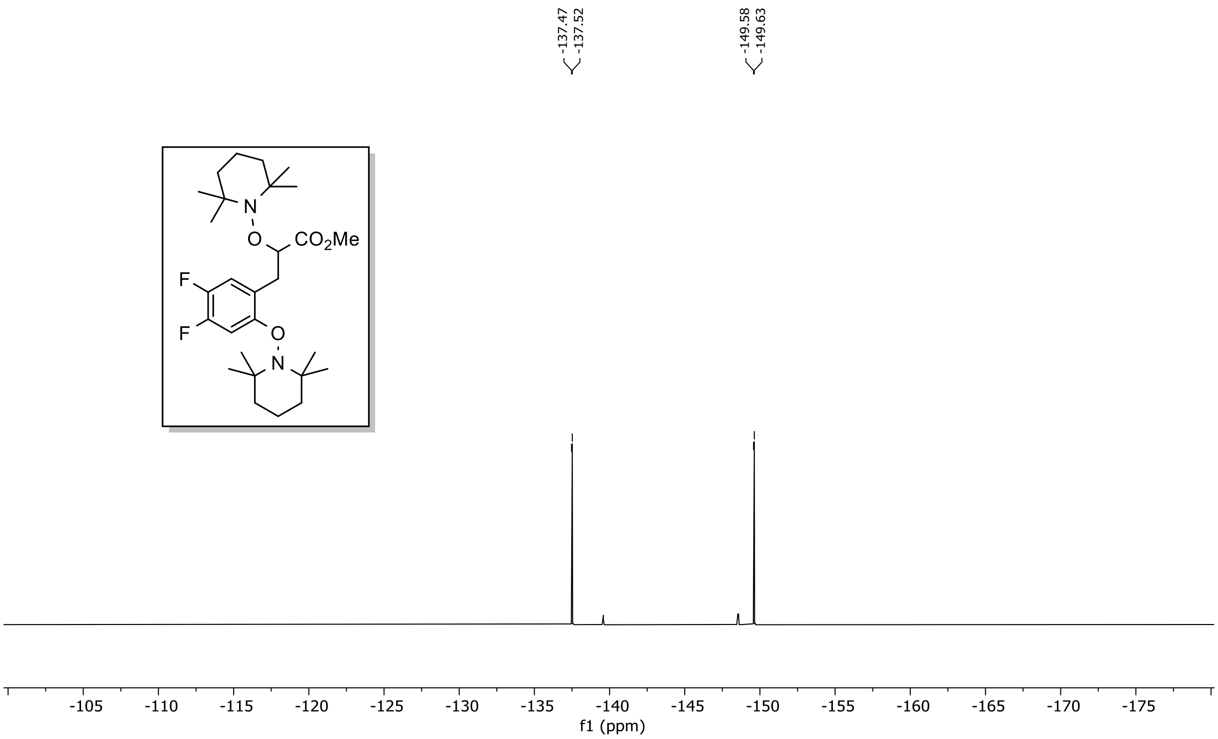

^{1}H NMR of 6g

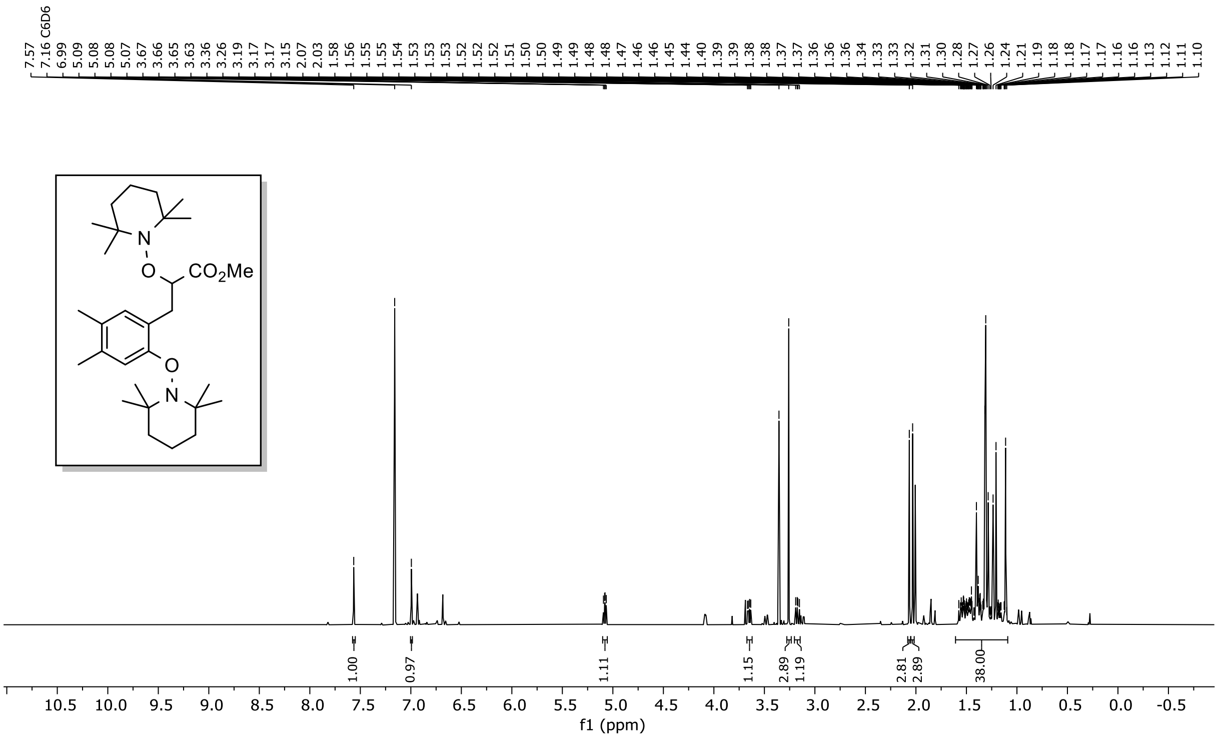

^{13}C NMR of 6g

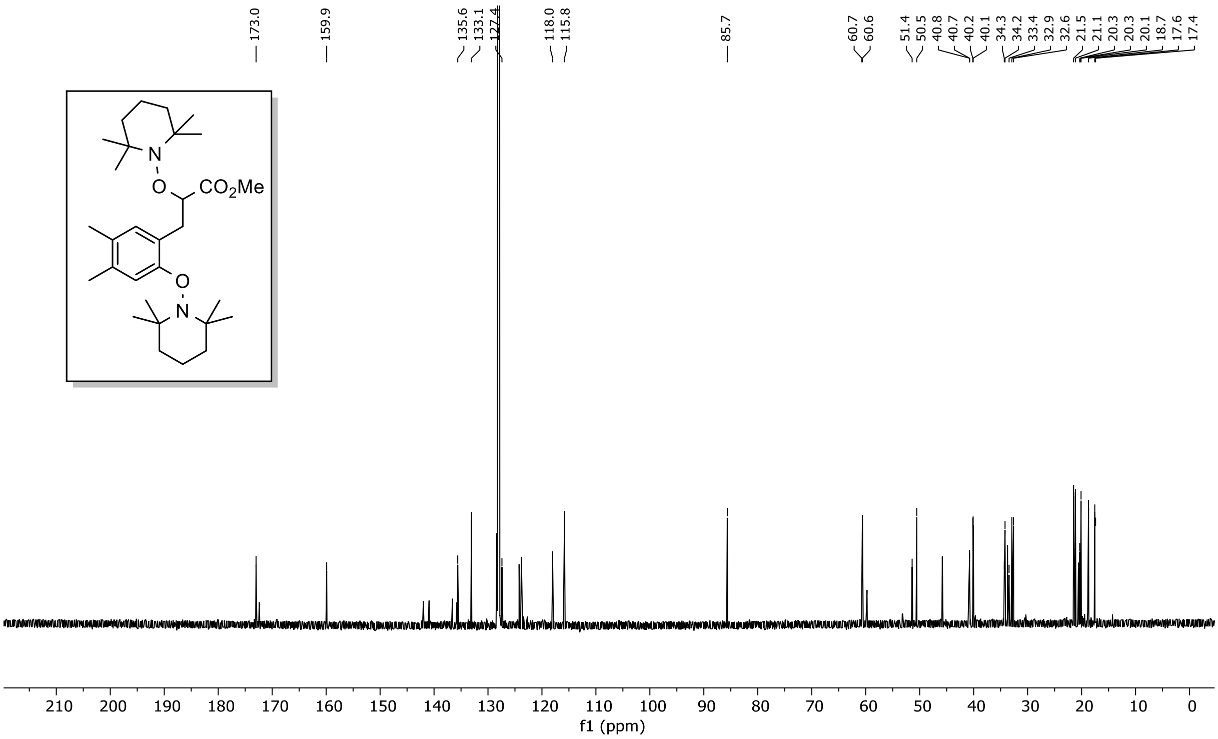

^{1}H NMR of 6g’

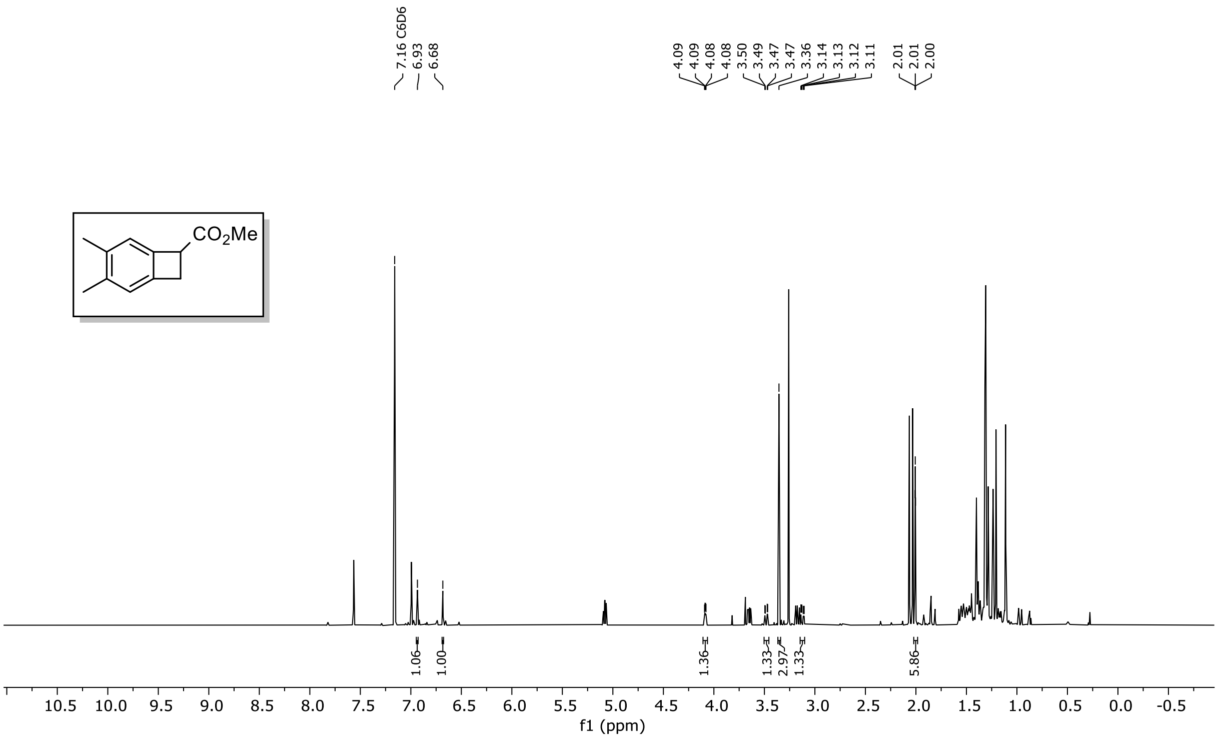

^{13}C NMR of 6g’

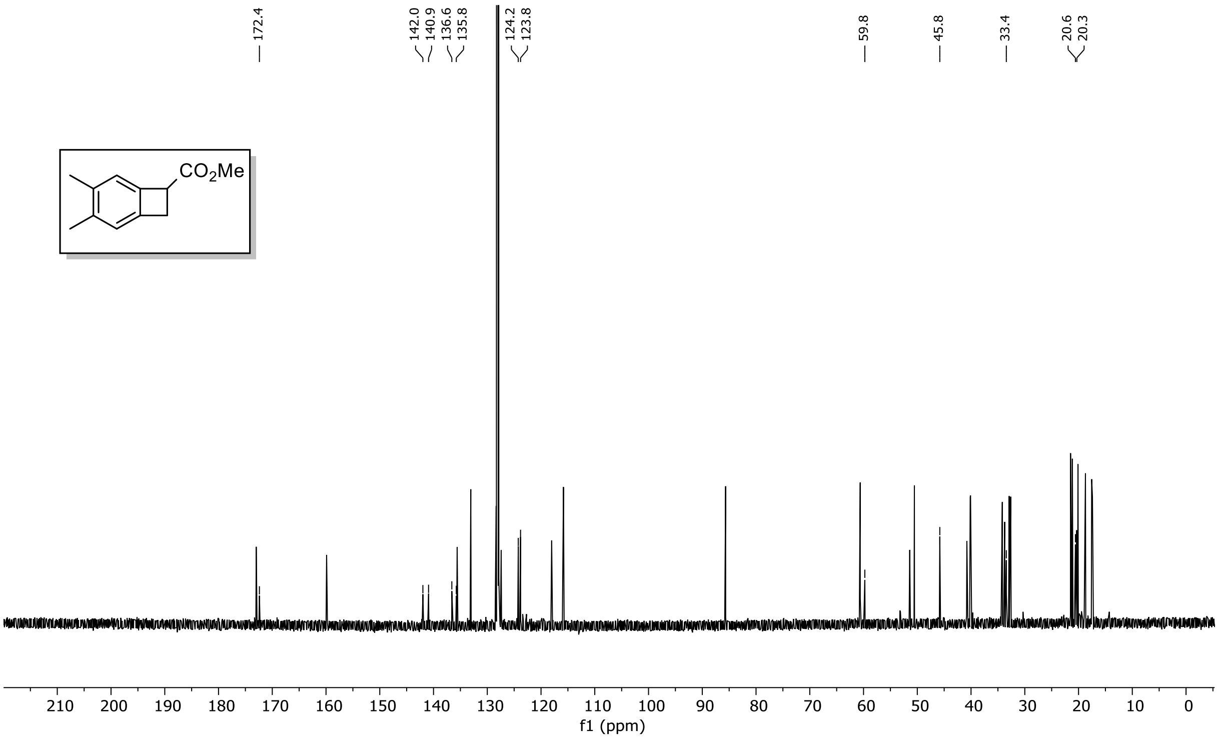

^{1}H NMR of 6h

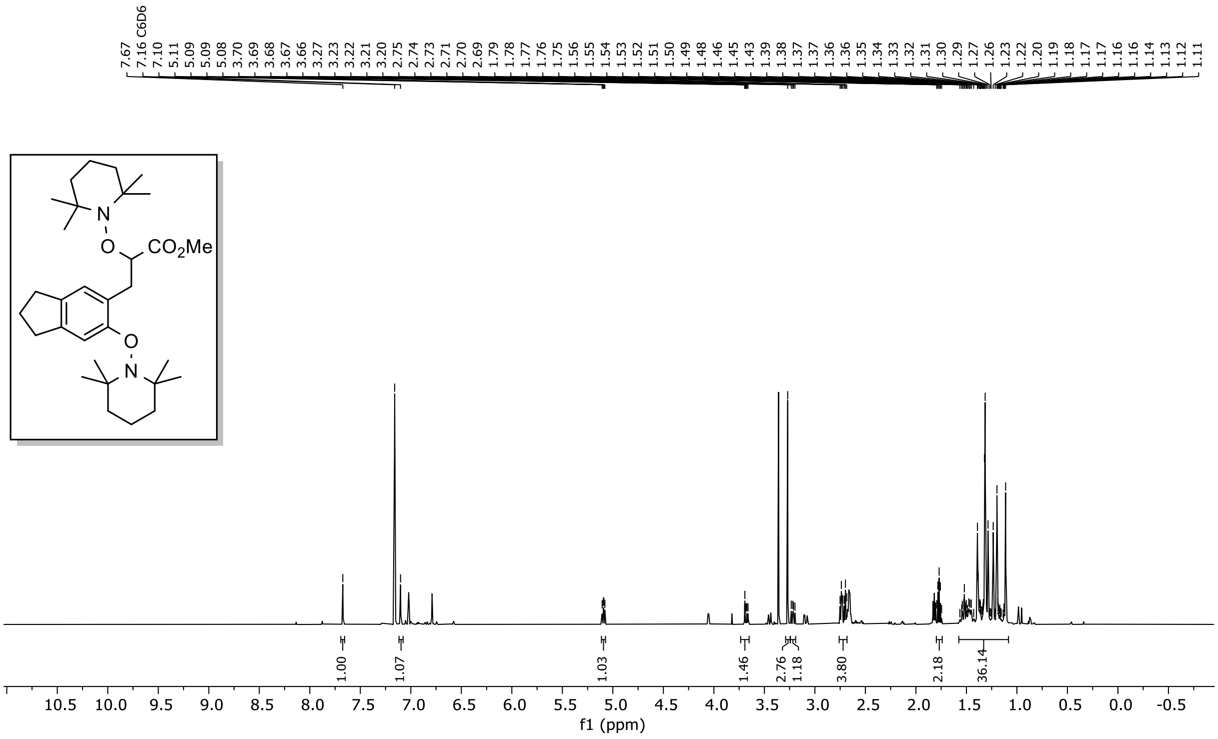

^{13}C NMR of 6h

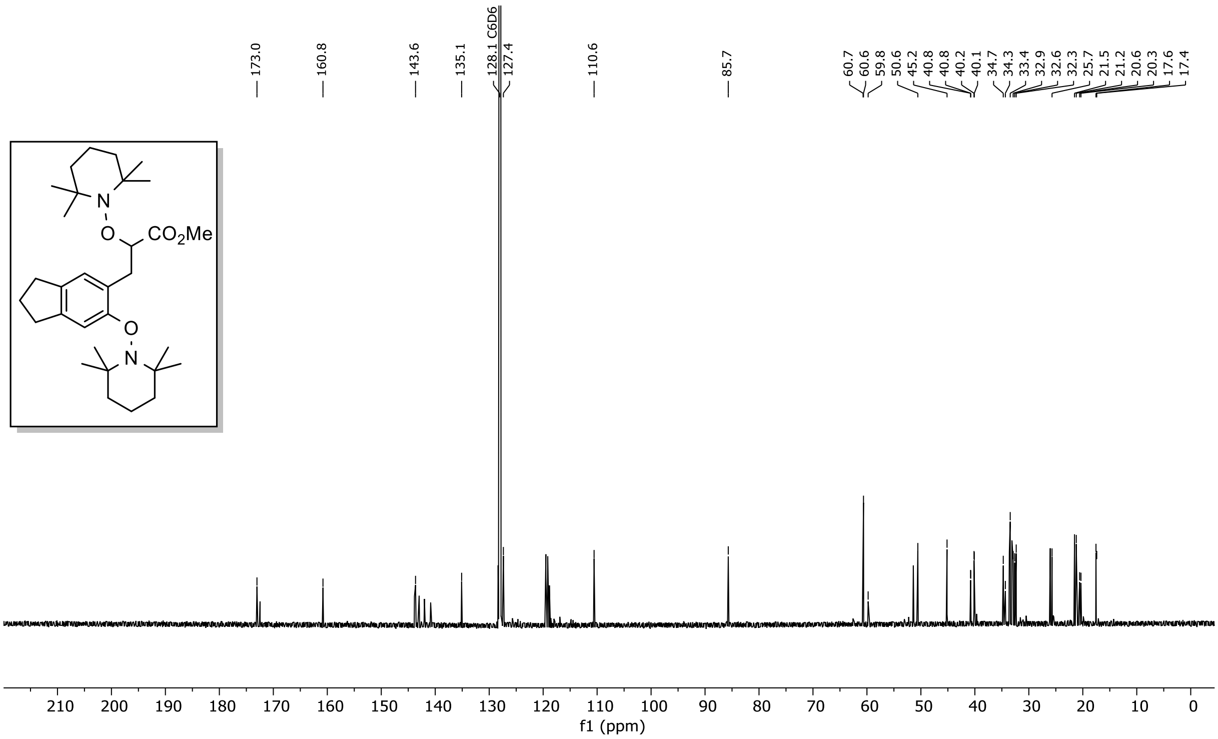

^{1}H NMR of 6h’

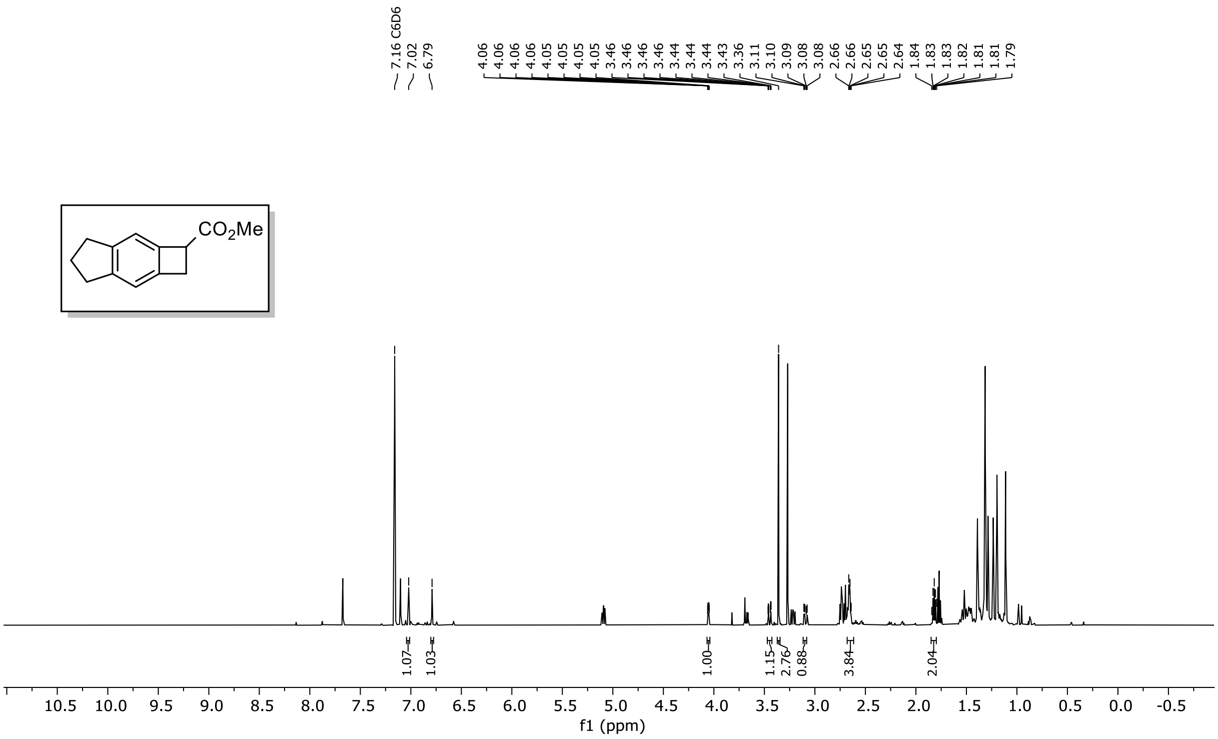

^{13}C NMR of 6h'

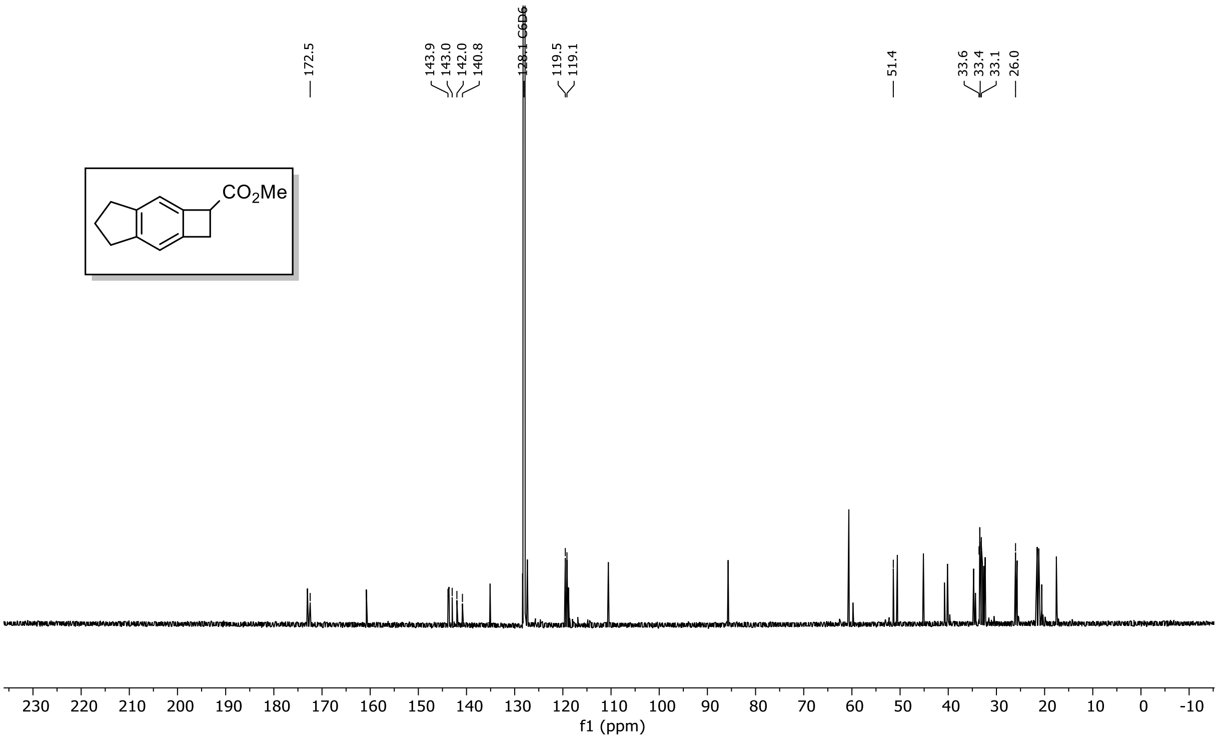

^{1}H NMR of 6i

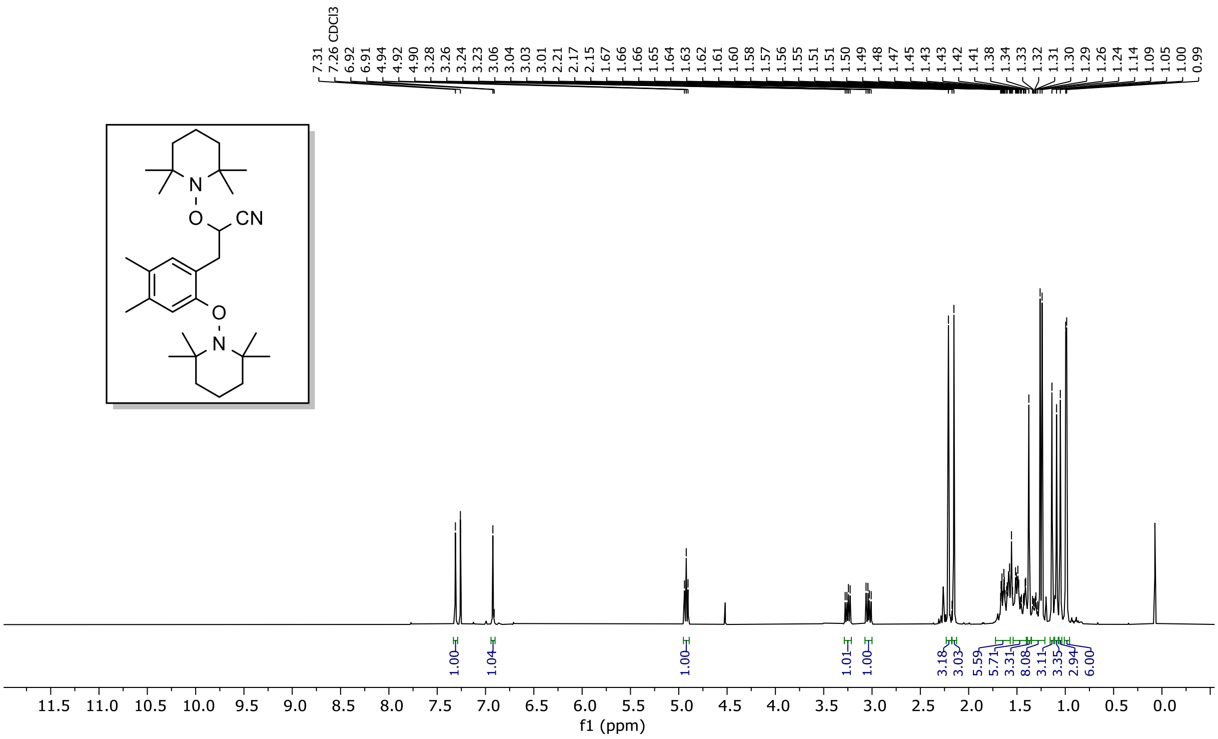

^{13}C NMR of 6i

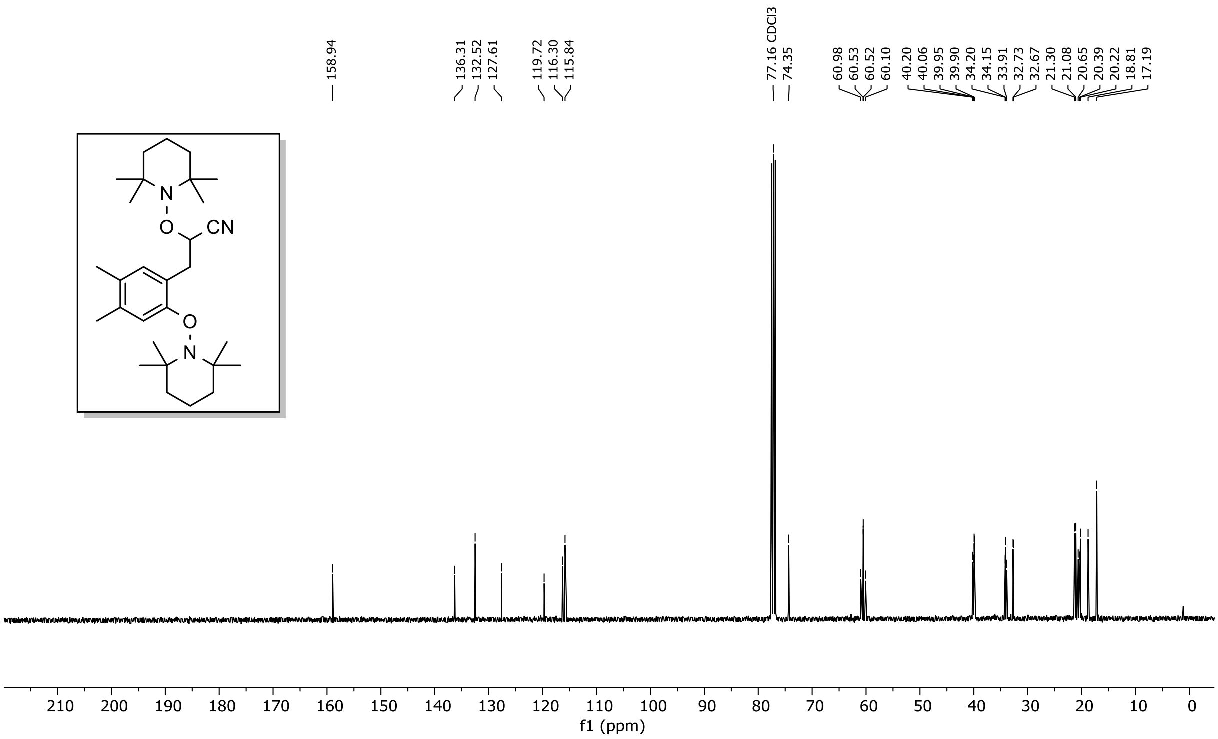

^{1}H NMR of 6j

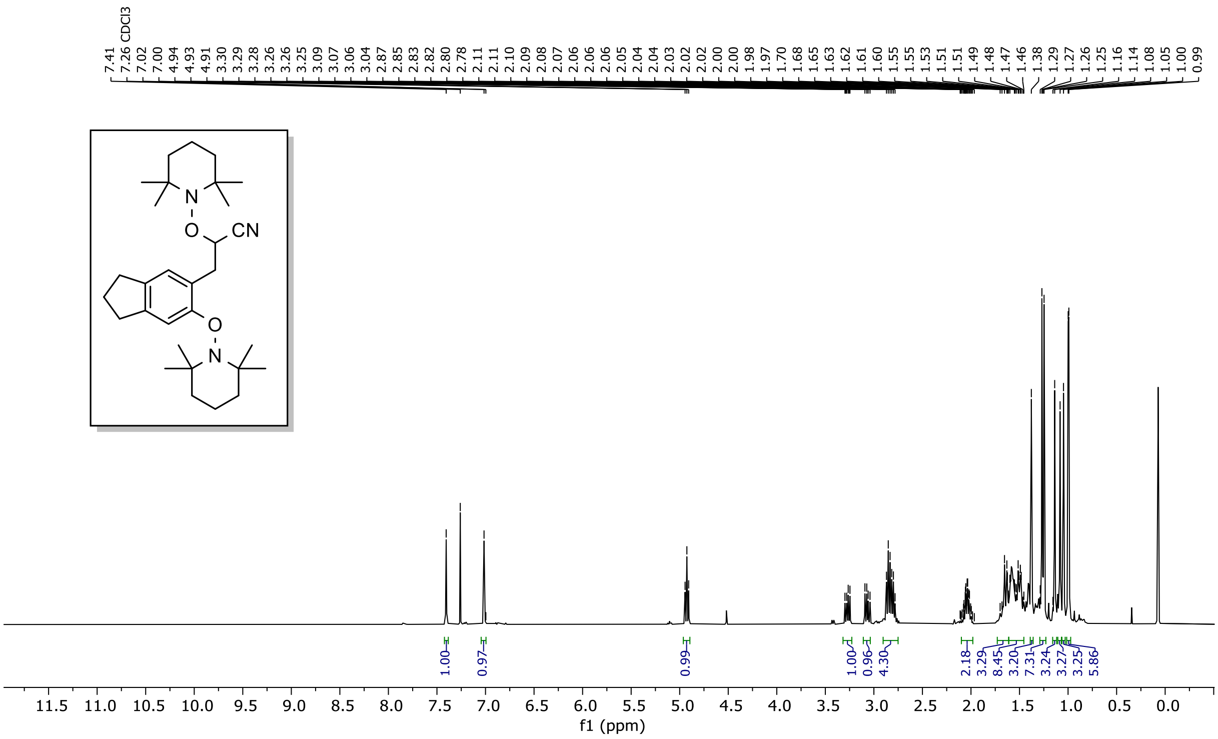

^{13}C NMR of 6j

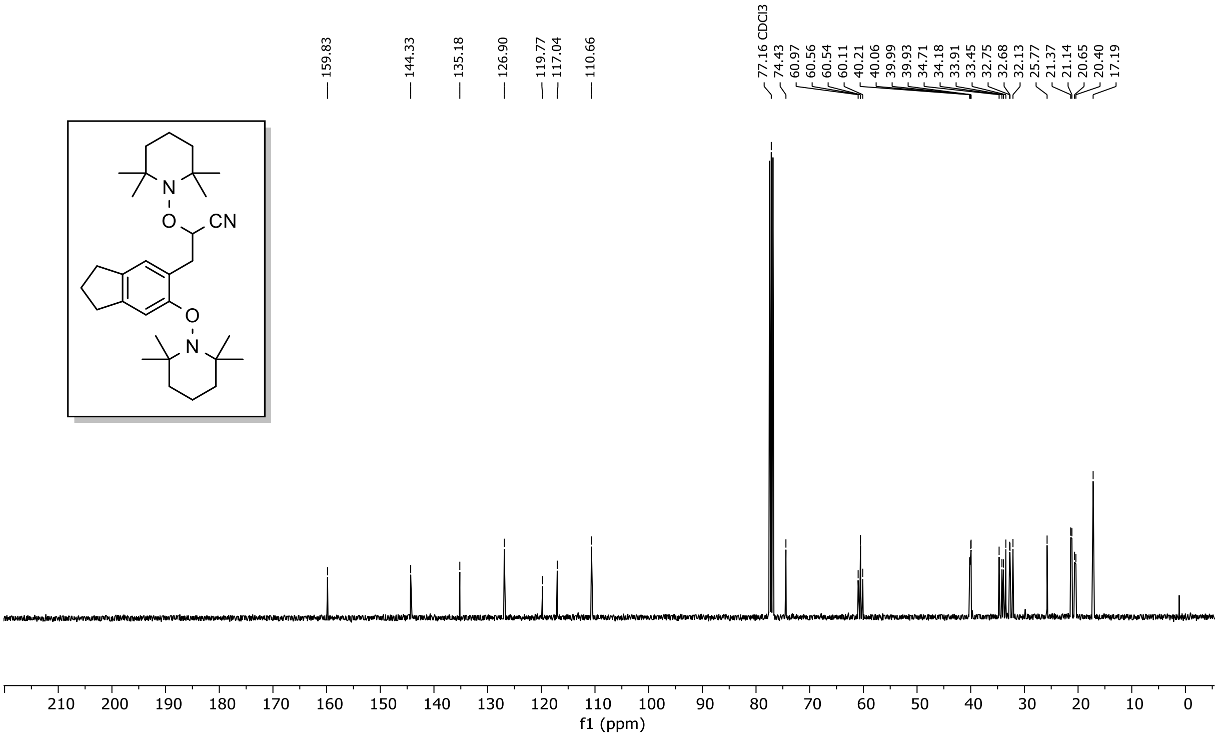

^{1}H NMR of 6k

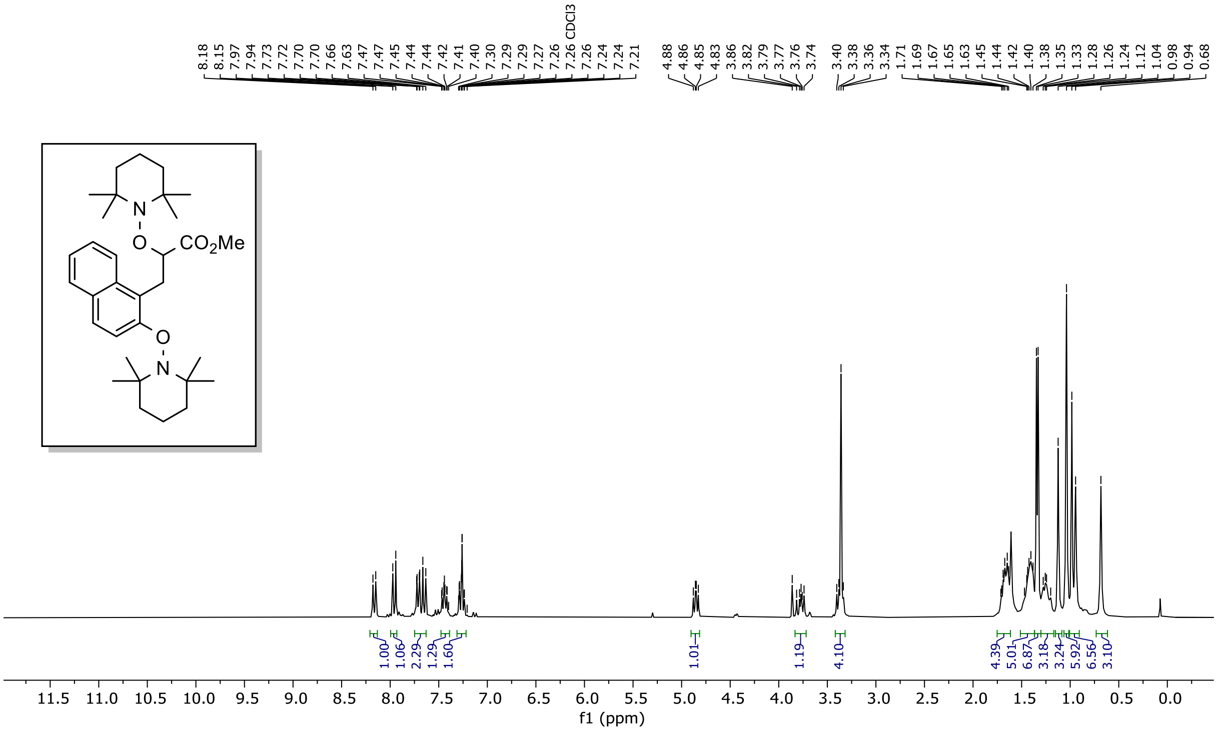

^{13}C NMR of 6k

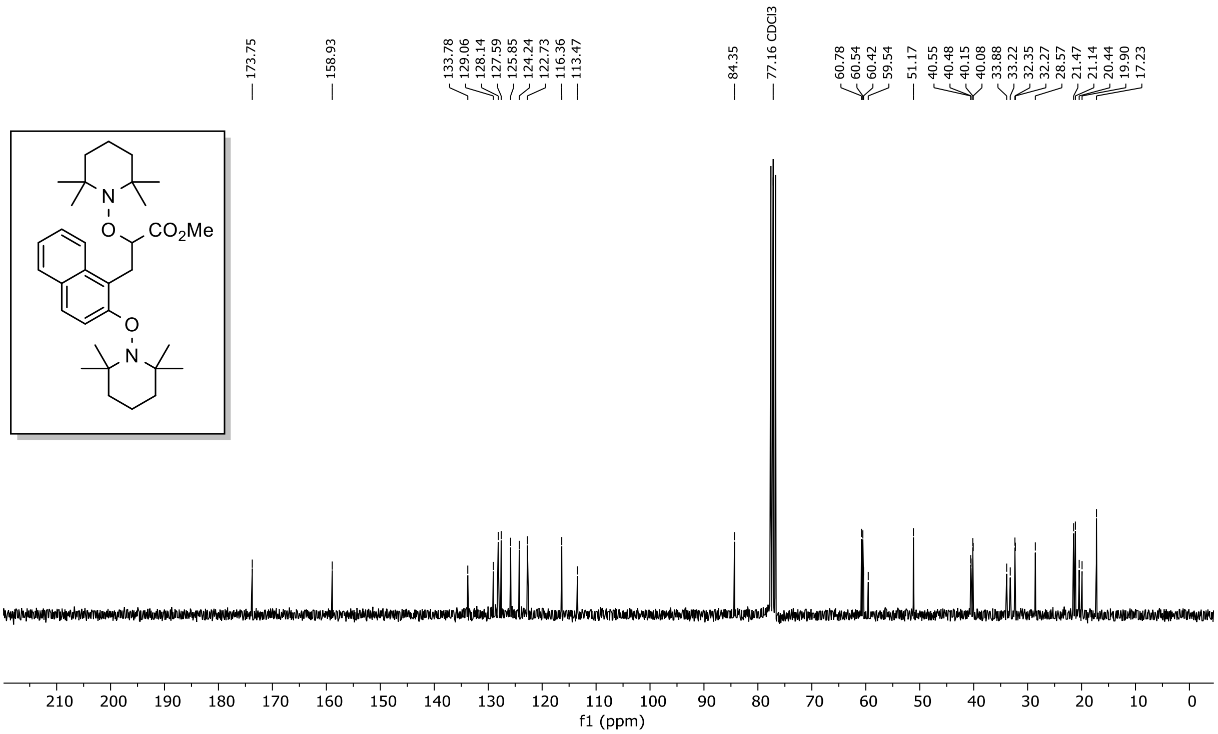

^{1}H NMR of 6l

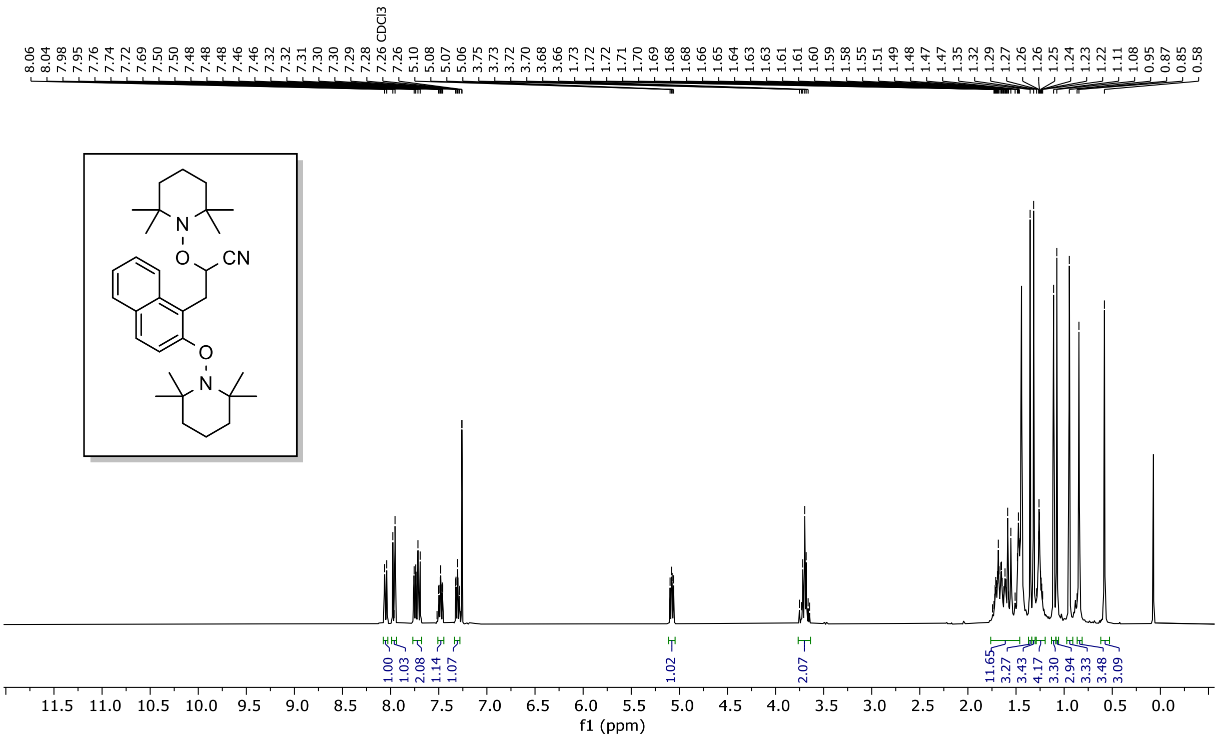

^{13}C NMR of 6l

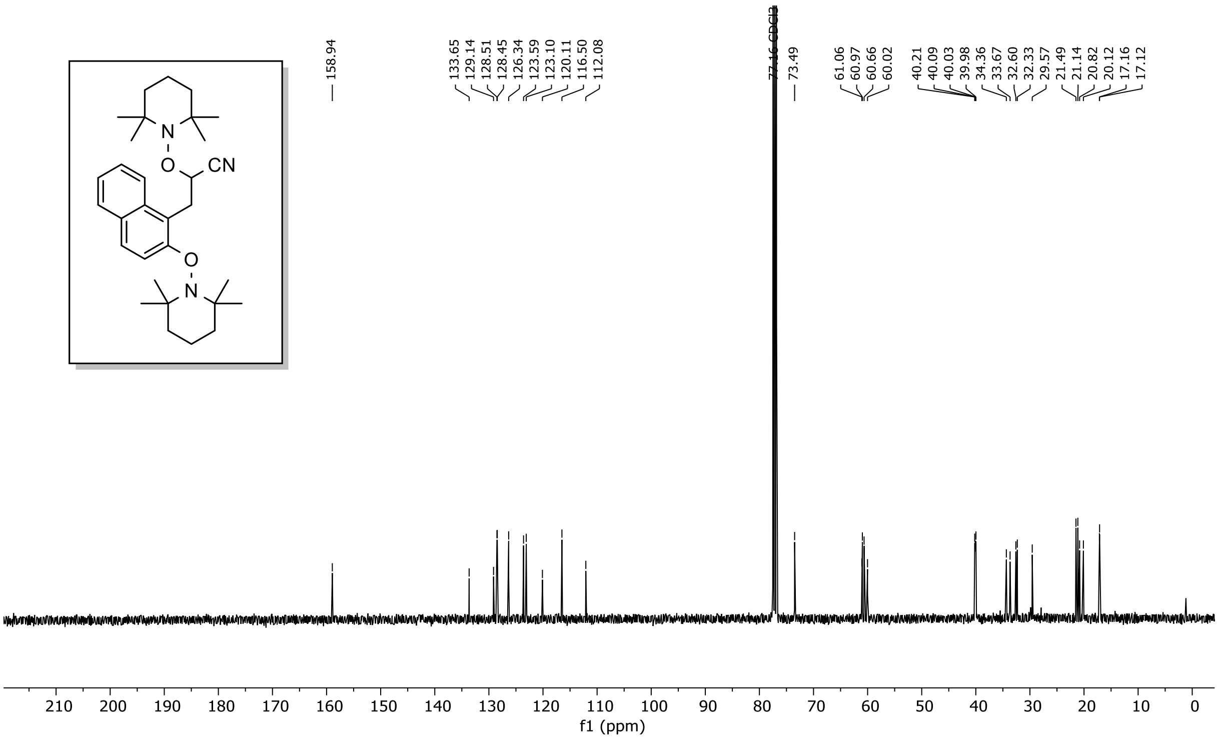

^{1}H NMR of 6m

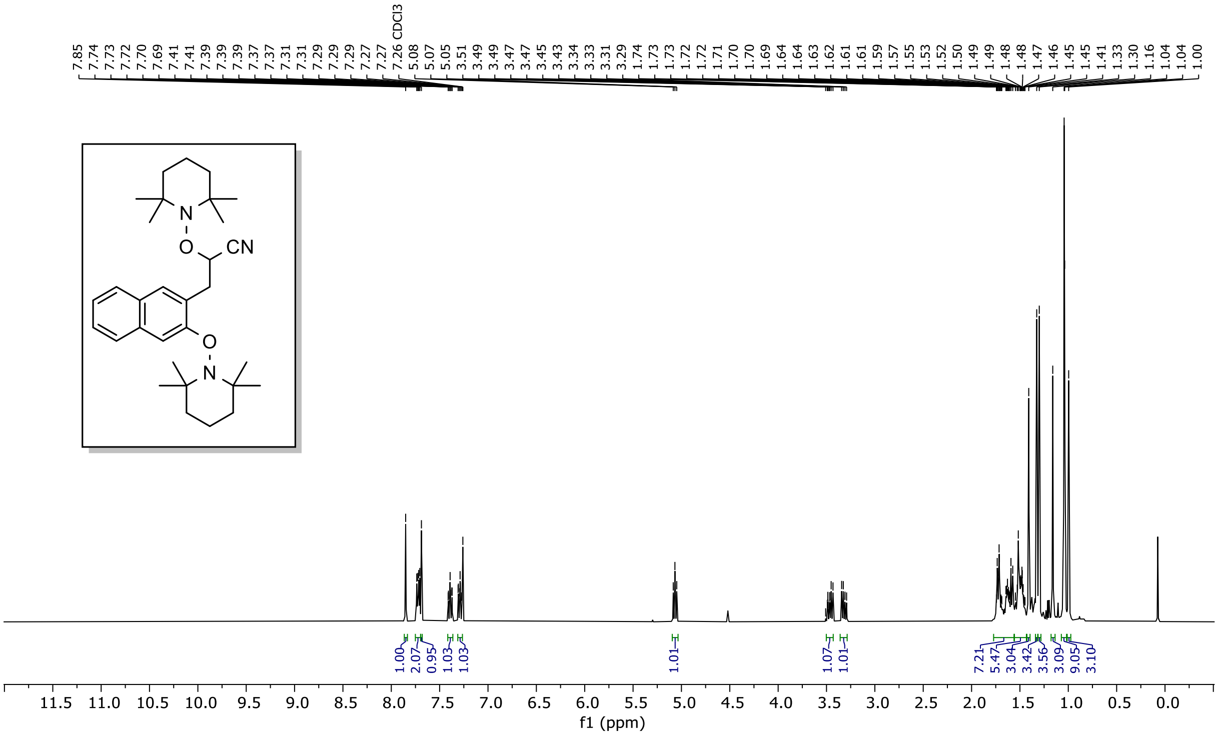

^{13}C NMR of 6m

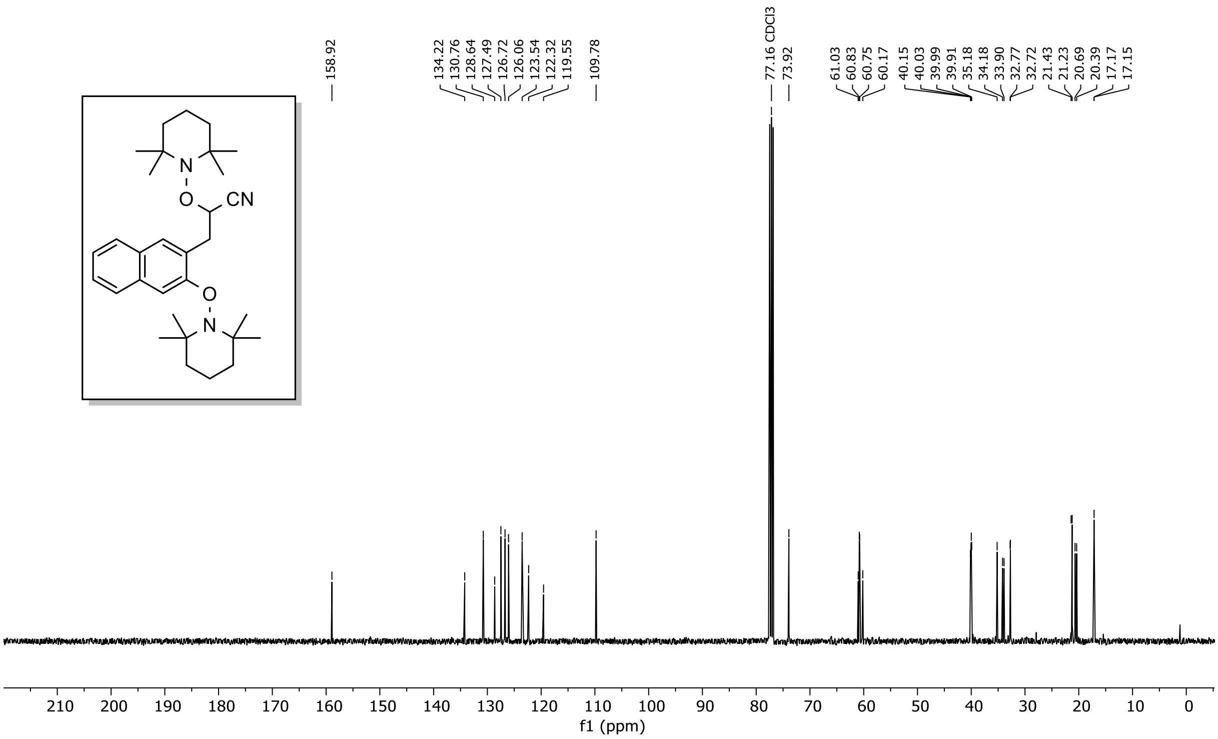

^{1}H NMR of 7

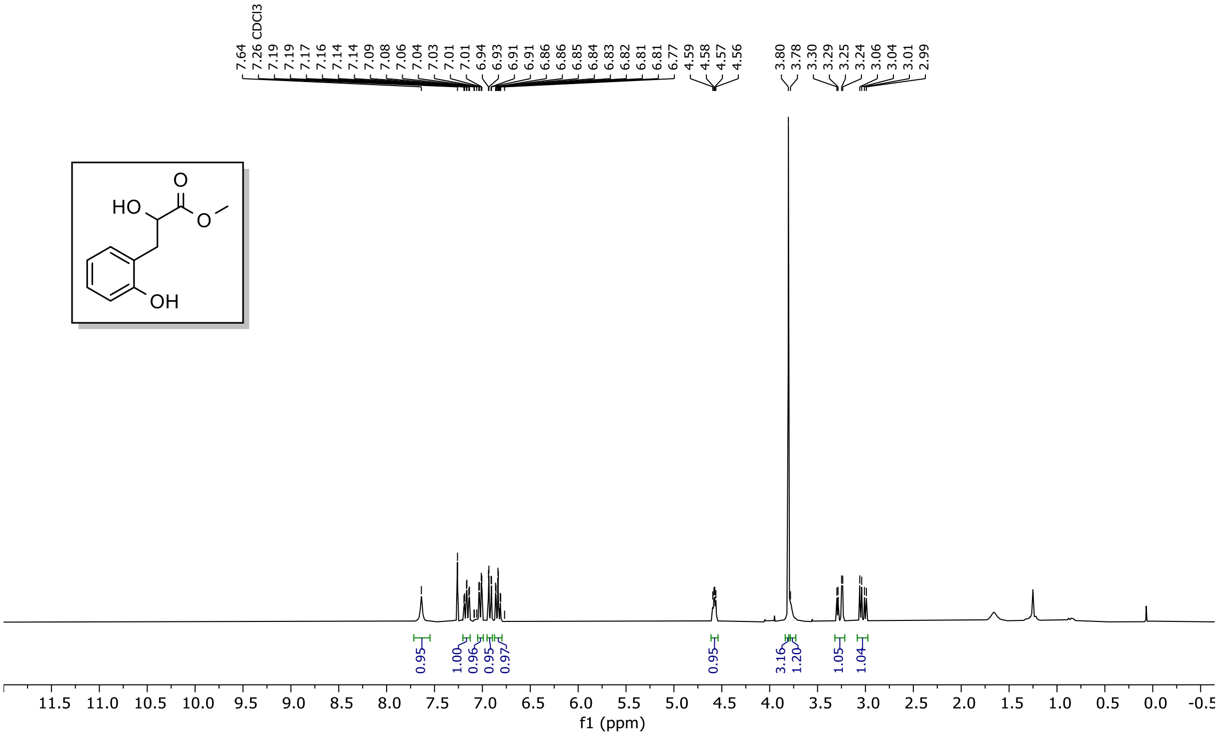

^{13}C NMR of 7

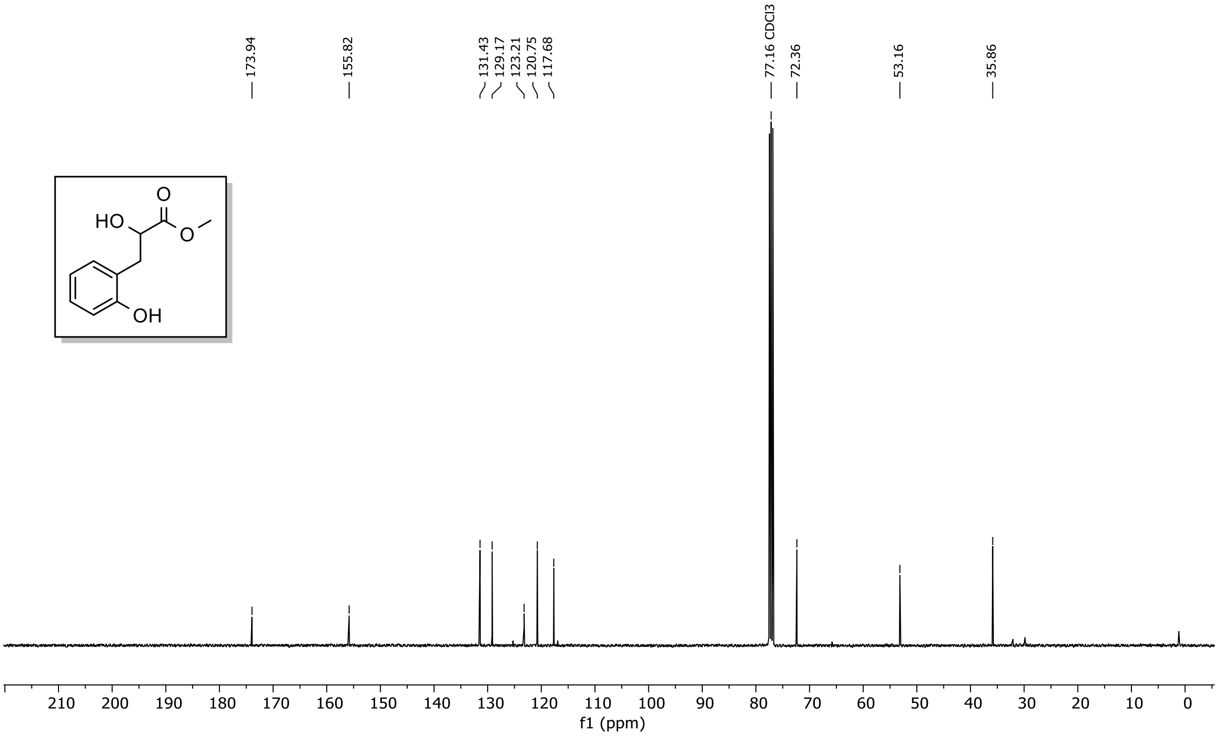

173.94
155.82
131.43
129.17
123.21
120.75
117.68
77.16 CDCl3
72.36
53.16
35.86
HO
O
O
OH
210 200 190 180 170 160 150 140 130 120 110 100 90 80 70 60 50 40 30 20 10 0
f1 (ppm)
